# Solar cycles or random processes? Evaluating solar variability in Holocene climate records

**DOI:** 10.1038/srep23961

**Published:** 2016-04-05

**Authors:** T. Edward Turner, Graeme T. Swindles, Dan J. Charman, Peter G. Langdon, Paul J. Morris, Robert K. Booth, Lauren E. Parry, Jonathan E. Nichols

**Affiliations:** 1 School of Geography, University of Leeds, LS2 9JT, UK; 2 Department of Geography, University of Exeter, EX4 4RJ, UK; 3 Geography and Environment, University of Southampton, Southampton, SO17 1BJ, UK; 4 Earth & Environmental Sciences, Lehigh University, Bethlehem, PA18015-3001, USA; 5 School of Interdisciplinary Studies, University of Glasgow, DG1 4ZL, UK; 6 Lamont-Doherty Earth Observatory at Columbia University, Palisades, NY 10964, USA

## Abstract

Many studies have reported evidence for solar-forcing of Holocene climate change across a range of archives. These studies have compared proxy-climate data with records of solar variability (e.g. ^14^C or ^10^Be), or have used time series analysis to test for the presence of solar-type cycles. This has led to some climate sceptics misrepresenting this literature to argue strongly that solar variability drove the rapid global temperature increase of the twentieth century. As proxy records underpin our understanding of the long-term processes governing climate, they need to be evaluated thoroughly. The peatland archive has become a prominent line of evidence for solar forcing of climate. Here we examine high-resolution peatland proxy climate data to determine whether solar signals are present. We find a wide range of significant periodicities similar to those in records of solar variability: periods between 40–100 years, and 120–140 years are particularly common. However, periodicities similar to those in the data are commonly found in random-walk simulations. Our results demonstrate that solar-type signals can be the product of random variations alone, and that a more critical approach is required for their robust interpretation.

Over the last 50 years there has been considerable interest in the relationship between solar variability and climate^1^,^2^,^3^. Studies from a range of sedimentary archives have investigated the role of solar forcing through comparisons of proxy climate data with reconstructions of solar activity^3^,^4^,^5^,^6^,^7^,^8^. Reconstructions of solar activity are based on concentrations of cosmogenic isotopes (e.g. ^14^C found in tree-rings and ^10^Be in ice cores) which form in the upper atmosphere and are modulated by the effects of changing solar activity on galactic cosmic ray flux^6^. Using this approach, numerous studies have reported evidence for solar-forced climate change during the Holocene epoch^3^,^5^,^9^. Furthermore, researchers have reported solar cycles in proxy climate data based on the results of spectral and wavelet analytical techniques^4^,^8^. Several papers reporting a solar-climate link have been used by climate sceptics as evidence of solar variability driving recent warming, implying that atmospheric carbon dioxide has a less important influence on global temperature^10^.

A number of climate proxies have been used in investigations of solar-forced climate change including geochemical and biological records from marine and lake sediments^3^,^5^,^11^, tree rings^12^, lake levels^13^ and glacial fluctuations^14^. In addition, palaeohydrological proxies from ombrotrophic (rain-fed) peatlands have been used to investigate Holocene solar-climate relationships^1^,^15^,^16^,^17^. Shifts in peat hydrology sometimes coincide with changes in solar activity during the mid- and late-Holocene^15^,^18^,^19^. The proposed mechanisms of solar-forced climate change include a complex series of ocean-atmosphere feedbacks driven primarily by changes in UV and solar wind^20^. The resultant variation in atmospheric circulation, temperature and precipitation would drive changes in peatland hydrology^3^,^20^. Global-scale climate response to solar forcing has also been inferred through comparison of peat profiles in Europe^1^,^15^ and N and S America^17^,^21^. In addition, spectral analysis has revealed periodicities in peat-based proxies that are similar to those found in cosmogenic isotope records of solar variability^16^,^19^,^22^. These periodicities have been frequently interpreted as periodic changes in climate, reflecting multi-decadal to centennial solar cycles^22^.

However, Holocene climate proxies are noisy and have chronological errors that often lead to considerable temporal uncertainties in reconstructions^7^,^23^. Quasi-random variations that arise from complex, non-linear autogenic fluctuations can themselves cause ecosystem changes including abrupt events, long-term trends and even quasi-cyclic behaviour^24^. Climate reconstructions derived from biological proxies in ombrotrophic peatlands rely on the assumption that down-core changes in species composition are driven by climate variability^25^. Whilst there is often ample evidence to suggest that hydrology is the strongest environmental control on taxa used in reconstructions (e.g. testate amoebae), other factors, such as competition, pH and trophic status may also play an important role^26^. We address the question of whether periodicities found in peat-based palaeoclimate records truly reflect changing solar activity, or whether they could also be explained by random variations or artefacts of sampling intervals and/or chronological errors.

We examined nine high-resolution proxy climate records from ombrotrophic bogs in Europe and the USA (Fig. 1, Supplementary Methods S1). These proxy records have high quality age control and robust age-depth relationships based on Bayesian models (Supplementary Fig. S2). Spectral and wavelet analyses were used to identify solar-type signals in the peat record, while the sunspot reconstruction of Solanki *et al.*^27^ was used as the record of changing solar activity through the mid-late Holocene. We also developed random walk simulations (RWs) – a non-stationary stochastic ‘red noise’ time series where values wander randomly over time (ref. 28; Supplementary Fig. S3). These simple simulations can exhibit complex features such as those found in palaeoenvironmental data^24^. We sampled fifteen RWs per site at the same time interval as the real proxy data to see if similar periodicities could be found in random simulations. We also generated an additional 5000 RWs sampled to a regular time interval of 10 years which we tested for significant positive correlation with the solar record. We used these to test a null hypothesis that such variations are the product of random variations. We selected one RW per site with features that plausibly imitate ‘real’ proxy reconstructions, such as rapid changes and quasi-cyclic patterns, for further detailed statistical analysis to illustrate our argument.

There are well-established climatic events in some of the peat-based records including the 2.7 ka BP year event, Medieval Warm Period, and the Little Ice Age (Fig. 1). The records indicate that rapid change in the last ~100 years is coincident with both the large increase in global atmospheric CO_2_ concentration and a rise in sunspot numbers. There are periods in the record where shifts in the proxy climate data correspond with excursions in solar activity (Fig. 1). There are also significant correlations between the proxy records from four of our nine sites and the solar reconstruction (Supplementary Table S7). Many previous studies have used running correlation analyses between records of solar variability and proxy climate data time series to interrogate the relationship between solar forcing and Holocene climate change^11^,^29^. Our analysis (Supplementary Fig. S7) shows that the running correlations between the proxy climate records and solar variability are highly variable in time for both 100-year and 500-year windows; however, when an appropriate Monte Carlo significance testing procedure is used (Supplementary Data S8) it is mostly non-significant (*p* > 0.10). Some studies have utilised significance testing procedures that are not appropriate for time series data as they do not account for the multiple comparison problem^11^,^29^. There are also significant correlations and running correlations between the RWs and the solar record, four of which are similar to or even stronger than those found for the ‘real’ data (Supplementary Fig. S7). Interestingly, 45% of the 5000 RWs were positively correlated with the solar record (Supplementary Fig. S9). Given that these are purely random data, it is quite remarkable that nearly half of these RWs show this level of correlation. This poses the question of whether solar-type cycles in proxy climate records can be robustly linked to solar variability.

Spectral analysis shows that there are a large number of significant, high-frequency periodicities present in the real data (Fig. 2). Commonly occurring periodicities span the ranges 40–100 years (n = 113 > 90% false alarm level), and 120–140 years (n = 17 > 90% false alarm level). In addition, our analysis of previous studies has shown the prominence of 80–90, 130–140, 200–210 and 260–270 year periodicities in peat-based climate records (Supplementary Table S6). However, caution is needed when interpreting these results as there may be a publication bias: the focus of several of these studies was to present evidence for solar-forcing of Holocene climate. Low-frequency periodicities were also present in both the real and RW data (Fig. 2), but millennial-scale climatic changes may be poorly preserved in peatlands due to signal-shredding or over-writing by autogenic processes such as ecohydrological feedbacks and secondary decomposition^25^. Additionally, the maximum time period covered by the peat cores in this study is 7 k years, rendering millennial-scale periodicities more questionable.

The periodicities reported here and in previous studies are present in the solar reconstruction (Fig. 3A) and match the range of the Gleissberg cycle (~70–100 years) and sub-harmonics of the Hale cycle (~132 years)^30^, de Vries cycle (~200–210 years) and others present in the ^14^C record (105, 131, 232, 385, 504, 805, 2,241 years: ref. 31). These cycles have also been shown to be prominent in other Holocene proxy climate records^9^,^16^. However, similar significant periods are also found in the analysis of RWs (Fig. 2). Periods similar to solar cycles are particularly common: 80–160 years and a clear peak at 120–140 years. Another peak spanning 200–220 is present (Fig. 2) that matches exactly the period of the de Vries solar cycle. Interestingly, 200–220 year periods are mostly absent from the real proxy climate data. Wavelet and Cross-Wavelet analyses illustrate clearly that any relationships between solar variability and the proxy climate records are temporally variable, inconsistent between records, and show phases of correspondence and non-correspondence. These discrepancies seem likely to result from some combination of: i) the sensitivity of a proxy to climate drivers; ii) differences in temporal resolution within a record driven by changes in sedimentation rate; and/or iii) differences in sampling resolution between reconstructions (Supplementary Fig. S5). The lack of consistency in correspondence through time and between sites is clear, suggesting that either the sites have exhibited variable sensitivity to solar-forced climate change over time, or that solar variability is not driving the variability in the proxy data (Fig. 3, Supplementary Fig. S5).

Periodicities present in proxies derived from complex environmental systems must be interpreted with caution because such systems possess the potential to modify external (climatic) signals through autogenic mechanisms (e.g. ref. 32, for sedimentary systems). Peat-based proxy climate records can exhibit amplified, damped or phase-shifted representations of climatic influences through mechanisms such as vegetation succession^33^ and a range of negative feedback mechanisms that can lead to a degree of homeostasis in system behaviour^25^,^34^.

The most common significant periodicities found here (within the ranges 40–100 years and 120–140 years) could be interpreted as evidence for solar-forced climate change because they match the ranges of cycles in solar reconstructions. However, similar periodicities are also prominent in the random-walk simulations. Thus, we propose that many of the periodicities found are the product of either: i) random variations; ii) autogenic mechanisms in a complex environmental system; iii) the sampling resolution; iv) the age model applied; or v) some combination of the above factors. Our analysis illustrates the importance of replication to avoid erroneous attribution of periodicities to external forcing. Large ensembles of well-dated Holocene proxy climate data are necessary for robust testing of solar signals in Holocene proxy climate records^16^,^35^, because they filter local, non-climatic effects and reveal persistent variations, some of which may well be associated with past solar variability. In dealing with time series analysis, care should be taken when attributing cyclical behaviour to solar forcing because such signals could merely be the product of random variations, non-climatic (e.g. autogenic) factors or the temporal-expression of the sampling strategy. We contend that many solar-type cycles reported in the palaeoclimatological literature may potentially be artefacts.

## Method

**Figure 1:**
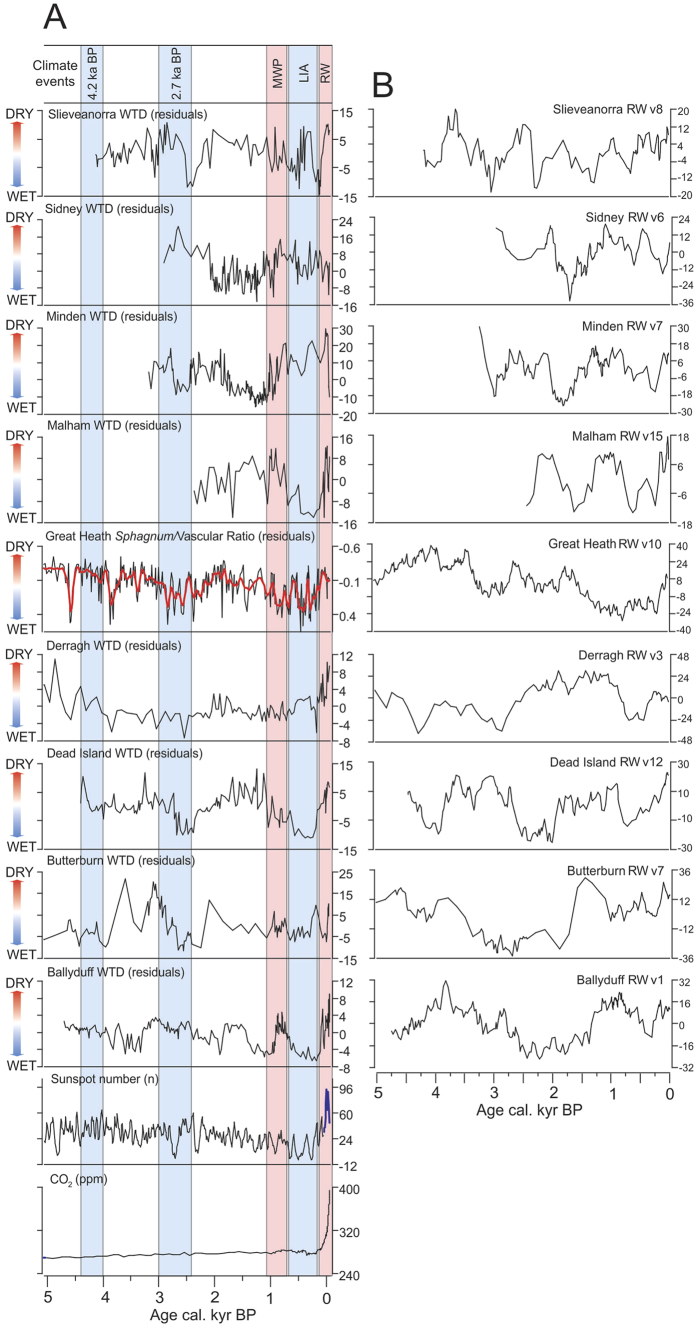
(**A**) Normalised water-table reconstruction from Ballyduff, Derragh, Dead Island, Slieveanorra (Ireland), Butterburn and Malham (England), Minden and Sidney (USA). The record from Great Heath (USA) is *Sphagnum*/Vascular Ratio based on ratios of leaf wax compounds. A loess smoothing function is illustrated (red line). The chronologies have been modelled using a Bayesian statistical approach (Supplementary Fig. S2). Reconstructed sunspot numbers (Solanki *et al.*^27^) and sunspot counts (blue line; source: SILSO data/image, Royal Observatory of Belgium, Brussels), and the combined CO_2_ record from Mauna Loa, the Law Dome and EPICA Dome C ice cores (See refs in Supplementary Method S1). (**B**) An example random walk simulation for each site (sampled to the same chronological spacing as the real data) is also shown.

We examined nine high-resolution proxy climate records from ombrotrophic bogs located in the Northern Hemisphere (USA and Europe; Fig. 1, Supplementary Methods S1). Eight of these records are based on transfer function-reconstructions of water-table depth from testate amoebae microfossils in the peat and one is based on *Sphagnum*/Vascular Ratio determined through ratios of leaf wax compounds (see Supplementary Methods S1 for full details). Age-depth models for the proxy palaeohydrological records were generated from radiocarbon dates and age-equivalent stratigraphic markers (tephra, spheroidal carbonaceous particles) using a Bayesian statistical modelling approach. A series of 15 random walks per site were generated (based on each dataset) and time-steps were matched to the corresponding proxy (e.g. Dead Island = 4454 years) from an initial value of zero. The sunspot reconstruction of Solanki *et al.*^27^ was used as the record of changing solar activity through the mid-late Holocene. Spectral and wavelet analyses were used to determine periodicities in the data, and cross-wavelet analysis was used to determine the temporal relationship between the proxy data and the sunspot reconstruction. The significance of periodicities was tested against appropriate noise background models. Bivariate running correlation analysis (time windows = 100 and 500 years) was used to determine the correlation between the solar record and the proxy climate data and the temporal variation of the correlation. The statistical significance of the correlation was calculated using a Monte Carlo simulation to determine the null distribution. An additional 5000 random walks were generated and tested for significant positive correlation (Spearman’s Rank, *p* < 0.05) with the solar reconstruction^27^. For full methods see Supplementary Methods S1.

## Additional Information

**How to cite this article**: Turner, T. E. *et al.* Solar cycles or random processes? Evaluating solar variability in Holocene climate records. *Sci. Rep.* **6**, 23961; doi: 10.1038/srep23961 (2016).

## Supplementary Material

Supplementary Information

## Figures and Tables

**Figure 2:**
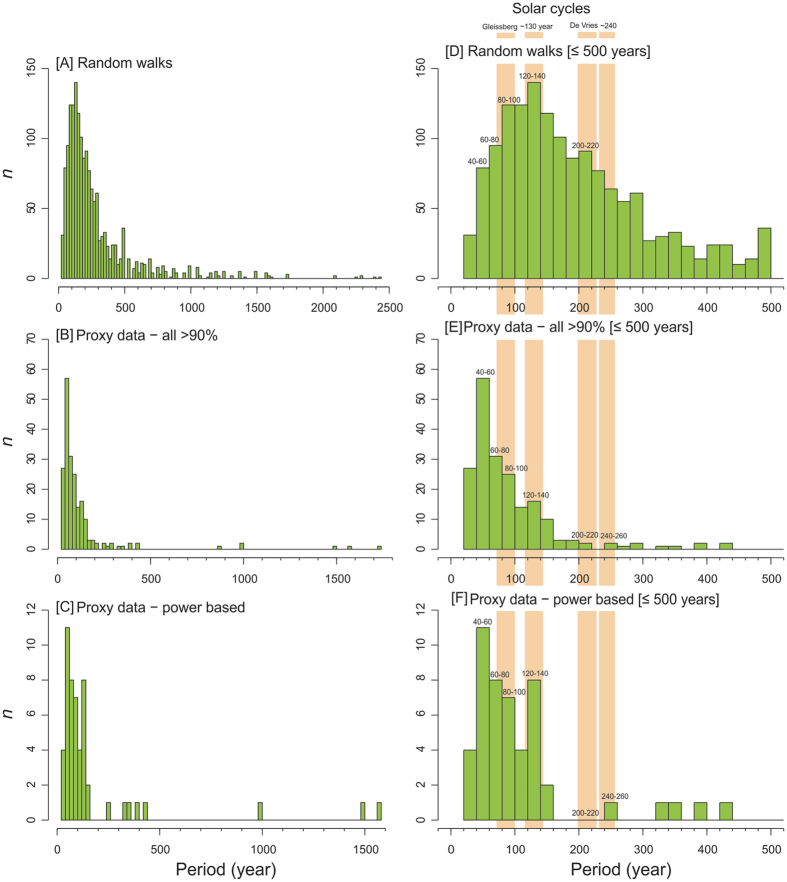
Histograms of significant periodicities present in the data and random walk simulations. (**A**) All periodicities in the random walks over 90% false alarm level; (**B**) All periodicities in the proxy climate records over 90% false alarm level; (**C**) Highest power periodicities in the proxy climate records over 90% false alarm level; (**D**) Periodicities with a period ≤500 years in random walks over 90% false alarm level; (**E**) Periodicities with a period ≤500 years in the proxy climate records over 90% false alarm level; (**F**) Highest power periodicities in the proxy climate records over 90% false alarm level ≤500 years. Solar cycle bands commonly reported in palaeoclimate literature are illustrated.

**Figure 3:**
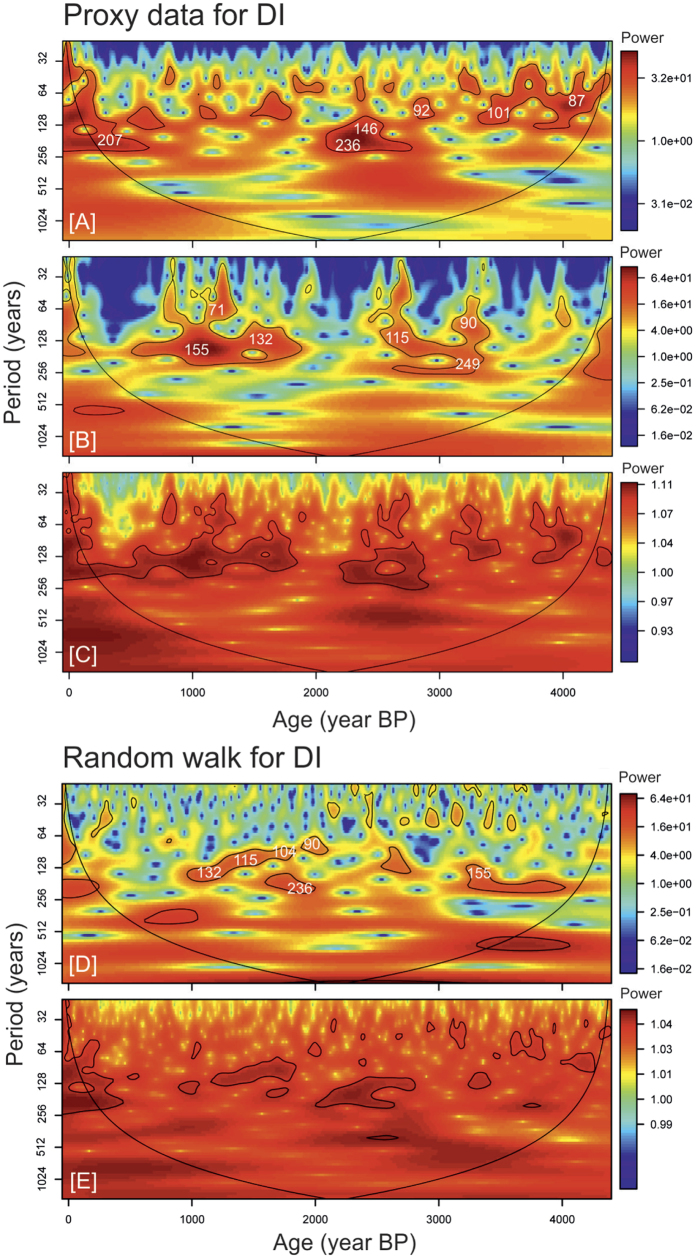
Continuous wavelet analysis of (A) the sunspot reconstruction of Solanki *et al.*^27^; (B) normalised water table reconstruction from Dead Island; (C) Cross-wavelet analysis of (A,B); (D) Random walk simulation sampled to the same chronological spacing as Dead Island; (E) Cross-wavelet analysis of (A,D). The black lines signify 95% significant levels against a lag1 (red noise) background. Dead Island is given here as an example: for other sites refer to Supplementary Fig. S5.
