# Corrigendum: Solar cycles or random processes? Evaluating solar variability in Holocene climate records

**DOI:** 10.1038/srep28410

**Published:** 2016-06-27

**Authors:** T. Edward Turner, Graeme T. Swindles, Dan J. Charman, Peter G. Langdon, Paul J. Morris, Robert K. Booth, Lauren E. Parry, Jonathan E. Nichols

Scientific Reports 6: Article number: 2396110.1038/srep23961 published online: 04052016; updated: 06272016.

A coding error in the Monte Carlo procedure for the determination of critical values in running correlation analysis (presented in Supplementary Data S8) has been brought to the attention of the authors. The code should read:


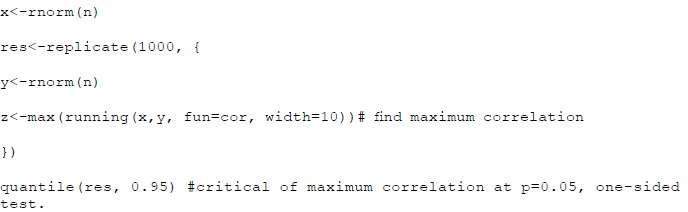


This is for 100-year time windows, and as the data has a 10-year time step, width = 10; n is the number of data points in the time series. In each case this makes a relatively minor change to the critical levels shown as dashed lines in Supplementary Fig. S7. The corrected critical levels are provided in the Table 1 below. In addition, the correct Figures appear below as Figures 1, 2, 3, 4, 5, 6, 7, 8, 9, 10.

As evident, the statement in the paper that most running correlations are mostly insignificant still holds entirely true. The authors would also like to acknowledge Richard Telford’s blog article which contained code providing the foundation for this small component of our analysis: (https://quantpalaeo.wordpress.com/2013/01/04/running-correlations-running-into-problems/).

## Figures and Tables

**Figure 1:**
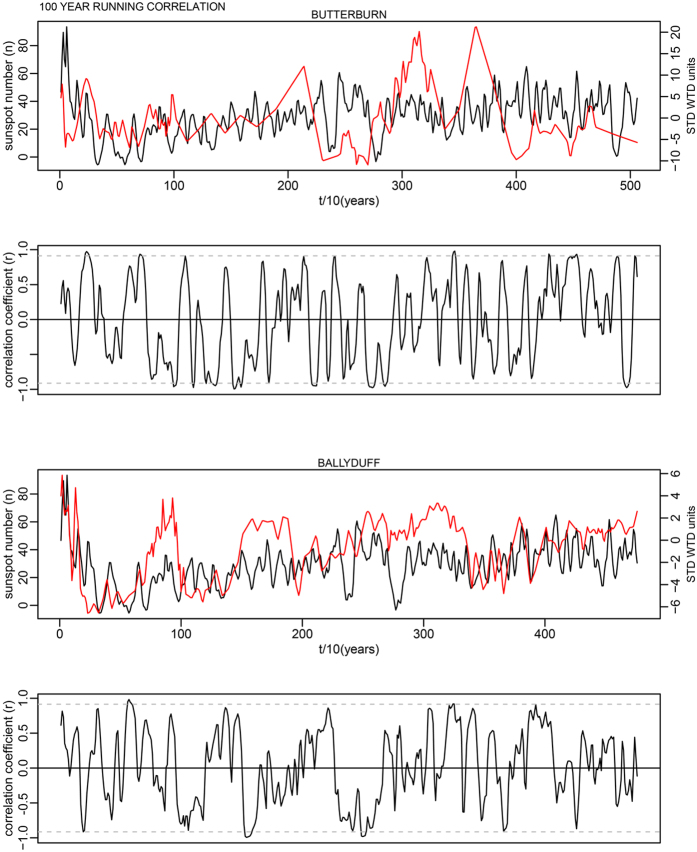


**Figure 2:**
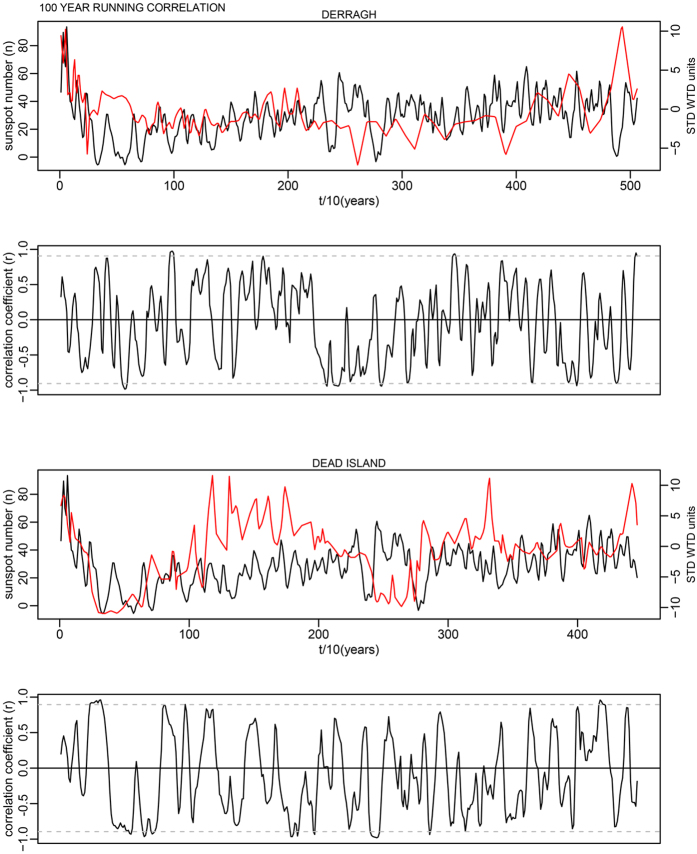


**Figure 3:**
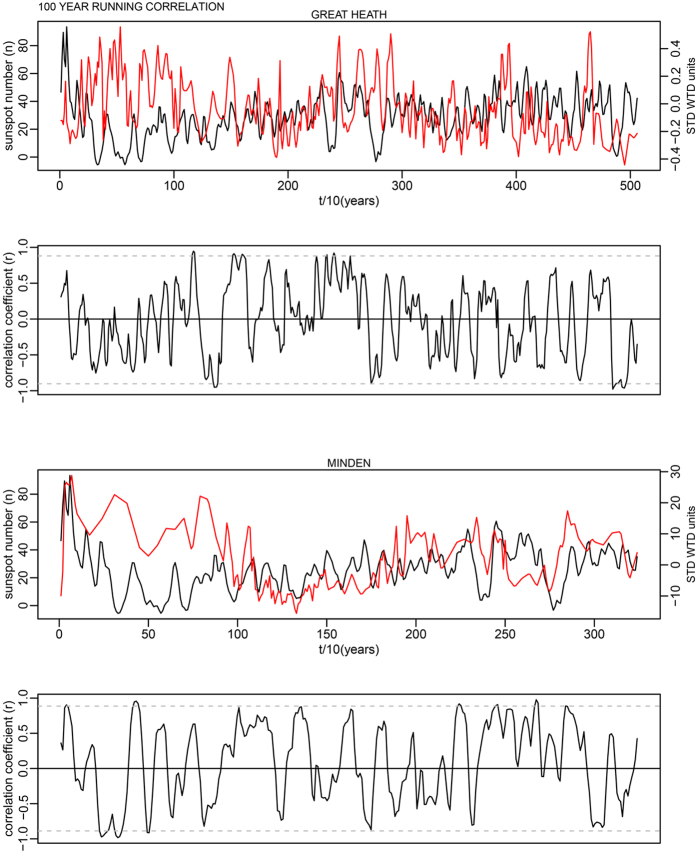


**Figure 4:**
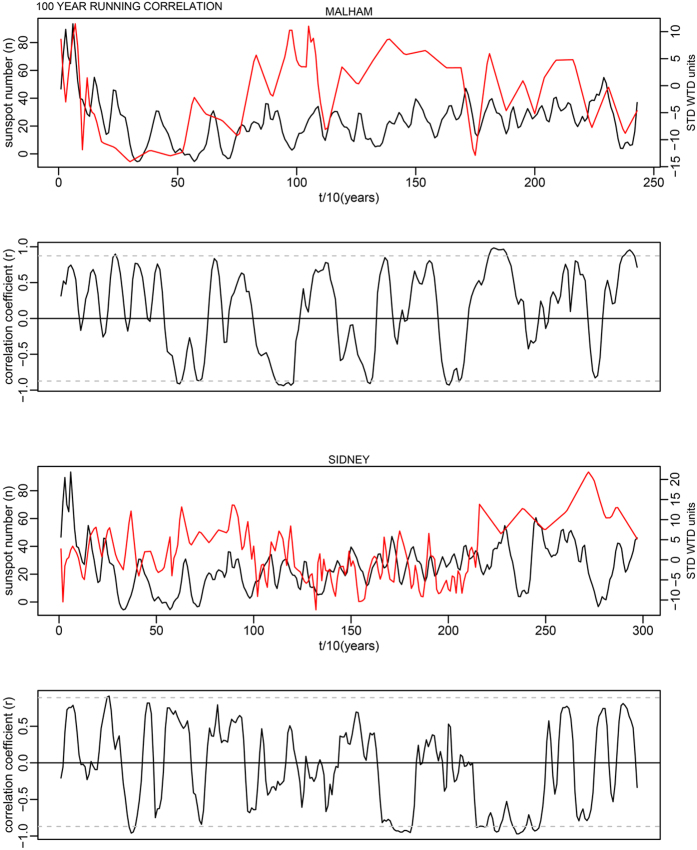


**Figure 5:**
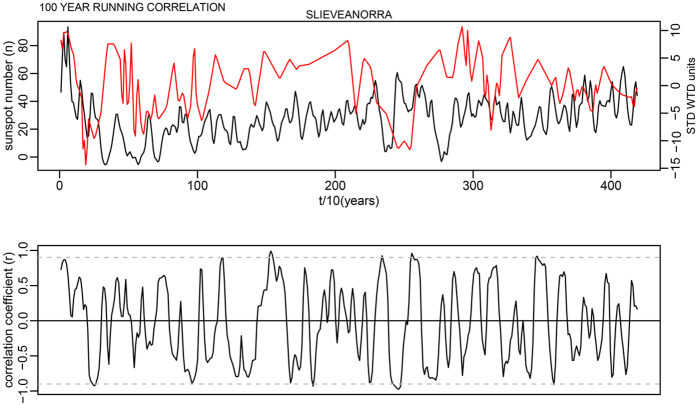


**Figure 6:**
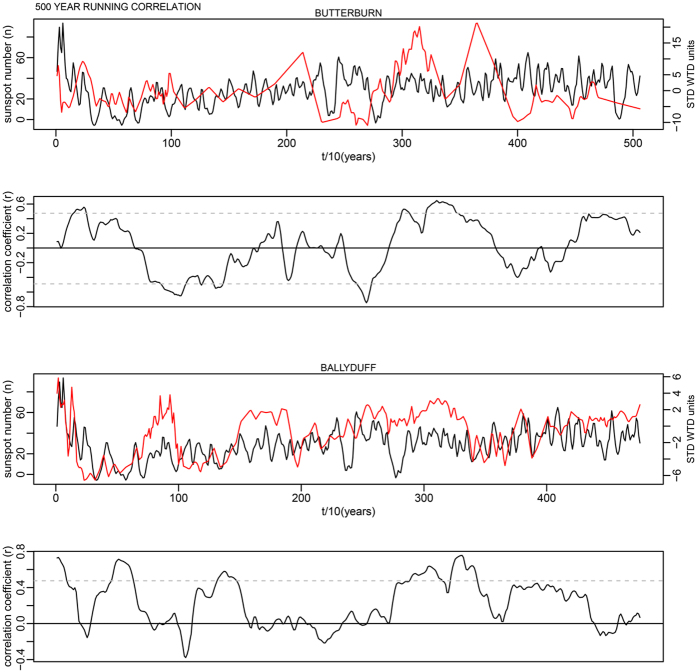


**Figure 7:**
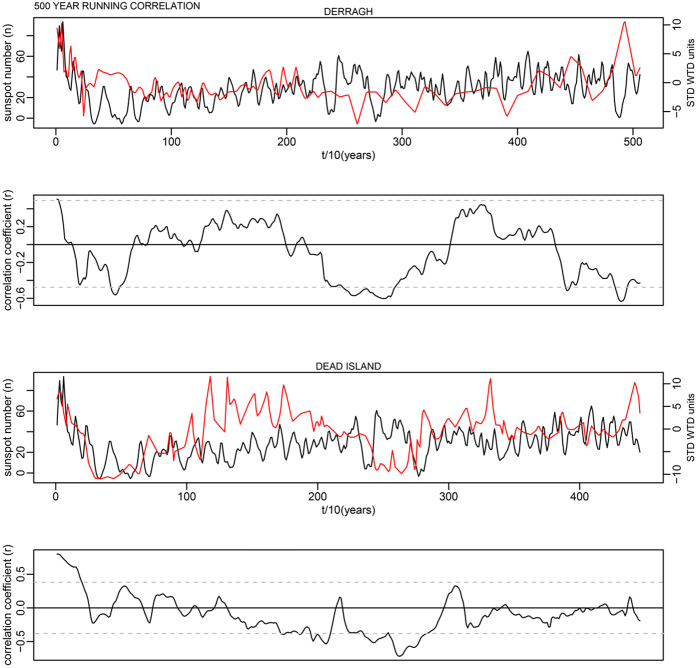


**Figure 8:**
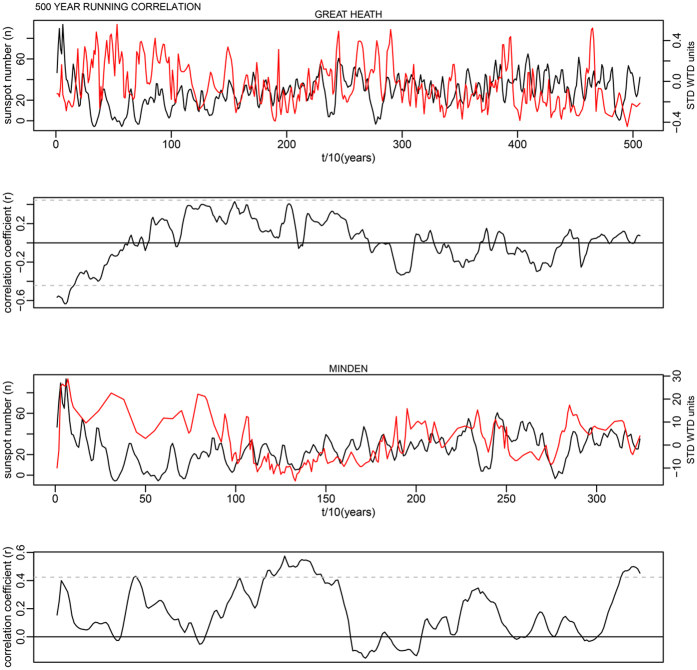


**Figure 9:**
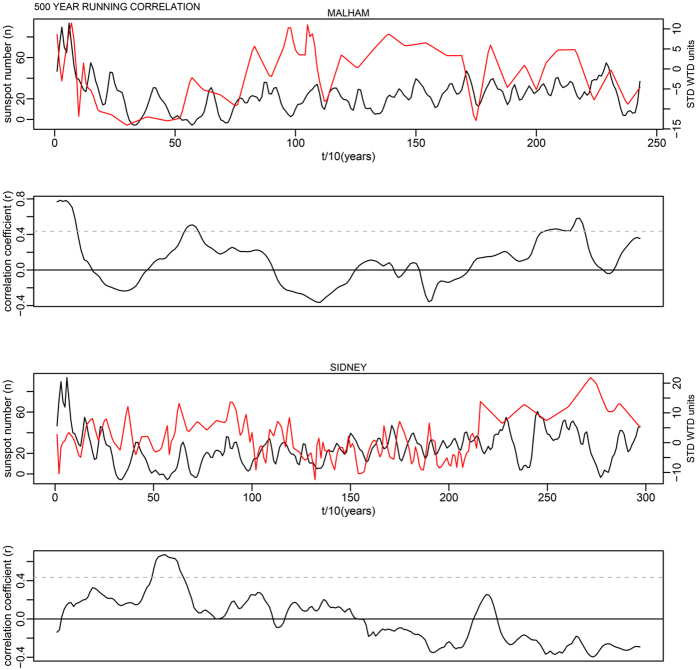


**Figure 10:**
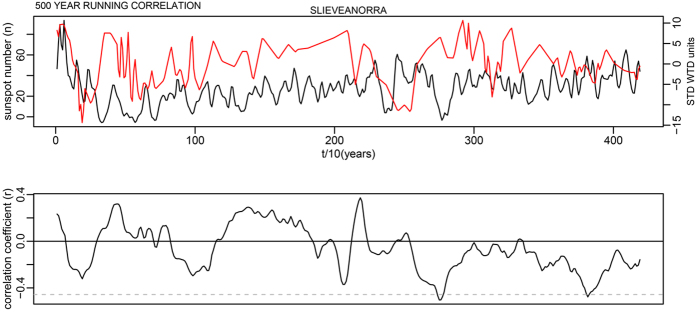


**Table 1:** 

Site code	Original critical level (100 year)	Corrected critical level (100 year)	Original critical level (500 year)	Corrected critical level (500 year)
BB	0.98	0.90	0.65	0.44
BD	0.98	0.90	0.75	0.44
DE	0.97	0.90	0.51	0.44
DI	0.96	0.90	0.80	0.43
GH	0.95	0.90	0.43	0.44
MI	0.98	0.89	0.57	0.41
MT	0.98	0.88	0.78	0.41
SI	0.91	0.89	0.67	0.42
SL	0.99	0.89	0.37	0.43

