# Supplementary material for: Solar cycles or random processes? Evaluating solar variability in Holocene climate records
Source: Sci Rep. 2016 Apr 5;6:23961. doi: 10.1038/srep23961 (PMC4820721; doi:10.1038/srep23961)
Supplement: Supplementary Information [file srep23961-s1.pdf]

Solar cycles or random processes? Evaluating solar variability in Holocene climate records

Supplementary Information

T. Edward Turner

School of Geography, University of Leeds, LS2 9JT, UK

Graeme T. Swindles

School of Geography, University of Leeds, LS2 9JT, UK

Dan J. Charman

Department of Geography, University of Exeter, EX4 4RJ, UK

Peter G. Langdon

Geography and Environment, University of Southampton, Southampton, SO17 1BJ, UK

Paul J. Morris

School of Geography, University of Leeds, LS2 9JT, UK

Robert K. Booth

Earth & Environmental Sciences, Lehigh University, Bethlehem, PA18015-3001, USA

Lauren E. Parry

School of Interdisciplinary Studies, University of Glasgow, DG1 4ZL, UK

Jonathan E. Nichols

Lamont-Doherty Earth Observatory at Columbia University, Palisades, NY 10964, USA

1 Materials and methods & site map

Full Method

We examined nine high-resolution (defined as having a sampling frequency of 2 cm or less and >10 ^{14}C radiocarbon dates per metre and/or precise known-age stratigraphic markers) proxy climate records from ombrotrophic bogs located in the Northern Hemisphere (Europe and the USA) (Figure 1 and SM1 Figure shown below). Eight of these records are based on transfer function-reconstructions of water-table depth from testate amoebae subfossils (Turner et al., 2014, Clifford and Booth, 2013, Booth et al., 2012, Mauquoy et al., 2008, Swindles et al., 2013), and one is based on *Sphagnum*/Vascular Ratio derived from ratios of leaf wax compounds (Nichols and Huang, 2012). Bayesian age-depth models for the proxy records were generated from radiocarbon dates and age-equivalent stratigraphic markers (tephra and spheroidal carbonaceous particles) using the Bacon software package (Blaauw and Christen, 2011) in R v. 3.1.2 (R Core Team, 2014). We describe the age-depth models as having 'high quality age control' in the manuscript. We define this as: age-depth models with at least 10 radiocarbon dates, most of which are supplemented by known-age stratigraphic markers such as microtephra layers, pollen markers, or SCPs. In the case of Slieveanorra and Dead Island, these age-depth models have >10 known-age stratigraphic markers, supplemented by radiocarbon dates.

A series of 15 simple one-dimensional random walks in discreet time (RWs) (cf. Blaauw et al., 2010: Özel, 2015) per site were generated in R v.3.1.2 (R Core Team, 2014) based on the time period for each proxy dataset (e.g. Dead Island = 4454 years). Each random walk starts from an initial value of zero with subsequent values generated from a normal distribution (cumulative sums of standard normals) with the mean taken from the value at the previous step, generating one random value per step, or 'year'. The R code is given in the supplementary material (S8). The RWs were interpolated to 10 year time-steps to more closely resemble peatland dynamics (e.g. typical peat accumulation rate = 1 cm yr^{-1}). Data were then linearly detrended prior to linear interpolation to the same time-steps generated from the Bayesian age-depth model for each site, such that both RW and real data

for each site exhibit the same temporal framework. The sunspot reconstruction of Solanki et al. (2004) was used as the record of changing solar activity through the mid-late Holocene.

Data were linearly detrended prior to spectral analysis. REDFIT spectral analysis (Lomb-Scargle Fourier Transform method) using the PAST software (Hammer et al., 2001) was used to test for periodicities in the proxy data and random walks. REDFIT tests for a null hypothesis of red (autocorrelated) noise in the data by fitting the time series to an AR(1) red noise model. The significance of spectral peaks was tested using a parametric (χ^2) approach (90, 95 and 99 % false-alarm levels). A runs test was used to test the appropriateness red noise model. The red noise model was deemed an appropriate description of all the proxy climate data (within 5 % acceptance interval). A white-noise background was also used for the sunspot data which contains strong periodic components and failed the runs test. In this case, Harmonic analysis was used to detect periodic signal components in the presence of noise (Borgmark, 2005, Percival and Walden, 1993). A rectangular window was used with the significance set to $\alpha = 0.05$ and $\lambda = 0.4$ (99.6 % false alarm level) for Siegel's test (Siegel, 1980) with two or three harmonic components. The analysis was carried out using the software package SPECTRUM (Schulz and Stattegger, 1997).

The proxy data and RWs were interpolated to a 10-year time interval for continuous wavelet analyses. Continuous wavelet transforms (CWT) (Morlet mother wavelet) were used to determine non-stationary periodicities. Cross-wavelet (XWT) analysis was used to explore common features in wavelet power-of-two time series (Maraun and Kurths, 2004). XWT highlights regions when there is a high common power between two time series (Grinsted et al., 2004).

Bivariate correlation analysis was carried out to determine if there were general correlations between the proxy data and solar variability, and random walk data and solar variability. Running correlation analysis was used to determine the temporal variation of the correlation (time windows = 100 years and 500 years). The statistical significance of the correlation was calculated using a Monte Carlo simulation to determine the null distribution. We calculated a running correlation for

many different random variables and found the highest correlation for each trial. We used the same number of data point and window width as the dataset to determine the critical value (see R code in supplementary material S8). The analyses were carried out using the gtools (Warnes et al., 2014) and biwavelet libraries (Gouhier, 2014) in R (R Core Team, 2014). We carried out the running correlations with both 100 and 500-year bins to examine whether the lack of correlation was related to interpolation.

An additional 5000 RWs were generated and interpolated to 10 year time-steps only (i.e. not further interpolated to 'real' age-depth model time-steps). These 5000 RWs were tested for significant positive correlation (Spearman's Rank, $p < 0.05$) with the solar reconstruction of Solanki et al. (2004).

The CO_2 record in Figure 1 is combined from Mauna Loa, the Law Dome and EPICA Dome C ice cores (Keeling et al., 1976, Etheridge et al., 1996, Monnin et al., 2004)

References

BLAAUW, M., BENNETT, K. D. & CHRISTEN, J. A. 2010. Random walk simulations of fossil proxy data. The Holocene, 20, 645-649.

BLAAUW, M. & CHRISTEN, J. A. 2011. Flexible paleoclimate age-depth models using an autoregressive gamma process. *Bayesian Analysis,* 6**,** 457-474.

BOOTH, R. K., JACKSON, S. T., SOUSA, V. A., SULLIVAN, M. E., MINCKLEY, T. A. & CLIFFORD, M. J. 2012. Multi-decadal drought and amplified moisture variability drove rapid forest community change in a humid region. *Ecology,* 93**,** 219-226.

BORGMARK, A. 2005. Holocene climate variability and periodicities in south-central Sweden, as interpreted from peat humification analysis. *The Holocene,* 15**,** 387-395.

CLIFFORD, M. & BOOTH, R. 2013. Increased probability of fire during late Holocene droughts in northern New England. *Climatic Change,* 119**,** 693-704.

ETHERIDGE, D. M., STEELE, L. P., LANGENFELDS, R. L., FRANCEY, R. J., BARNOLA, J. M. & MORGAN, V. I. 1996. Natural and anthropogenic changes in atmospheric CO2 over the last 1000 years from air in Antarctic ice and firn. *Journal of Geophysical Research: Atmospheres,* 101**,** 4115-4128.

GOUHIER, T. 2014. biwavelet: Conduct univariate and bivariate wavelet analyses (Version 0.14).

GRINSTED, A., MOORE, J. C. & JEVREJEVA, S. 2004. Application of the cross wavelet transform and wavelet coherence to geophysical time series. *Nonlinear Processes in Geophysics,* 11**,** 561-566.

HAMMER, Ø., HARPER, D. A. T. & RYAN, A. P. D. 2001. PAST: Paleontological Statistics Software Package for Education and Data Analysis. *Palaeontologia Electronica* 4**,** 9.

KEELING, C. D., BACASTOW, R. B., BAINBRIDGE, A. E., EKDAHL, C. A., GUENTHER, P. R., WATERMAN, L. S. & CHIN, J. F. S. 1976. Atmospheric carbon dioxide variations at Mauna Loa Observatory, Hawaii. *Tellus,* 28**,** 538-551.

MARAUN, D. & KURTHS, J. 2004. Cross wavelet analysis: significance testing and pitfalls. *Nonlinear Processes in Geophysics,* 11**,** 505-514.

MAUQUOY, D., YELOFF, D., VAN GEEL, B., CHARMAN, D. J. & BLUNDELL, A. 2008. Two decadally resolved records from north-west European peat bogs show rapid climate changes associated with solar variability during the mid–late Holocene. *Journal of Quaternary Science,* 23**,** 745-763.

MONNIN, E., STEIG, E. J., SIEGENTHALER, U., KAWAMURA, K., SCHWANDER, J., STAUFFER, B., STOCKER, T. F., MORSE, D. L., BARNOLA, J.-M., BELLIER, B., RAYNAUD, D. & FISCHER, H. 2004. Evidence for substantial accumulation rate variability in Antarctica during the Holocene, through synchronization of CO2 in the Taylor Dome, Dome C and DML ice cores. *Earth and Planetary Science Letters,* 224**,** 45-54.

NICHOLS, J. E. & HUANG, Y. 2012. Hydroclimate of the northeastern United States is highly sensitive to solar forcing. *Geophysical Research Letters,* 39**,** L04707.

ÖZEL, G. 2015. in *Handbook of Research on Behavioral Finance and Investment Strategies* (ed. Copur, Z.) Ch. 11, 191-193. Hershey PA, USA, IGI Global.

PERCIVAL, D. B. & WALDEN, A. T. 1993. *Spectral Analysis for Physical Applications: Multitaper and Conventional Univariate Techniques,* Cambridge, Cambridge University Press.

R CORE TEAM 2014. R: A language and environment for statistical computing. Vienna, Austria: R Foundation for Statistical Computing.

SCHULZ, M. & STATTEGGER, K. 1997. Spectrum: spectral analysis of unevenly spaced paleoclimatic time series. *Comput. Geosci.,* 23**,** 929-945.

SIEGEL, A. F. 1980. Testing for periodicity in a time series. *J. Am. Statist. Assoc. ,* 75**,** 345-348.

SOLANKI, S. K., USOSKIN, I. G., KROMER, B., SCHUSSLER, M. & BEER, J. 2004. Unusual activity of the Sun during recent decades compared to the previous 11,000 years. *Nature* 431, 1084-1087.

SWINDLES, G. T., LAWSON, I. T., MATTHEWS, I. P., BLAAUW, M., DALEY, T. J., CHARMAN, D. J., ROLAND, T. P., PLUNKETT, G., SCHETTLER, G., GEAREY, B. R., TURNER, T. E., REA, H. A., ROE, H. M., AMESBURY, M. J., CHAMBERS, F. M., HOLMES, J., MITCHELL, F. J. G., BLACKFORD, J., BLUNDELL, A., BRANCH, N., HOLMES, J., LANGDON, P., MCCARROLL, J., MCDERMOTT, F., OKSANEN, P. O., PRITCHARD, O., STASTNEY, P., STEFANINI, B., YOUNG, D., WHEELER, J., BECKER, K. & ARMIT, I. 2013. Centennial-scale climate change in Ireland during the Holocene. *Earth-Science Reviews,* 126**,** 300-320.

TURNER, T. E., SWINDLES, G. T. & ROUCOUX, K. H. 2014. Late Holocene ecohydrological and carbon dynamics of a UK raised bog: impact of human activity and climate change. *Quaternary Science Reviews,* 84**,** 65-85.

WARNES, G. R., BOLKER, B. & LUMLEY, T. 2014. gtools: Various R programming tools. R package version 3.4.1.

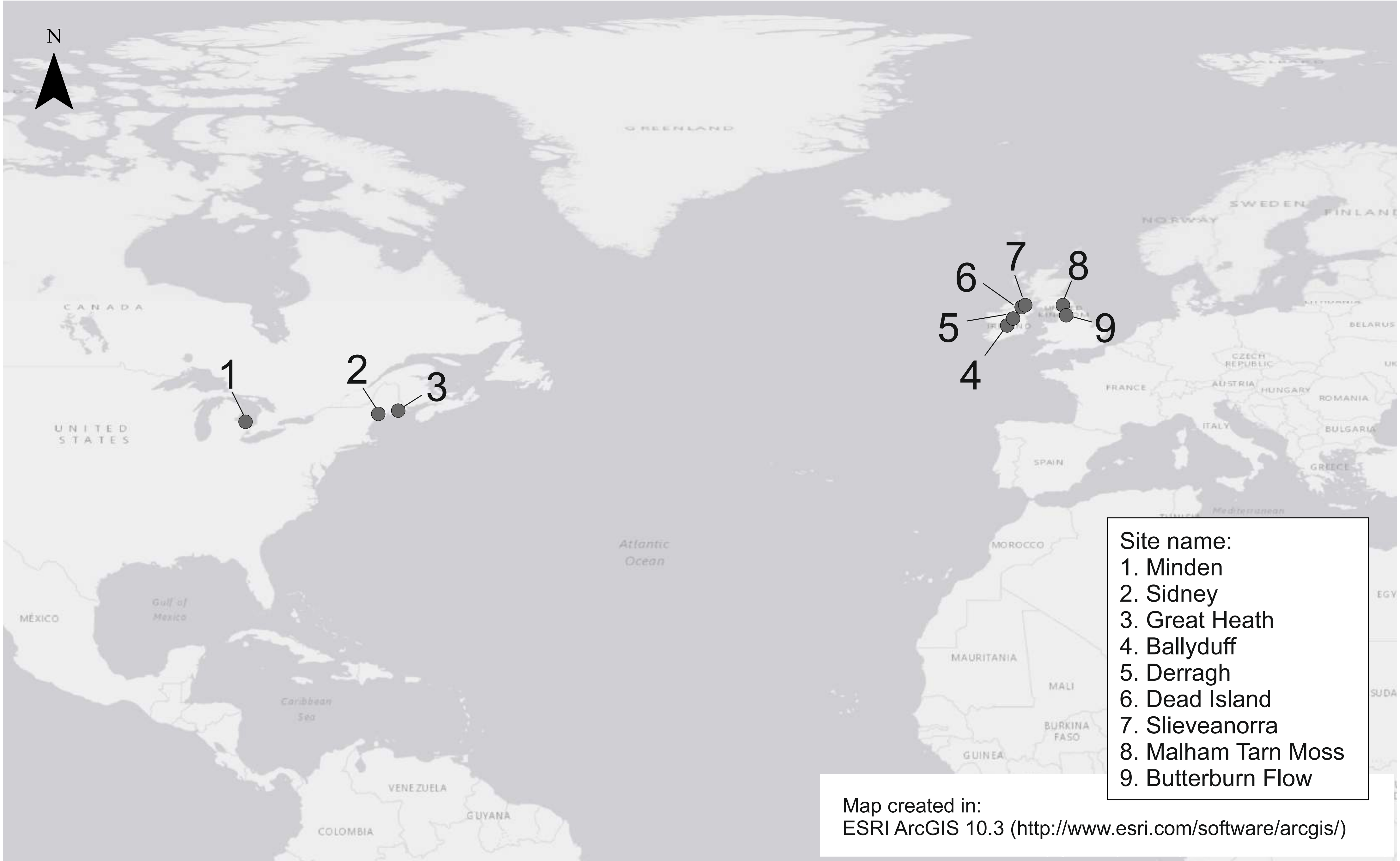
N
GREENLAND
CANADA
UNITED STATES
MÉXICO
Gulf of Mexico
Caribbean Sea
VENEZUELA
GUYANA
COLOMBIA
Atlantic Ocean
SWEDEN
NORWAY
FINLAND
BELARUS
CZECH REPUBLIC
AUSTRIA
HUNGARY
ROMANIA
BULGARIA
FRANCE
ITALY
SPAIN
GREECE
Mediterranean
MOROCCO
MAURITANIA
MALI
BURKINA FASO
GUINEA
1
2
3
4
5
6
7
8
9
Site name:
1. Minden
2. Sidney
3. Great Heath
4. Ballyduff
5. Derragh
6. Dead Island
7. Slieveanorra
8. Malham Tarn Moss
9. Butterburn Flow
Map created in:
ESRI ArcGIS 10.3 (http://www.esri.com/software/arcgis/)

2 Age-depth models

Bayesian age-depth models for all nine sites in this study. Major plot shows the age distributions, with darker greys indicating more probable calendar ages and the red curve showing the best model based on the weighted mean age for each depth. Calibrated ^{14}C dates are shown in blue, and age-equivalent time markers in pale green. The upper left plot shows the stability of the Markov Chain Monte Carlo iterations, and the upper middle and upper right plots show the prior (green curves) and posterior (grey histograms) distribution for accumulation rate and memory, respectively.

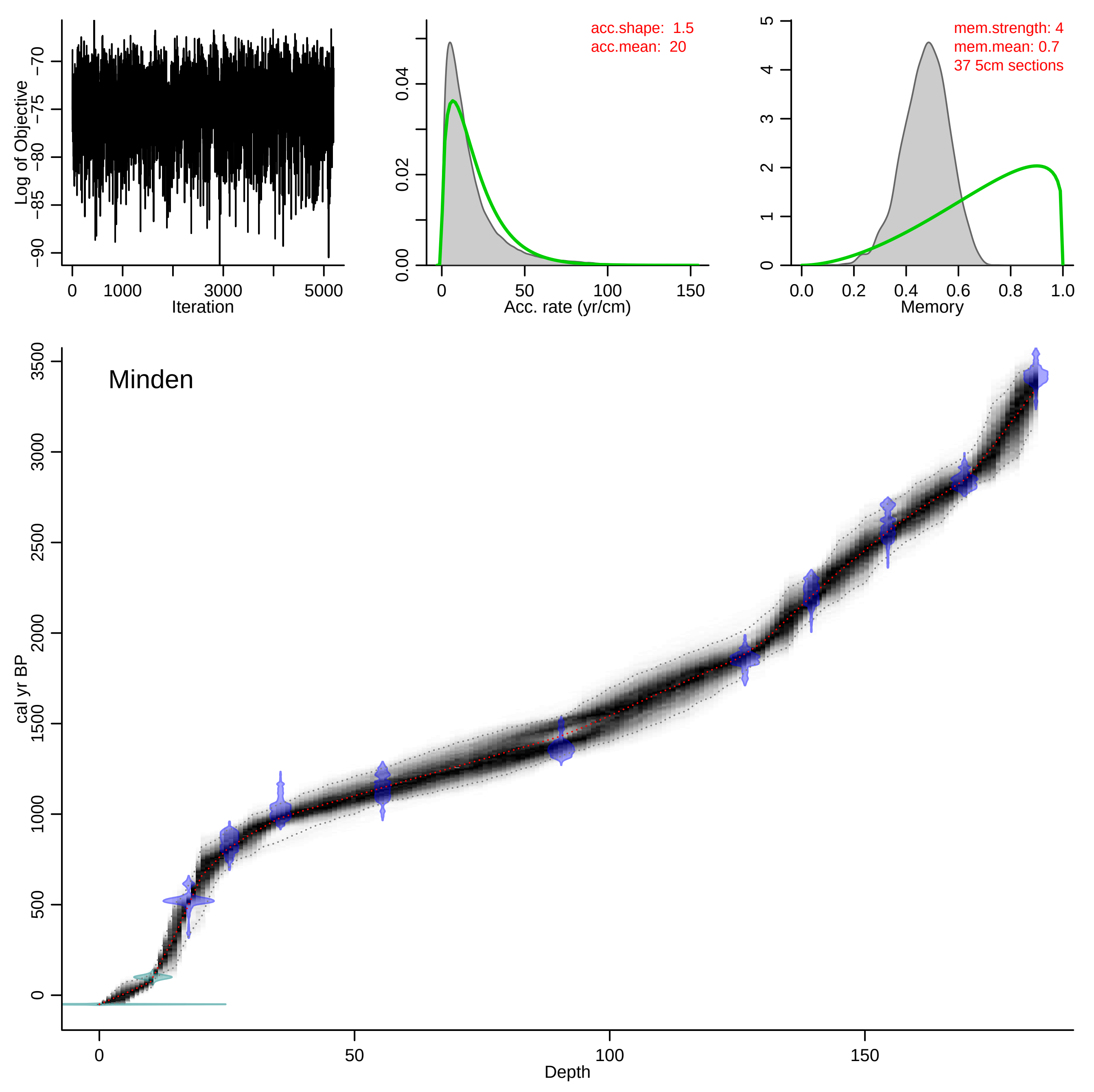

Log of Objective
Iteration
acc.shape: 1.5
acc.mean: 20
Acc. rate (yr/cm)
mem.strength: 4
mem.mean: 0.7
37 5cm sections
Memory
Minden
cal yr BP
Depth

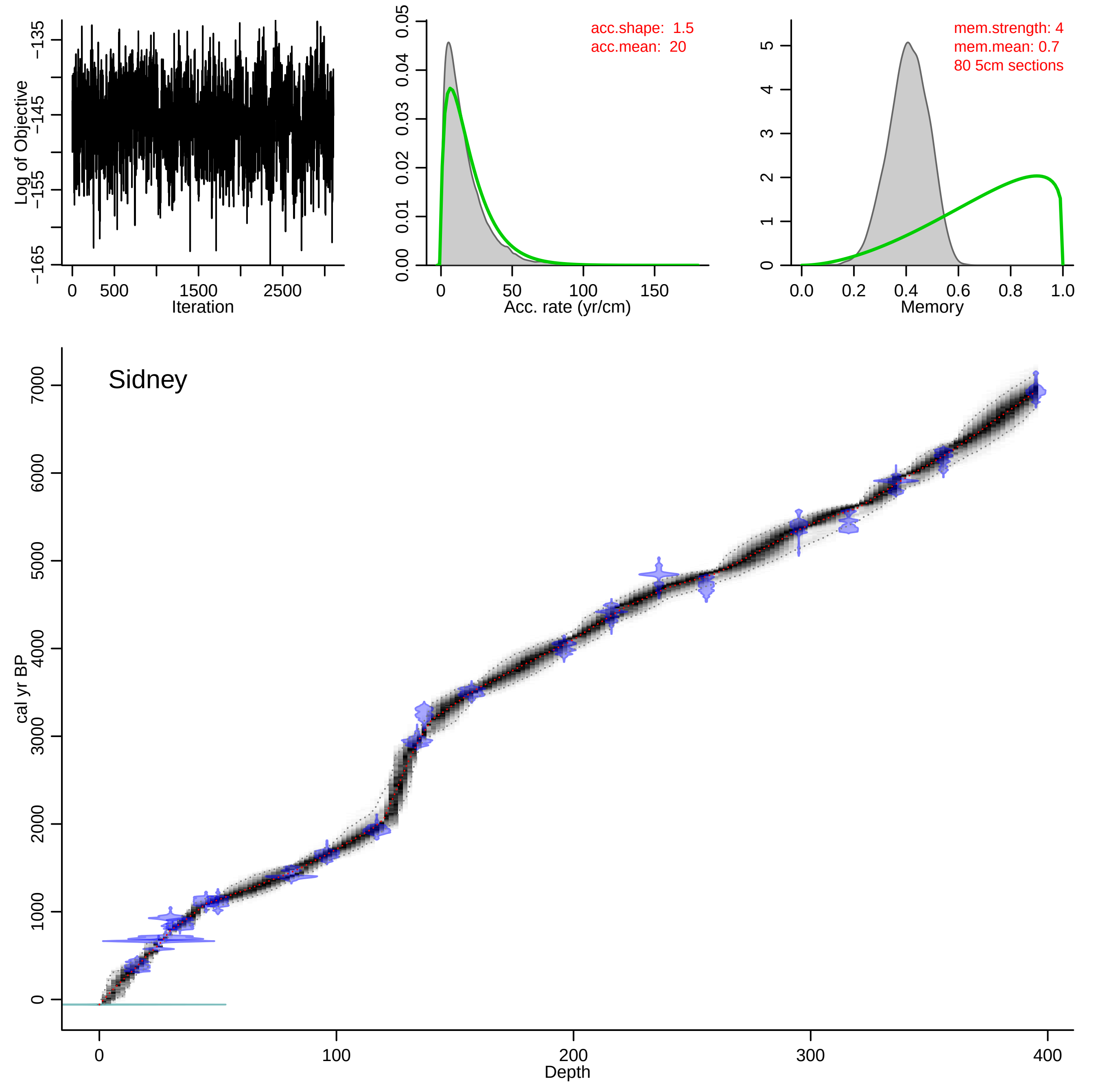

Log of Objective
−135
−145
−155
−165
0
500
1500
2500
Iteration
0.00
0.01
0.02
0.03
0.04
0.05
0
50
100
150
Acc. rate (yr/cm)
acc.shape: 1.5
acc.mean: 20
0
1
2
3
4
5
0.0
0.2
0.4
0.6
0.8
1.0
Memory
mem.strength: 4
mem.mean: 0.7
80 5cm sections
Sidney
cal yr BP
0
1000
2000
3000
4000
5000
6000
7000
0
100
200
300
400
Depth

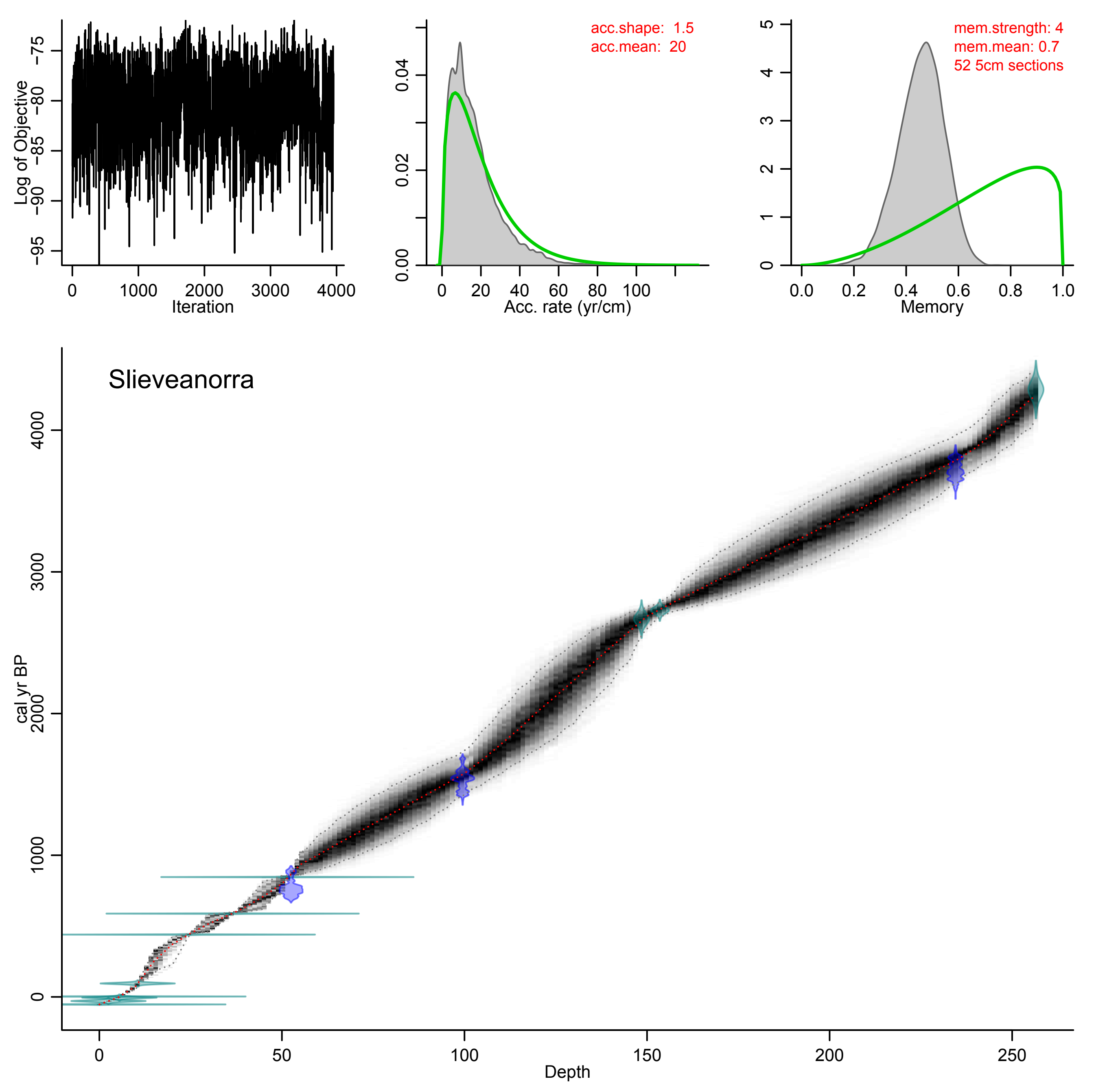
Log of Objective
Iteration
acc.shape: 1.5
acc.mean: 20
Acc. rate (yr/cm)
mem.strength: 4
mem.mean: 0.7
52 5cm sections
Memory
Slieveanorra
cal yr BP
Depth

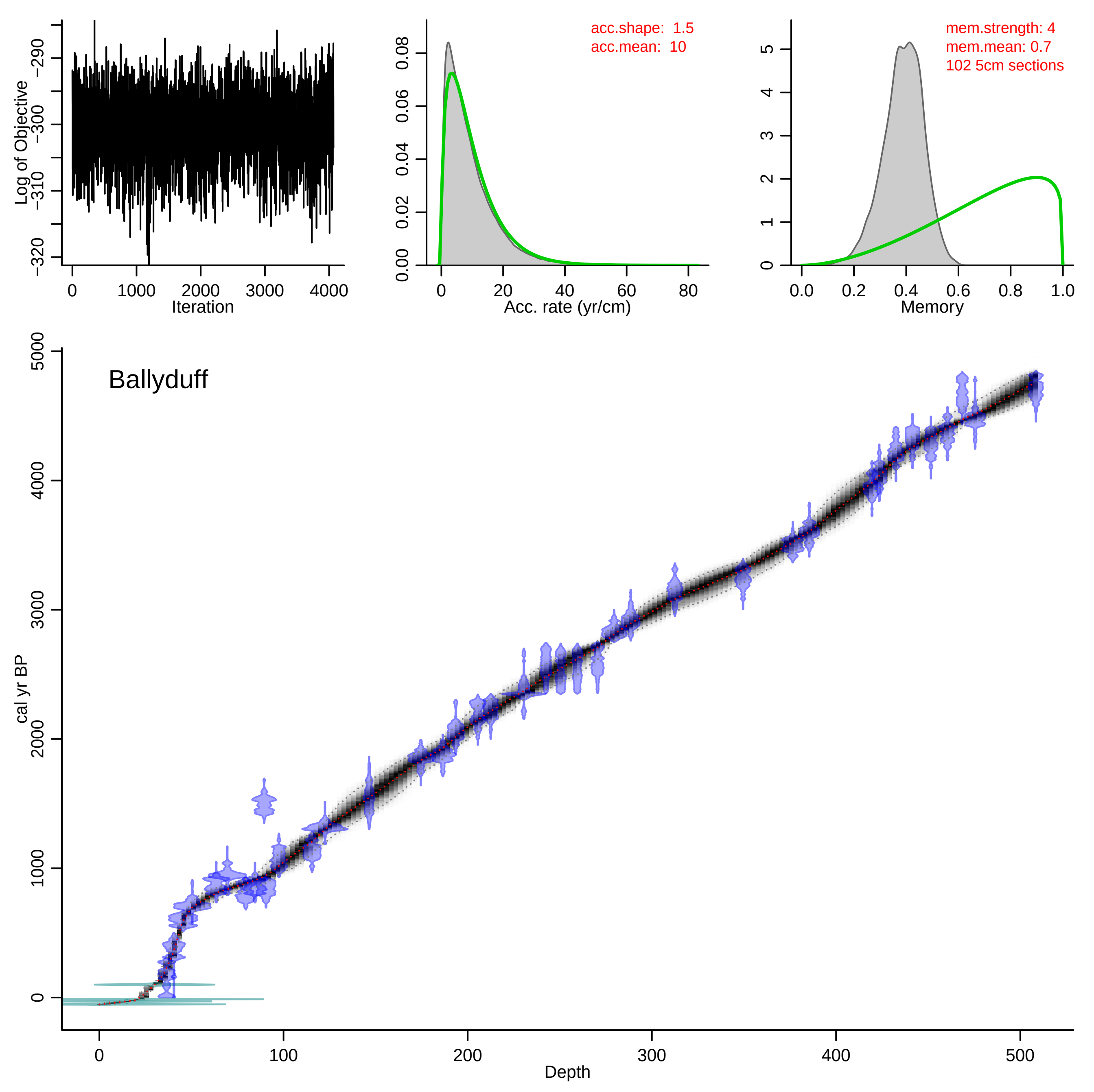

Log of Objective
Iteration
acc.shape: 1.5
acc.mean: 10
Acc. rate (yr/cm)
mem.strength: 4
mem.mean: 0.7
102 5cm sections
Memory
Ballyduff
cal yr BP
Depth

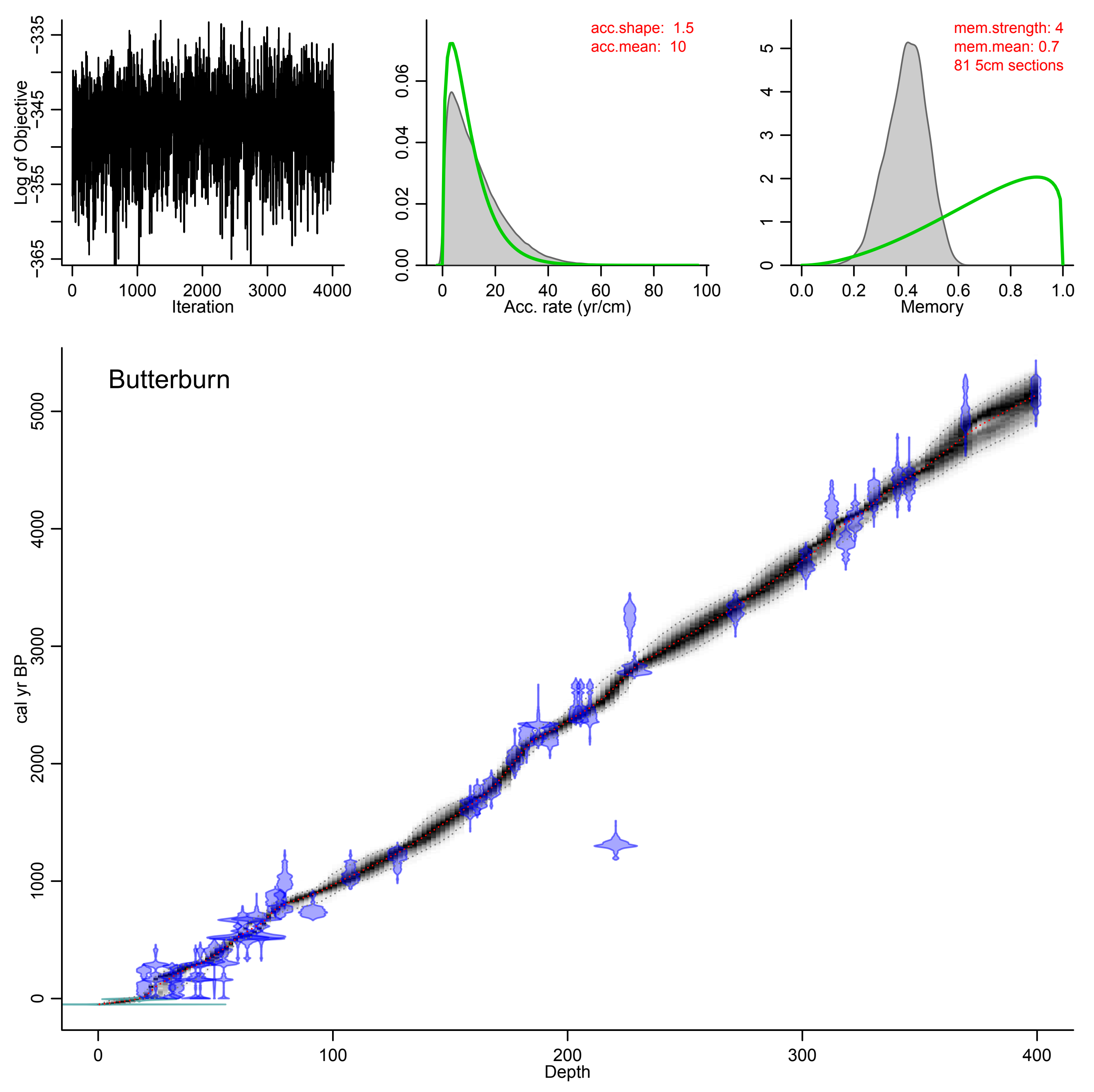

Log of Objective
Iteration
acc.shape: 1.5
acc.mean: 10
Acc. rate (yr/cm)
mem.strength: 4
mem.mean: 0.7
81 5cm sections
Memory
Butterburn
cal yr BP
Depth

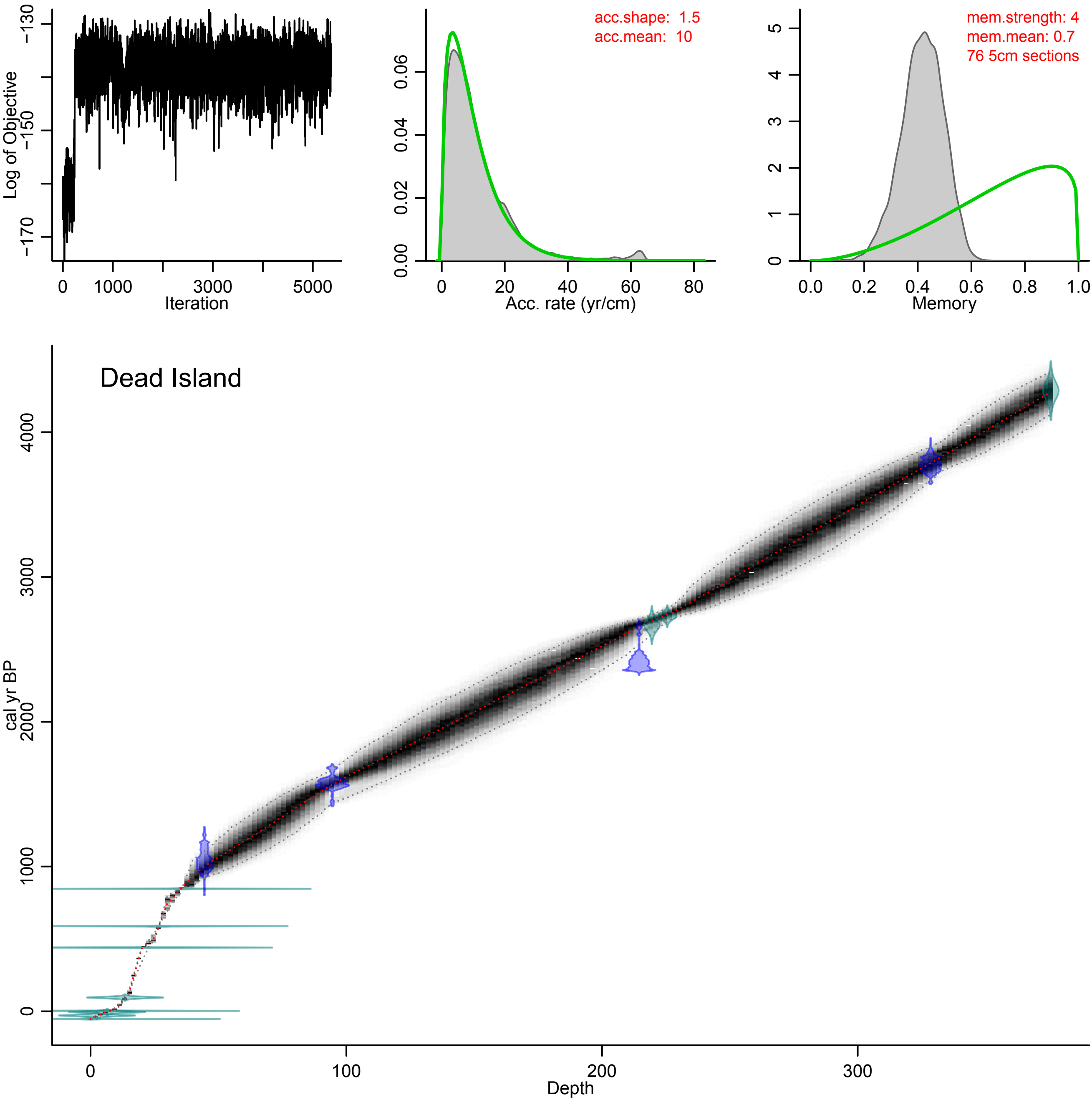
Log of Objective
Iteration
acc.shape: 1.5
acc.mean: 10
Acc. rate (yr/cm)
mem.strength: 4
mem.mean: 0.7
76 5cm sections
Memory
Dead Island
cal yr BP
Depth

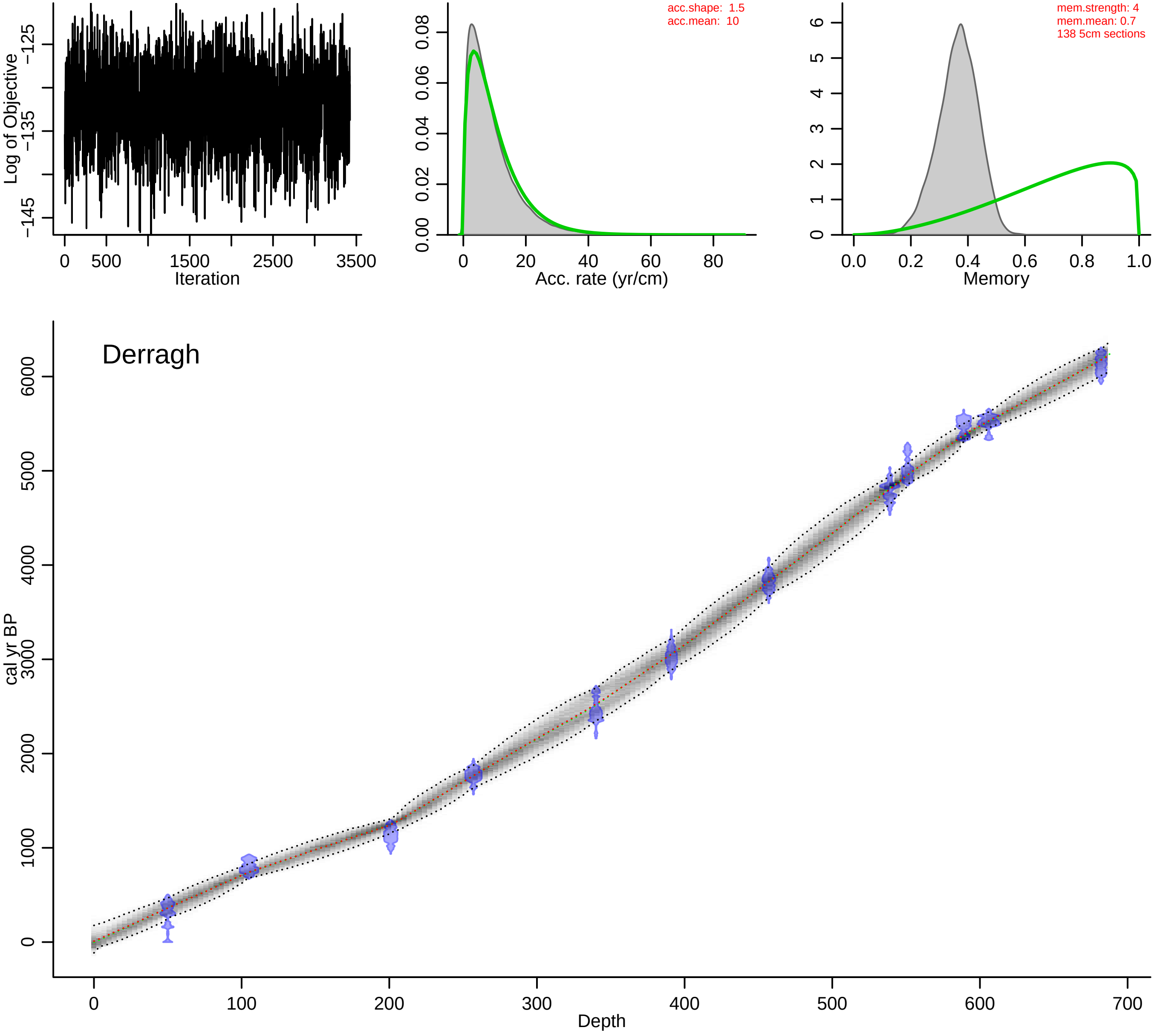

Log of Objective
Iteration
acc.shape: 1.5
acc.mean: 10
Acc. rate (yr/cm)
mem.strength: 4
mem.mean: 0.7
138 5cm sections
Memory
Derragh
cal yr BP
Depth

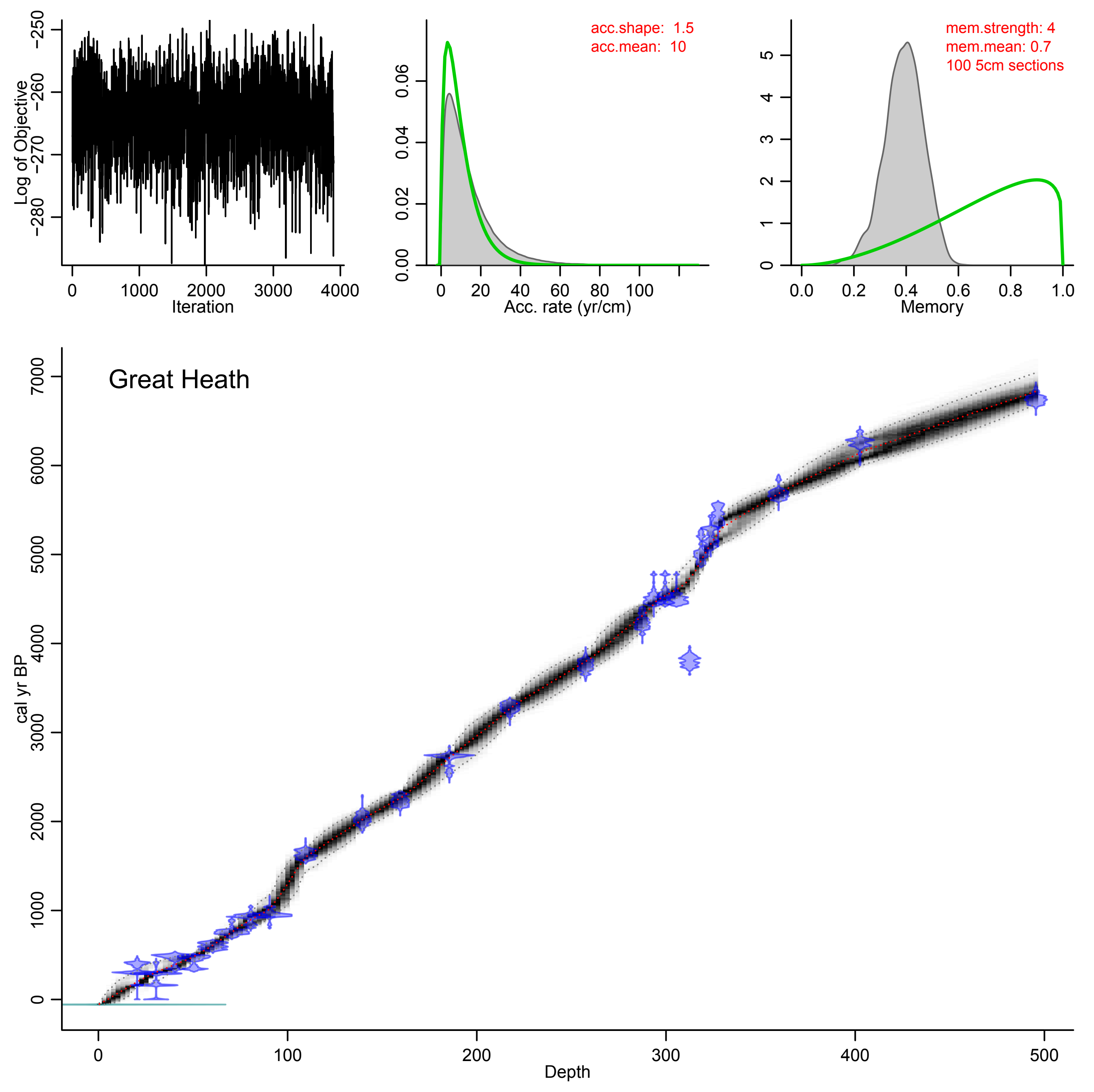
Log of Objective
Iteration
acc.shape: 1.5
acc.mean: 10
Acc. rate (yr/cm)
mem.strength: 4
mem.mean: 0.7
100 5cm sections
Memory
Great Heath
cal yr BP
Depth

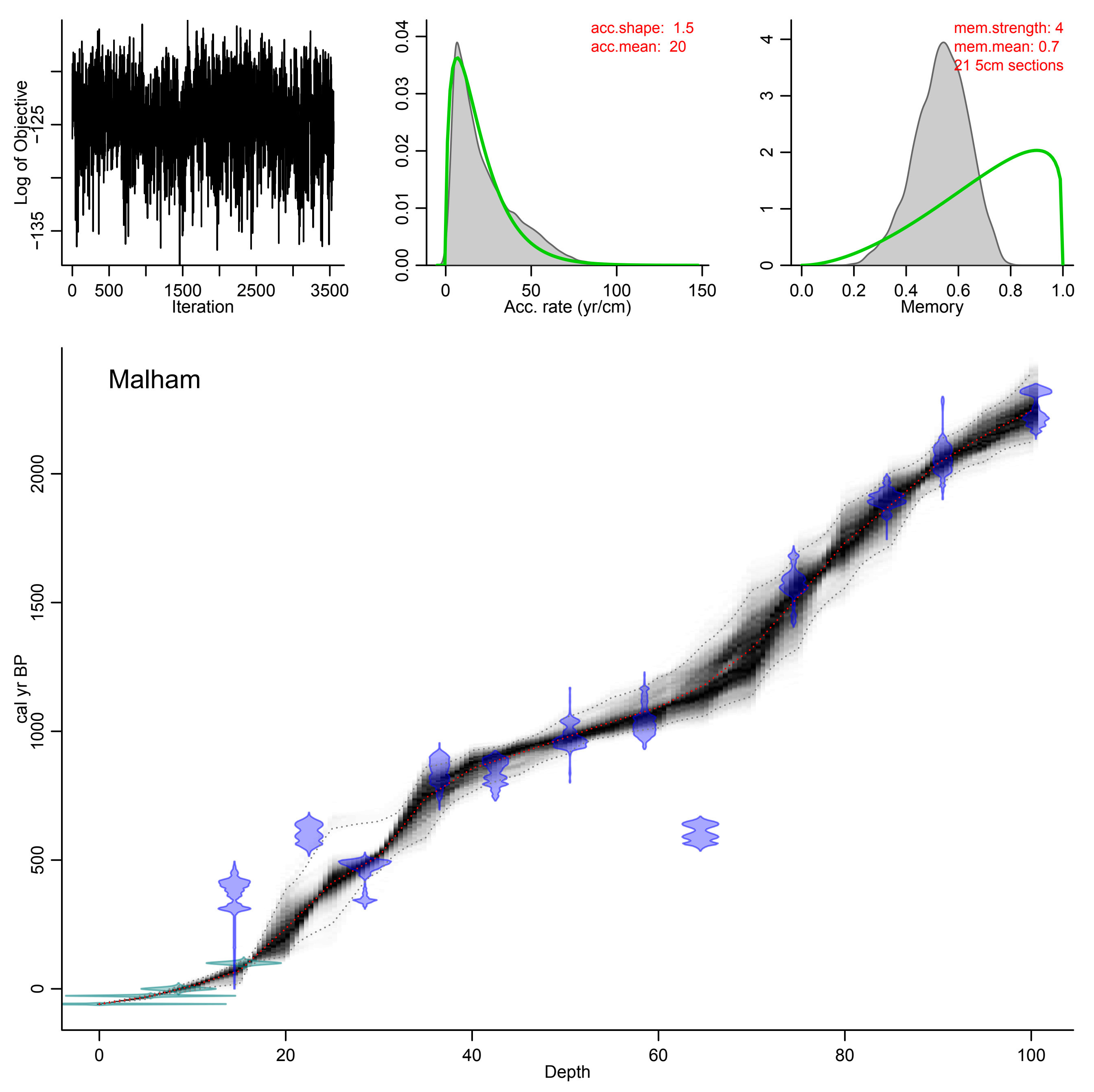

Log of Objective
Iteration
acc.shape: 1.5
acc.mean: 20
Acc. rate (yr/cm)
mem.strength: 4
mem.mean: 0.7
21 5cm sections
Memory
Malham
cal yr BP
Depth

3 Random walks

Plots show the 15 random walk (RW) iterations per site generated for this study. Each RW was detrended and interpolated to the corresponding proxy data age-depth model time-steps, shown on the x-axis (see full methods in Supplementary Material 1). Y-axis values have no units.

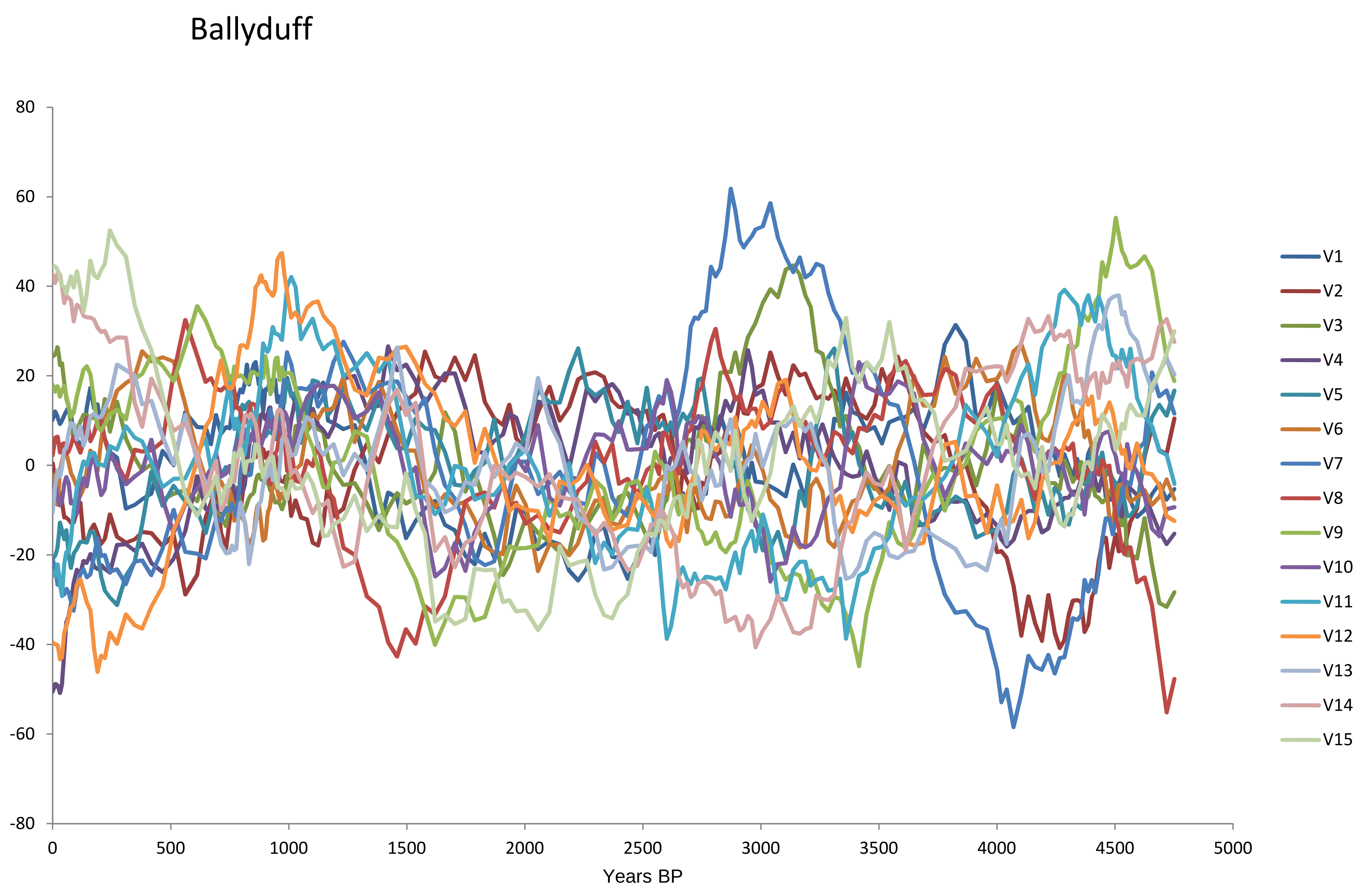

Ballyduff
80
60
40
20
0
-20
-40
-60
-80
0
500
1000
1500
2000
2500
3000
3500
4000
4500
5000
Years BP
V1
V2
V3
V4
V5
V6
V7
V8
V9
V10
V11
V12
V13
V14
V15

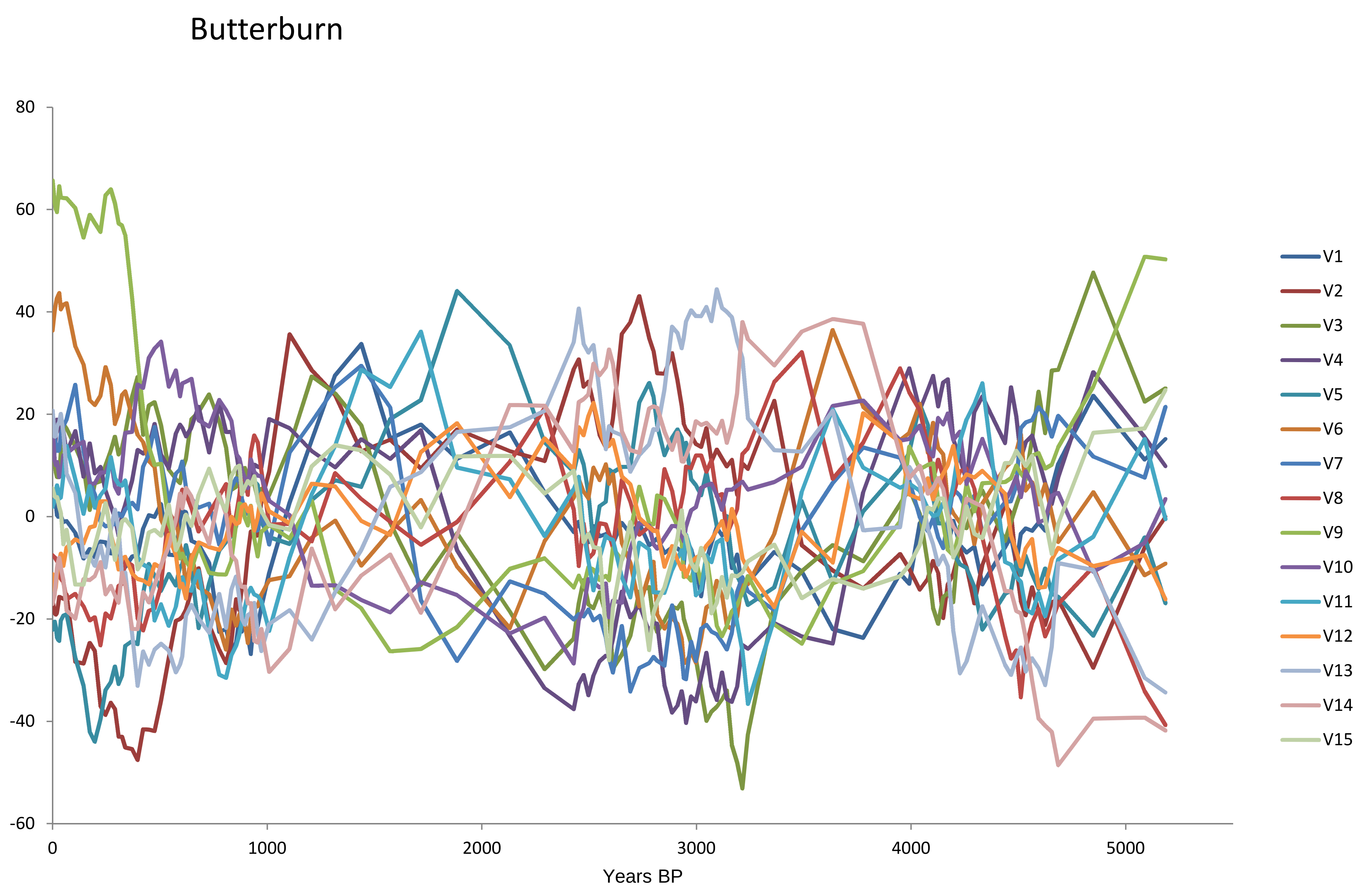
Butterburn
80
60
40
20
0
-20
-40
-60
0
1000
2000
3000
4000
5000
Years BP
V1
V2
V3
V4
V5
V6
V7
V8
V9
V10
V11
V12
V13
V14
V15

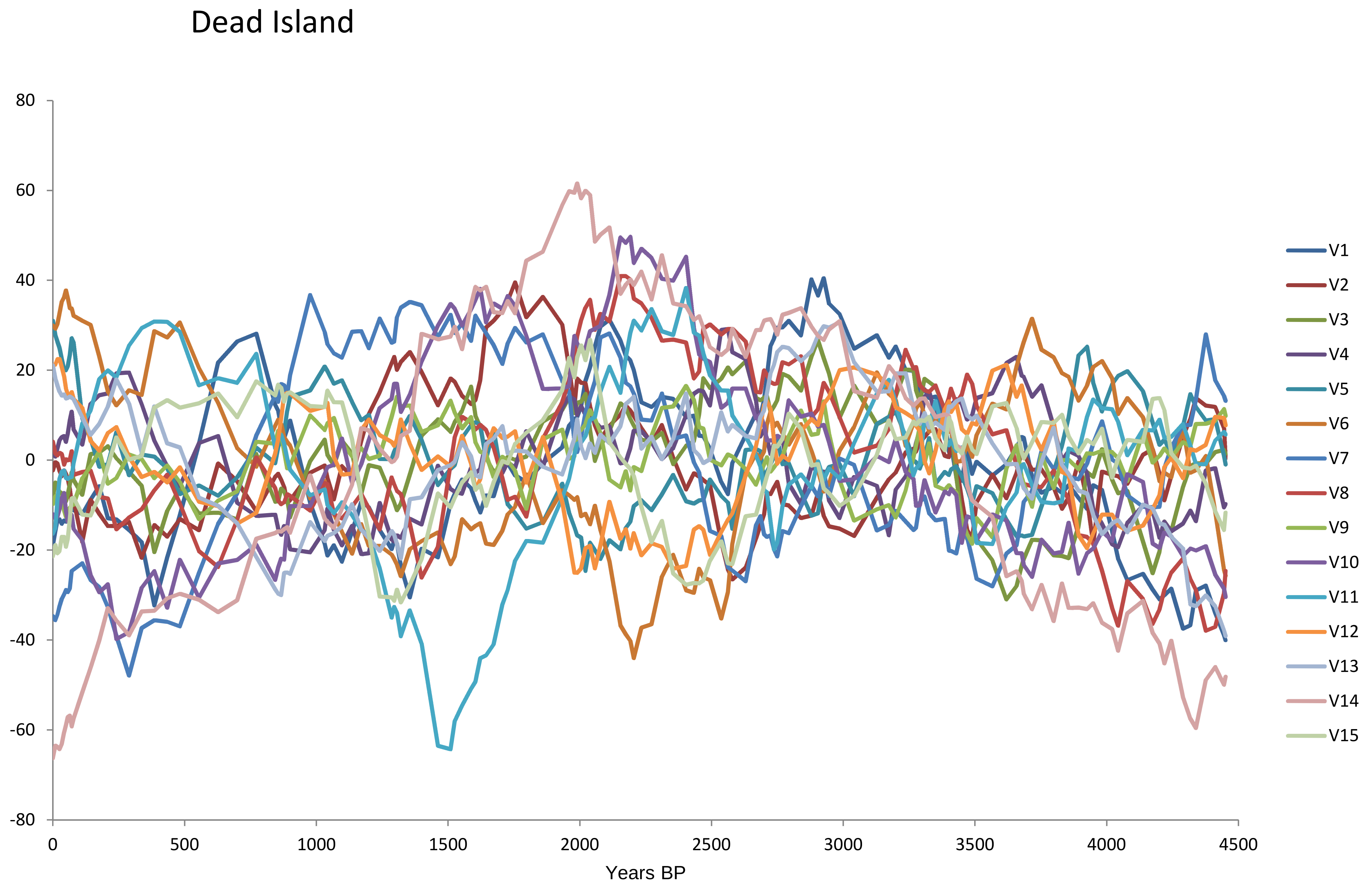

Dead Island
80
60
40
20
0
-20
-40
-60
-80
0
500
1000
1500
2000
2500
3000
3500
4000
4500
Years BP
V1
V2
V3
V4
V5
V6
V7
V8
V9
V10
V11
V12
V13
V14
V15

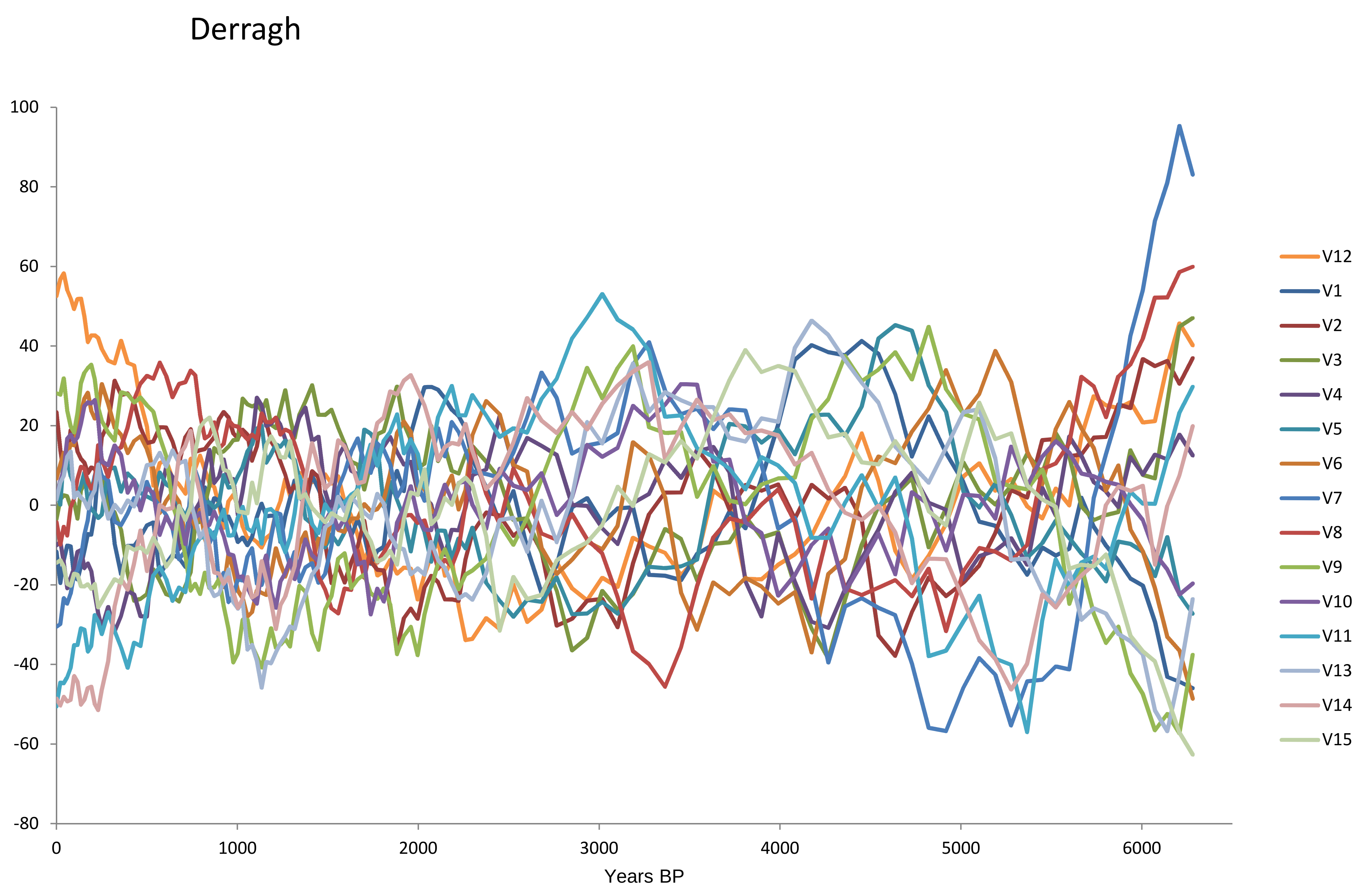
Derragh
100
80
60
40
20
0
-20
-40
-60
-80
0
1000
2000
3000
4000
5000
6000
Years BP
V12
V1
V2
V3
V4
V5
V6
V7
V8
V9
V10
V11
V13
V14
V15

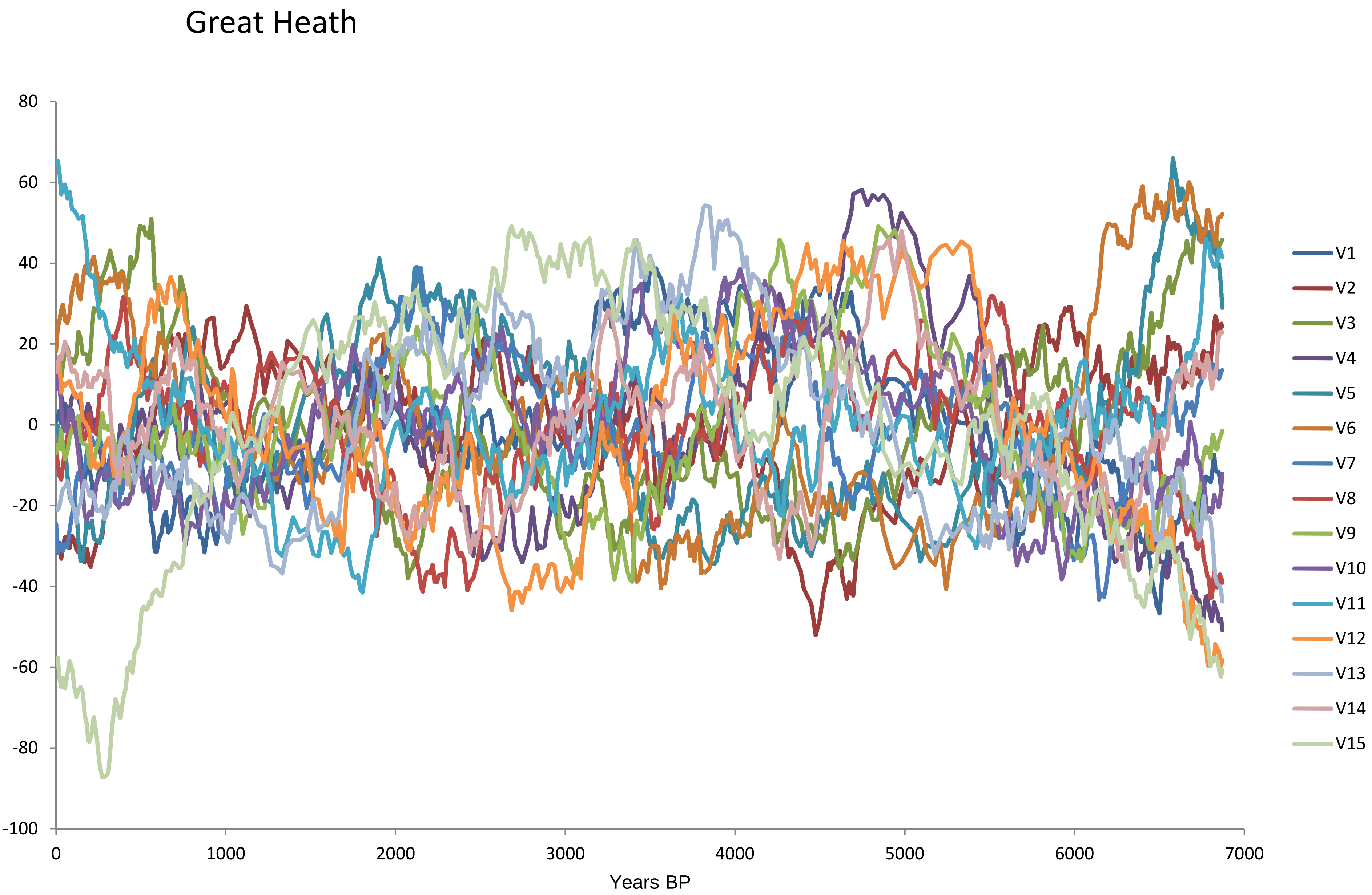
Great Heath
80
60
40
20
0
-20
-40
-60
-80
-100
0
1000
2000
3000
4000
5000
6000
7000
Years BP
V1
V2
V3
V4
V5
V6
V7
V8
V9
V10
V11
V12
V13
V14
V15

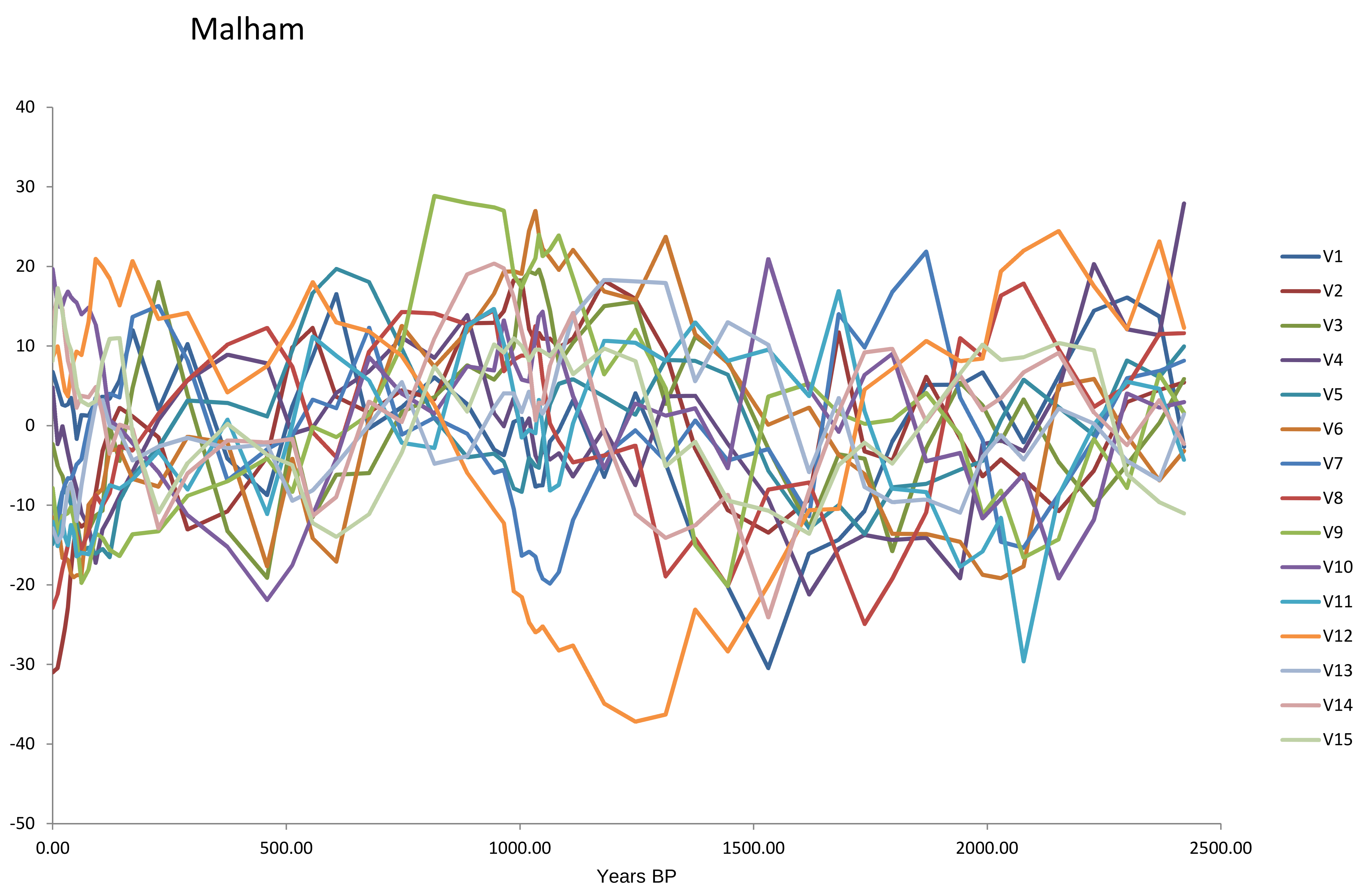
Malham
40
30
20
10
0
-10
-20
-30
-40
-50
0.00
500.00
1000.00
1500.00
2000.00
2500.00
Years BP
V1
V2
V3
V4
V5
V6
V7
V8
V9
V10
V11
V12
V13
V14
V15

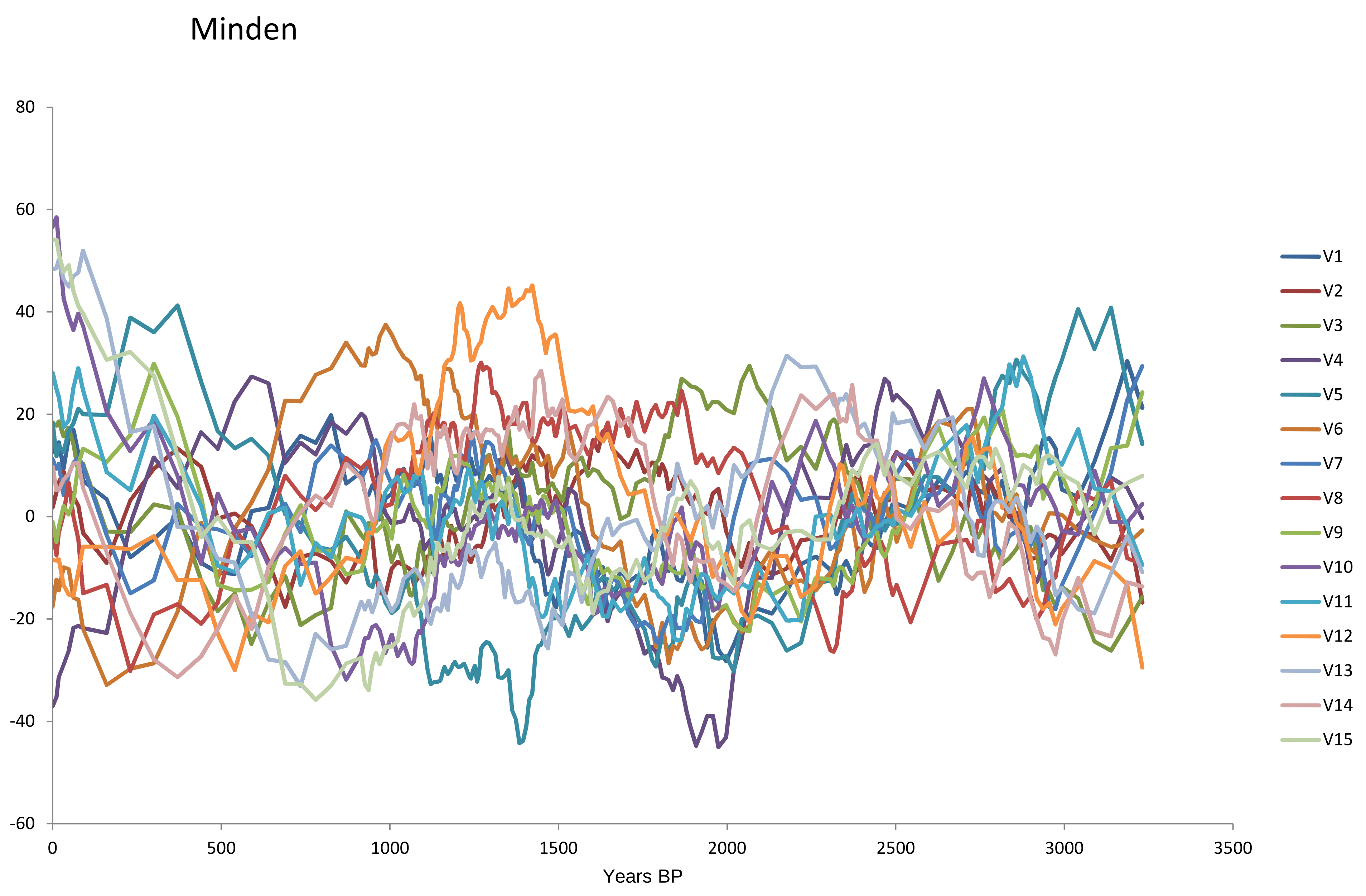
Minden
80
60
40
20
0
-20
-40
-60
0
500
1000
1500
2000
2500
3000
3500
Years BP
V1
V2
V3
V4
V5
V6
V7
V8
V9
V10
V11
V12
V13
V14
V15

Sidney

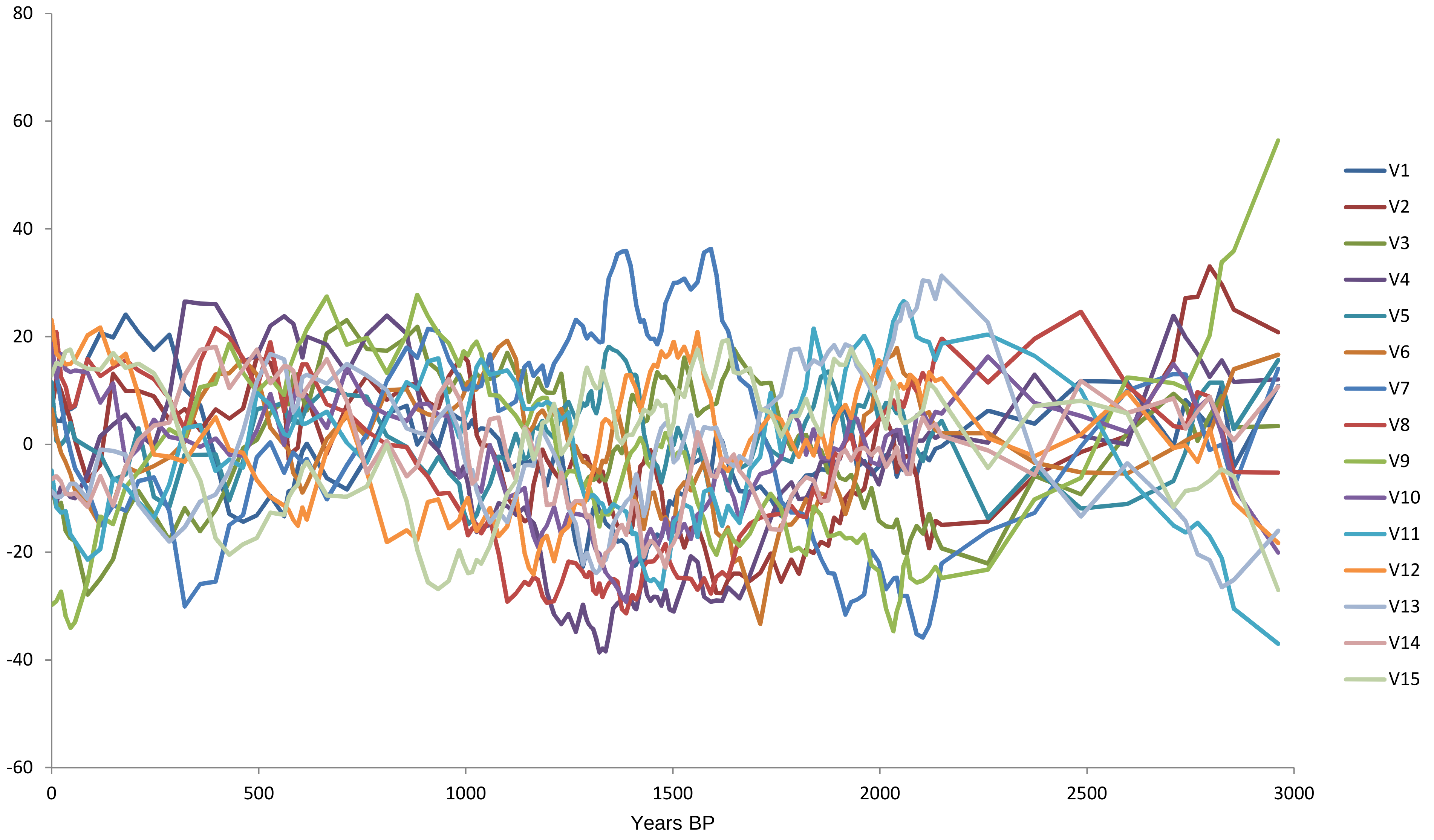

80
60
40
20
0
-20
-40
-60
0
500
1000
1500
2000
2500
3000
Years BP
V1
V2
V3
V4
V5
V6
V7
V8
V9
V10
V11
V12
V13
V14
V15

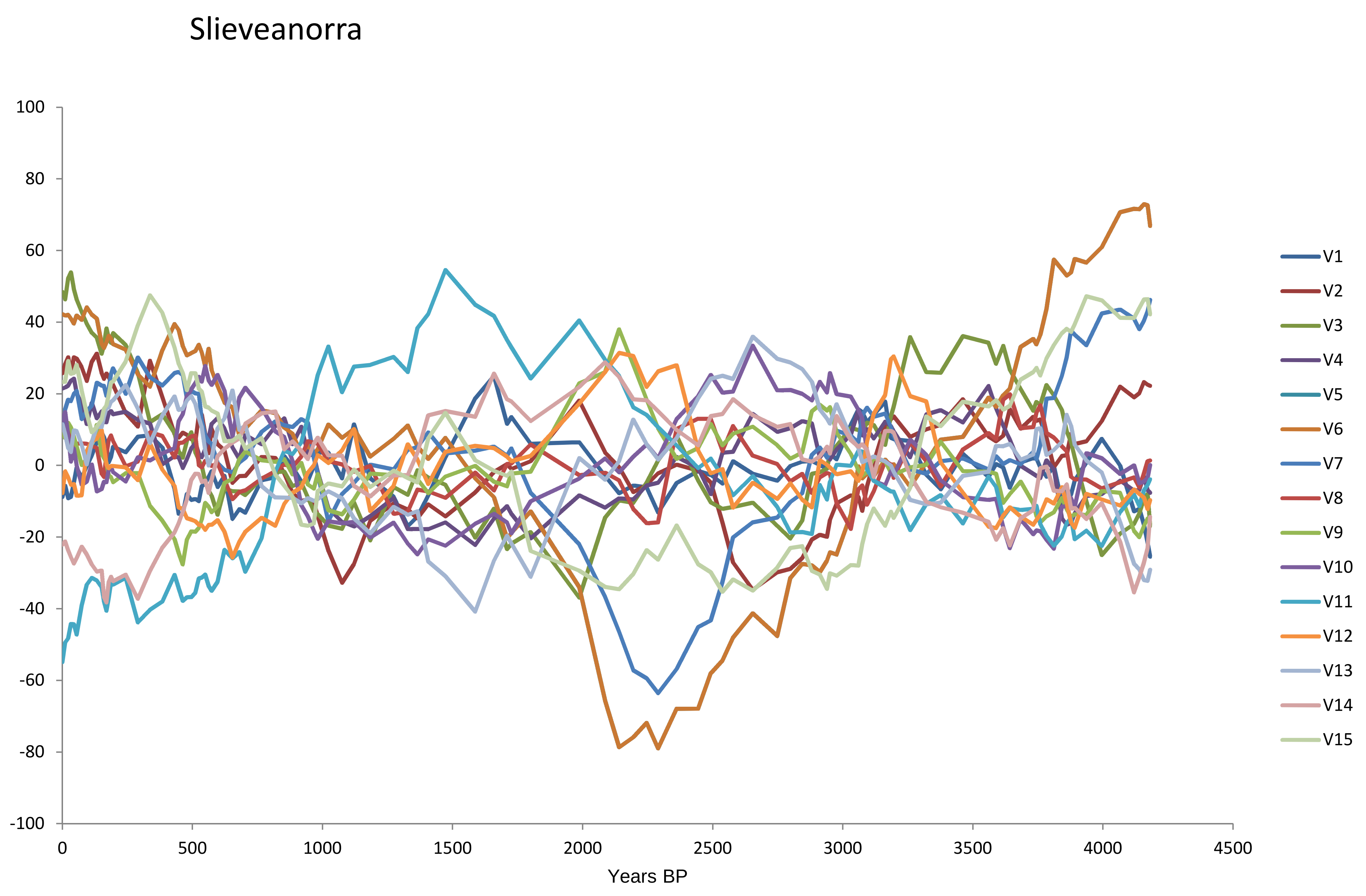

Slieveanorra
100
80
60
40
20
0
-20
-40
-60
-80
-100
0
500
1000
1500
2000
2500
3000
3500
4000
4500
Years BP
V1
V2
V3
V4
V5
V6
V7
V8
V9
V10
V11
V12
V13
V14
V15

4 Spectral analysis

Spectral (REDFIT) analysis of proxy-based water table reconstructions for the nine sites. Green lines indicate the 90, 95 and 99% false-alarm levels. Red lines show the fit to a theoretical AR(1) model. The bottom left and bottom middle panels show both the REDFIT and white noise spectral analysis of the Solar reconstruction of Solanki et al. (2004), respectively. Significant periodicities found in the white noise analysis are shown in the table linked by the arrow to the white noise plot. The critical level for Siegel’s test is shown (red line).

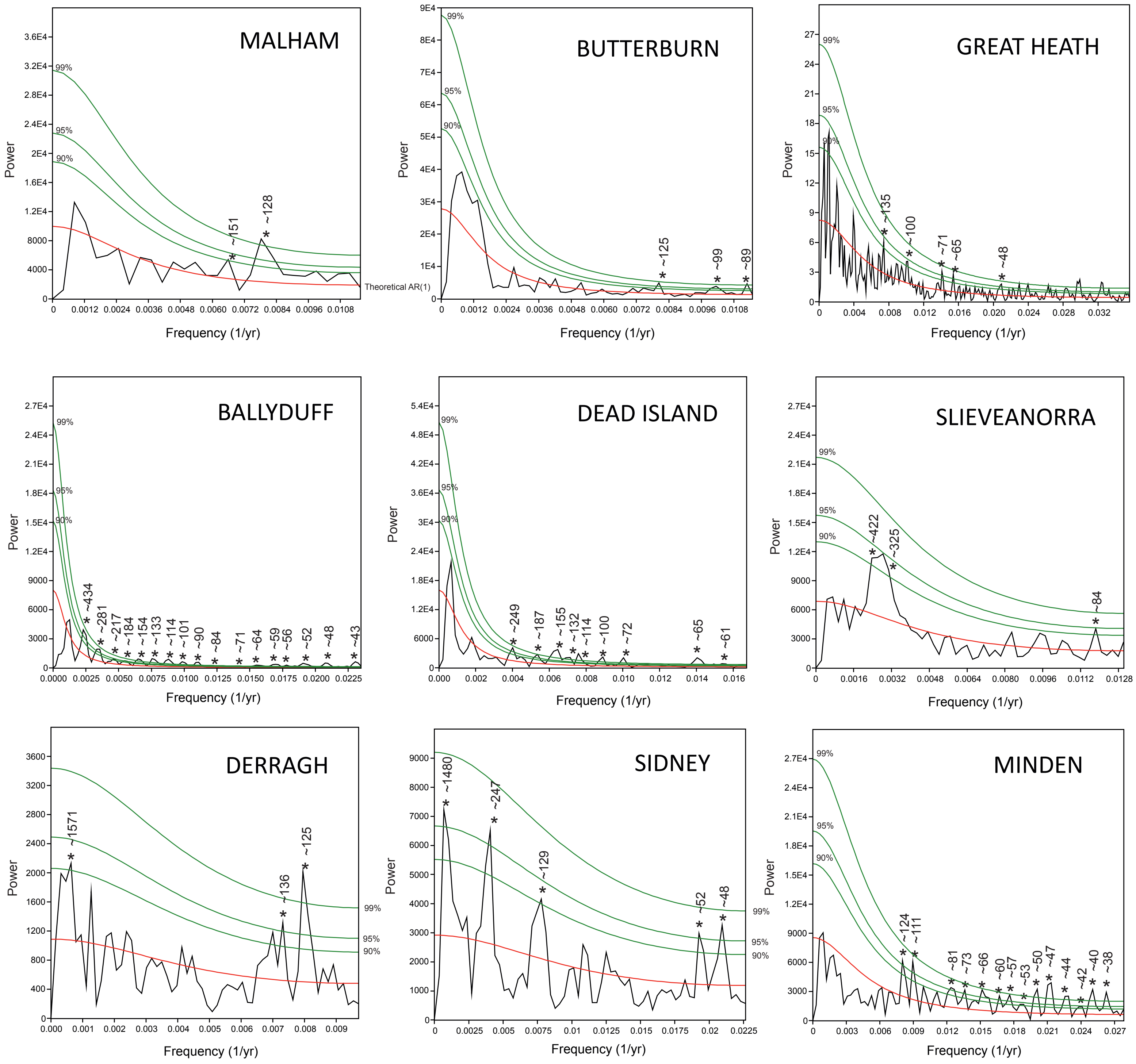

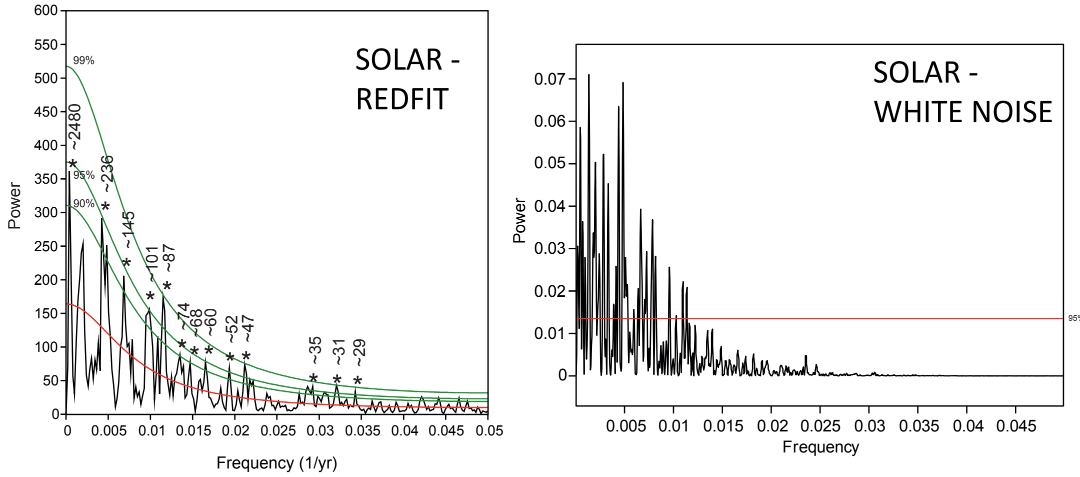

Significant periodicities		
6613	484	195
4960	422	189
3968	413	156
2480	405	151
2204	361	150
1984	354	149
1804	348	148
1417	342	147
1323	305	139
1044	301	138
992	296	129
794	242	128
763	231	127
735	228	126
709	225	123
684	223	122
567	209	122
551	207	104
536	205	104
522	202	91
509	198	91
496	196	88

5 Wavelet analysis

These figures show [top panel] continuous wavelet analysis of the sunspot reconstruction of Solanki et al. (2004), and [middle panel] normalised water table reconstruction from each site. The bottom panel shows the cross-wavelet analysis of the top and middle panels. The black lines signify 95 % significant levels against a lag1 (red noise) background. Each sites proxy data (‘real data’) is shown first, followed by the same analysis conducted on the random walk data (site name suffixed with ‘RW’).

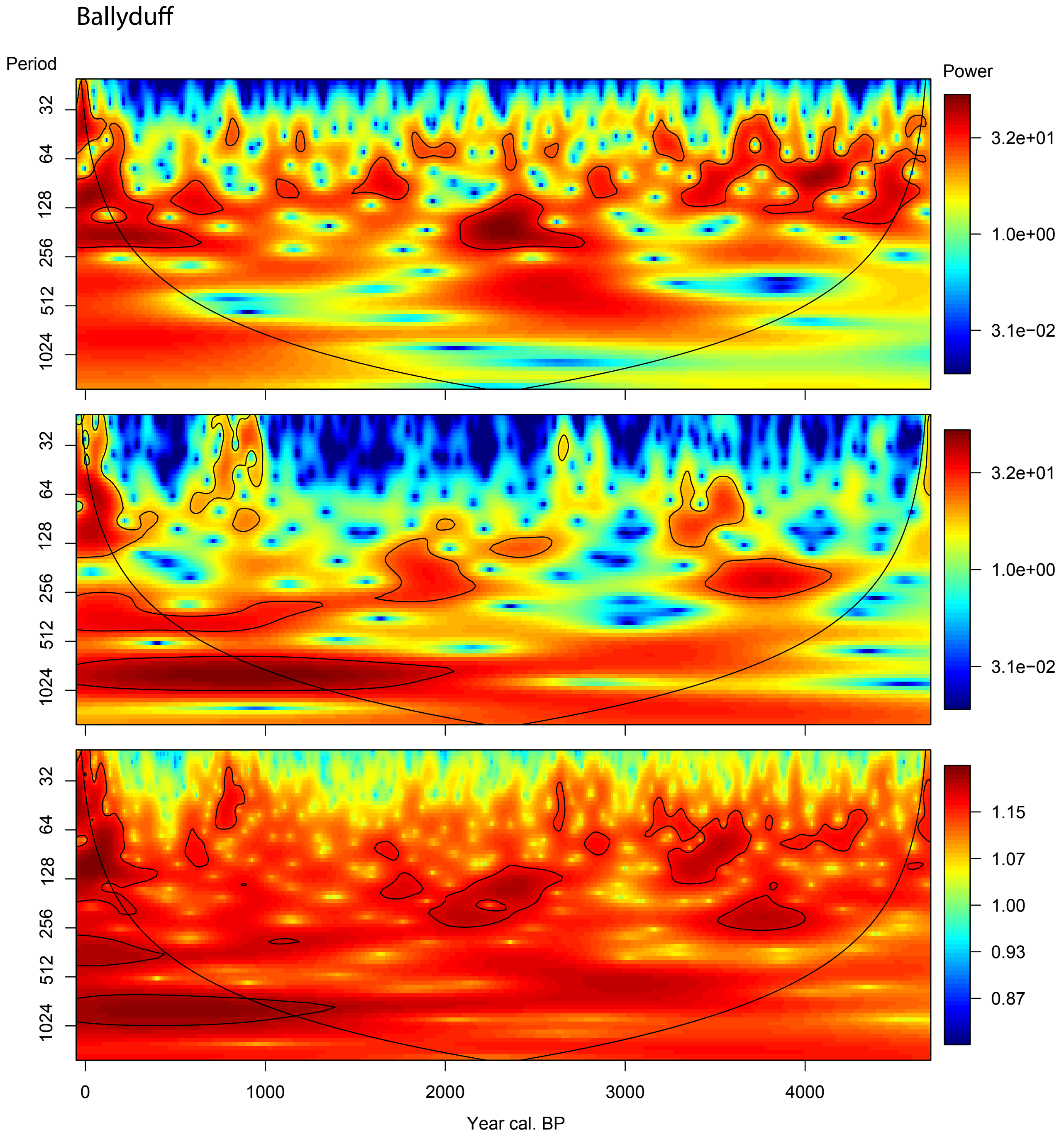

Ballyduff
Period
Power
32
64
128
256
512
1024
3.2e+01
1.0e+00
3.1e−02
1.15
1.07
1.00
0.93
0.87
0
1000
2000
3000
4000
Year cal. BP

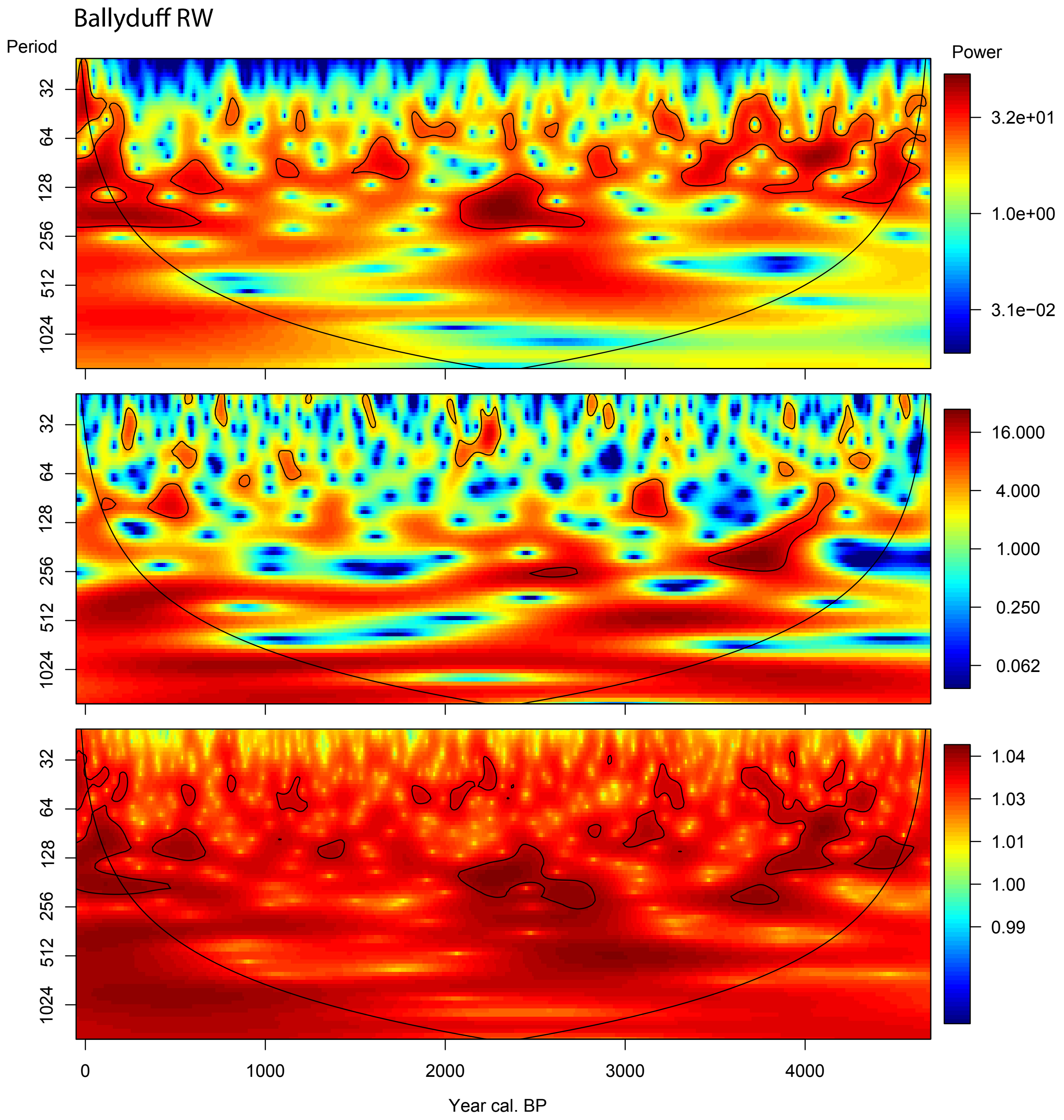
Ballyduff RW
Period
Power
32
64
128
256
512
1024
3.2e+01
1.0e+00
3.1e−02
16.000
4.000
1.000
0.250
0.062
1.04
1.03
1.01
1.00
0.99
0
1000
2000
3000
4000
Year cal. BP

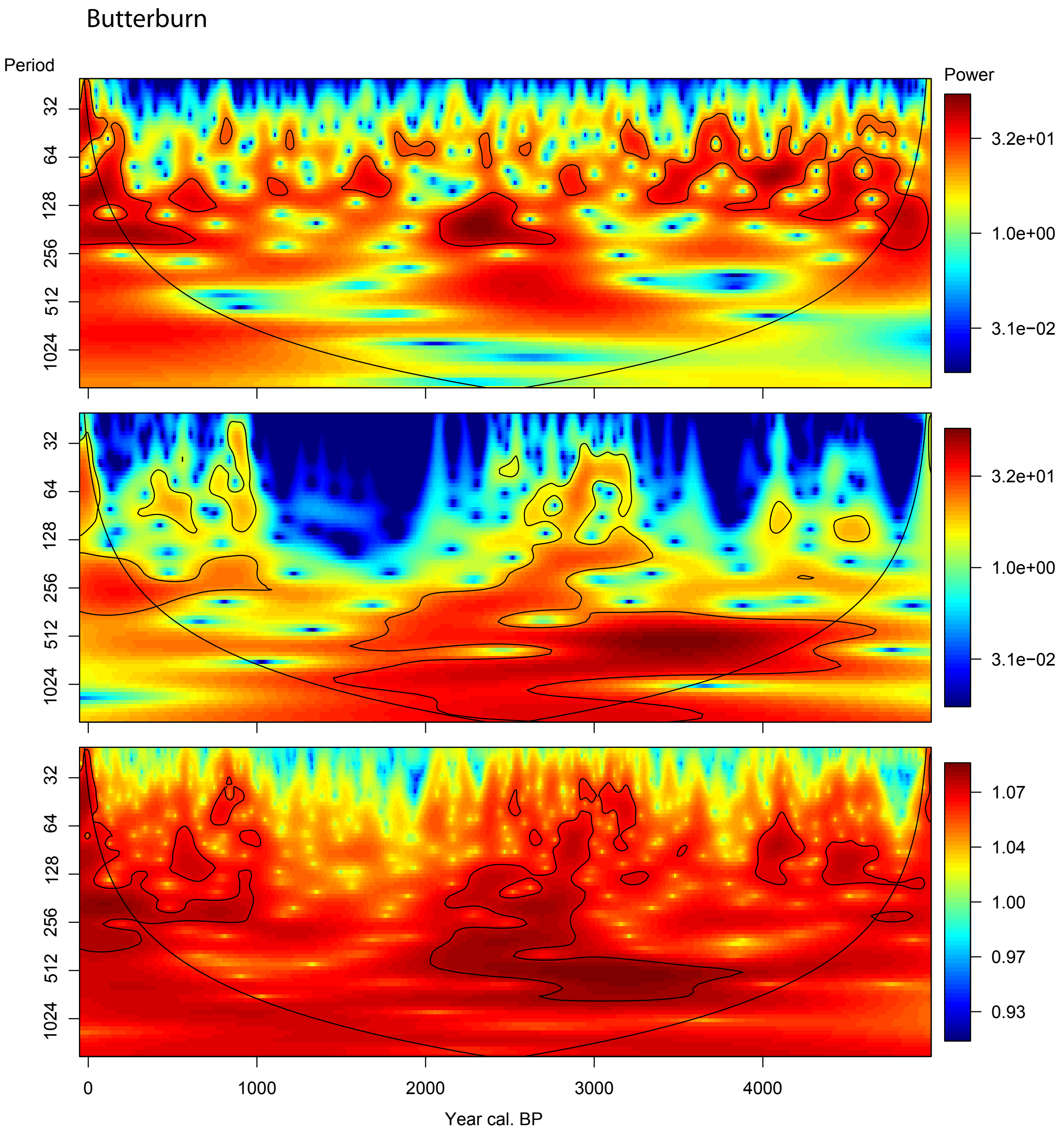
Butterburn
Period
Power
32
64
128
256
512
1024
3.2e+01
1.0e+00
3.1e−02
1.07
1.04
1.00
0.97
0.93
0
1000
2000
3000
4000
Year cal. BP

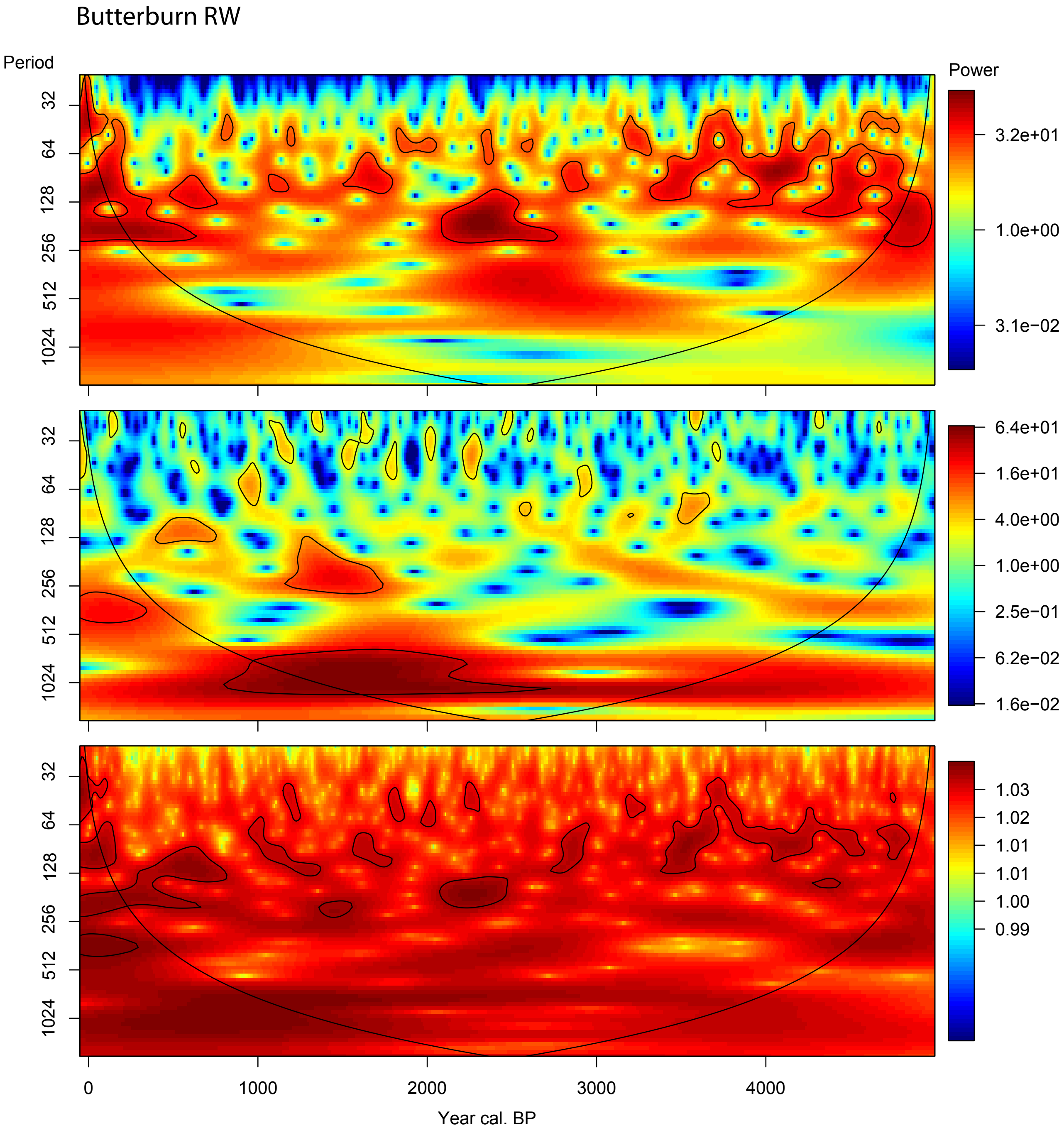
Butterburn RW
Period
Power
32
64
128
256
512
1024
3.2e+01
1.0e+00
3.1e−02
6.4e+01
1.6e+01
4.0e+00
1.0e+00
2.5e−01
6.2e−02
1.6e−02
1.03
1.02
1.01
1.01
1.00
0.99
0
1000
2000
3000
4000
Year cal. BP

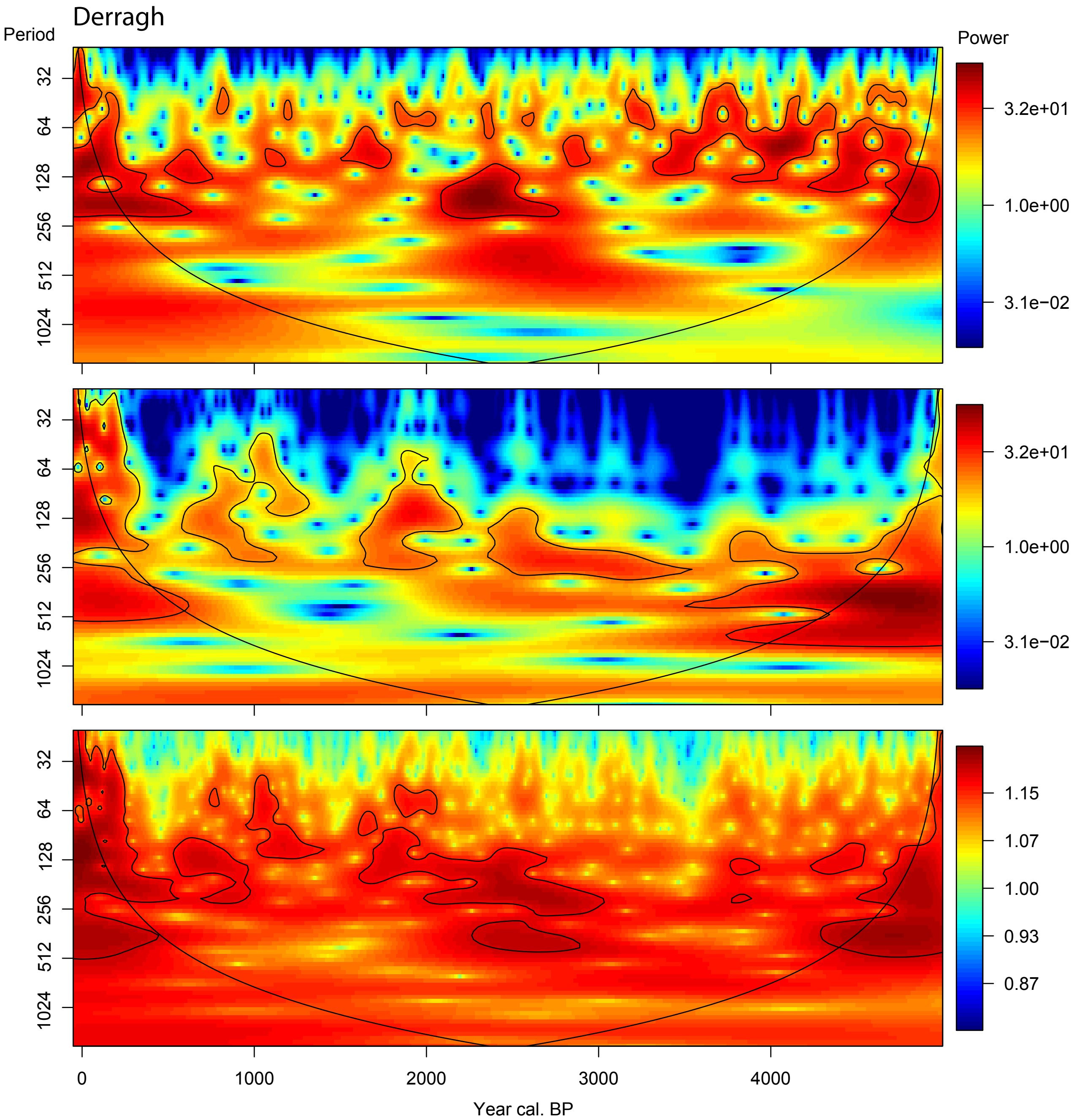

Derragh
Period
Power
32
64
128
256
512
1024
3.2e+01
1.0e+00
3.1e−02
1.15
1.07
1.00
0.93
0.87
0
1000
2000
3000
4000
Year cal. BP

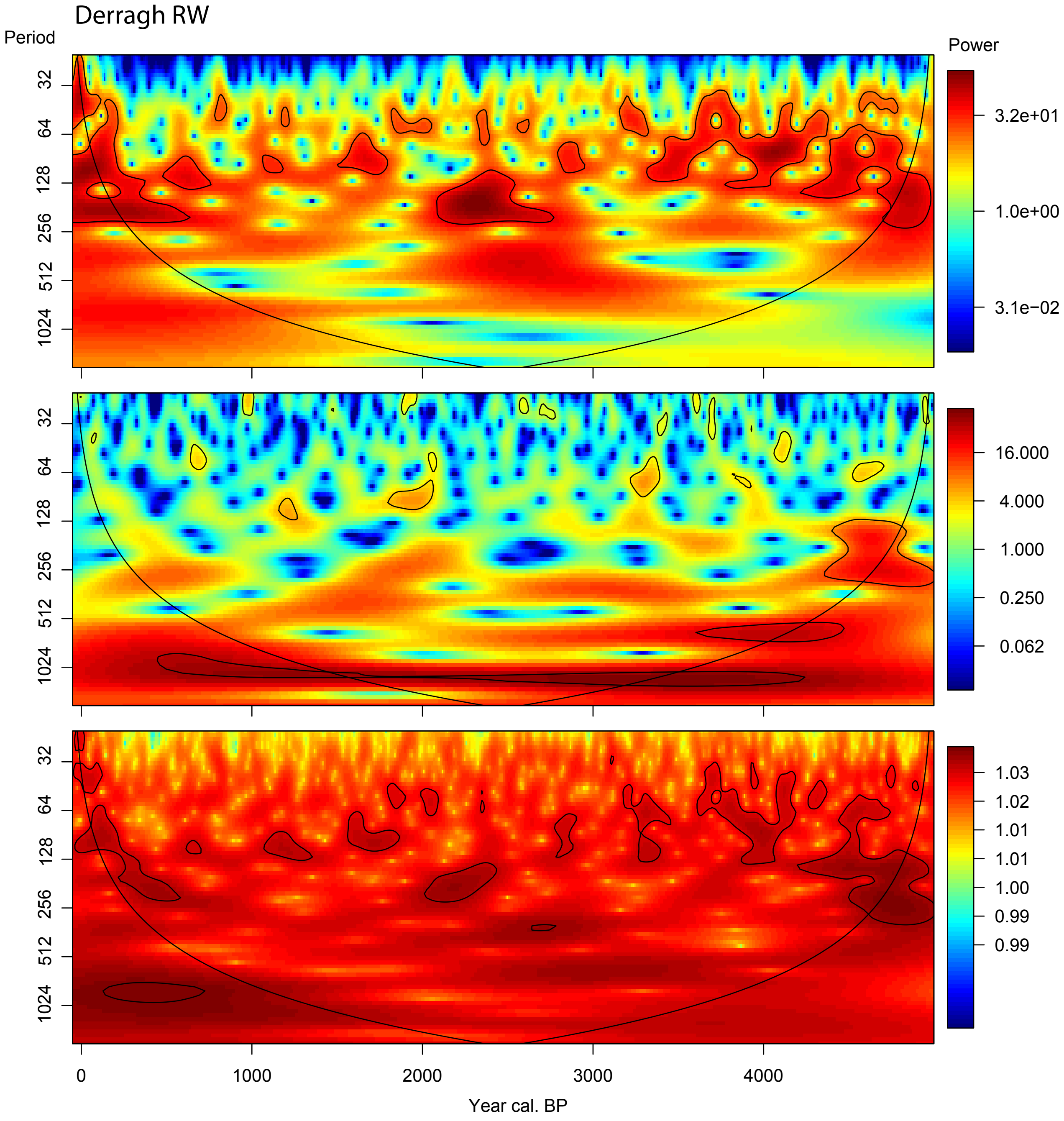

Derragh RW
Period
Power
32
64
128
256
512
1024
3.2e+01
1.0e+00
3.1e−02
16.000
4.000
1.000
0.250
0.062
1.03
1.02
1.01
1.01
1.00
0.99
0.99
0
1000
2000
3000
4000
Year cal. BP

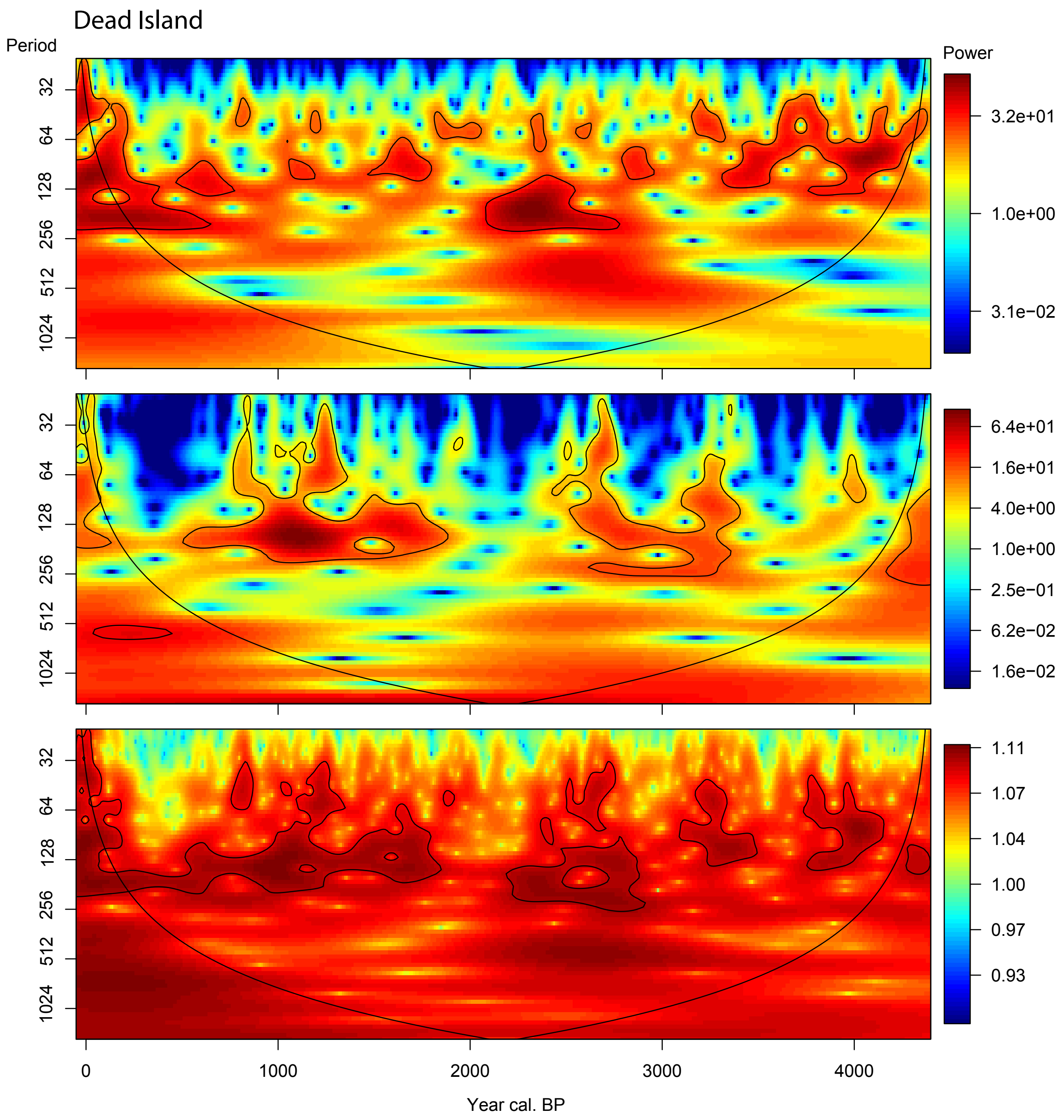
Dead Island
Period
32
64
128
256
512
1024
Power
3.2e+01
1.0e+00
3.1e−02
6.4e+01
1.6e+01
4.0e+00
1.0e+00
2.5e−01
6.2e−02
1.6e−02
1.11
1.07
1.04
1.00
0.97
0.93
0
1000
2000
3000
4000
Year cal. BP

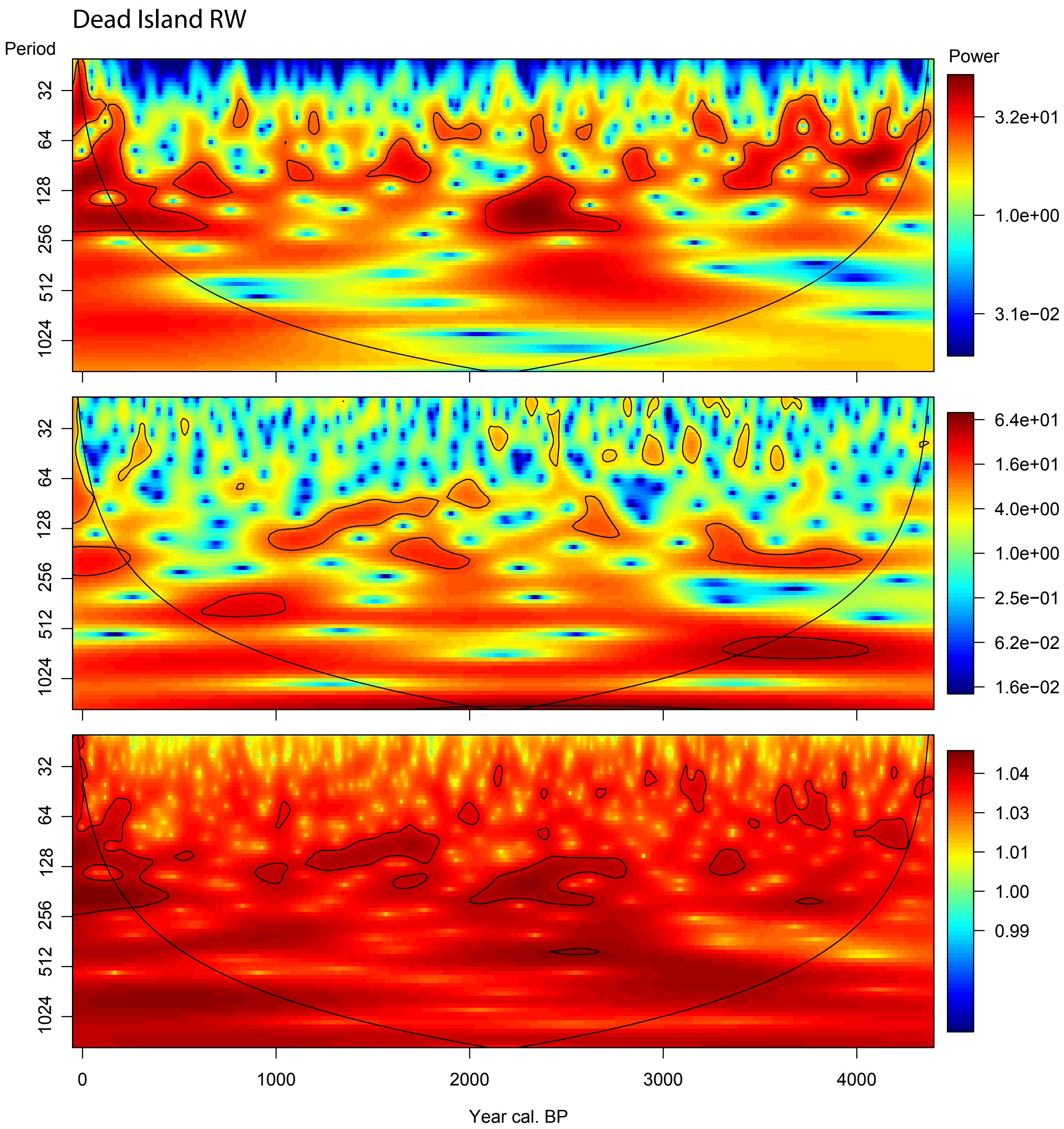

Dead Island RW
Period
Power
32
64
128
256
512
1024
3.2e+01
1.0e+00
3.1e−02
6.4e+01
1.6e+01
4.0e+00
1.0e+00
2.5e−01
6.2e−02
1.6e−02
1.04
1.03
1.01
1.00
0.99
0
1000
2000
3000
4000
Year cal. BP

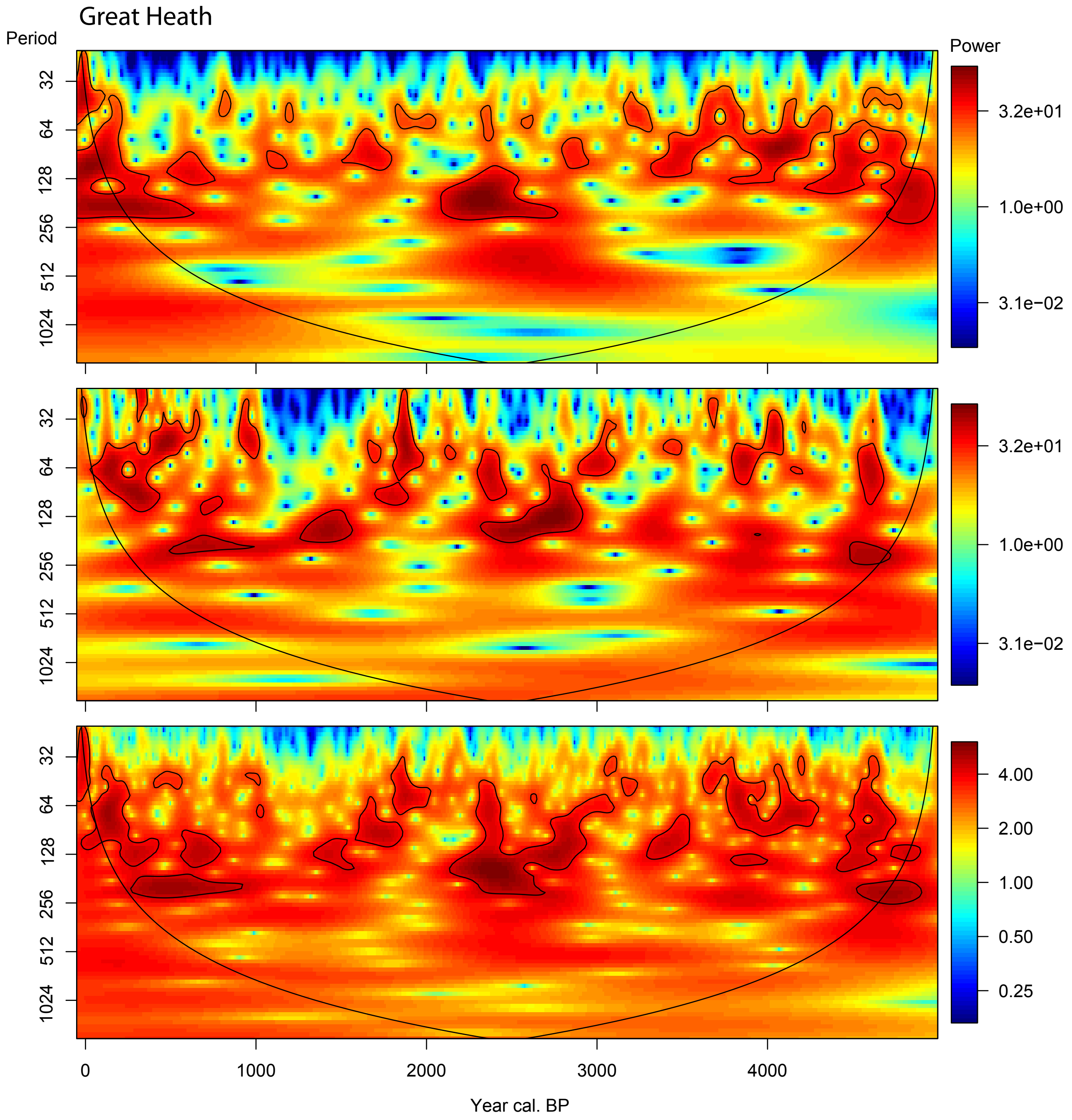

Great Heath
Period
Power
32
64
128
256
512
1024
3.2e+01
1.0e+00
3.1e−02
4.00
2.00
1.00
0.50
0.25
0
1000
2000
3000
4000
Year cal. BP

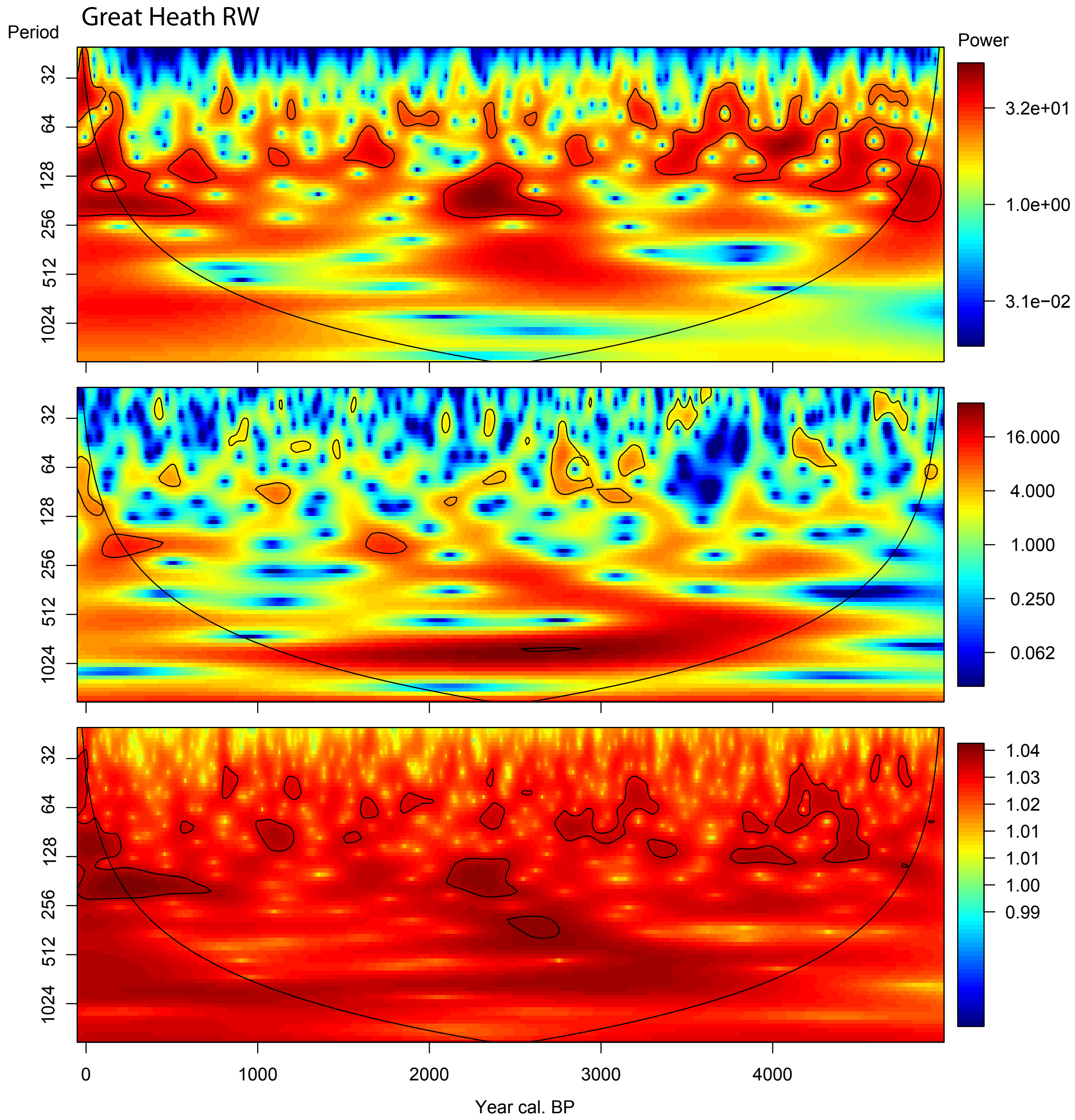

Great Heath RW
Period
Power
32
64
128
256
512
1024
3.2e+01
1.0e+00
3.1e−02
16.000
4.000
1.000
0.250
0.062
1.04
1.03
1.02
1.01
1.01
1.00
0.99
0
1000
2000
3000
4000
Year cal. BP

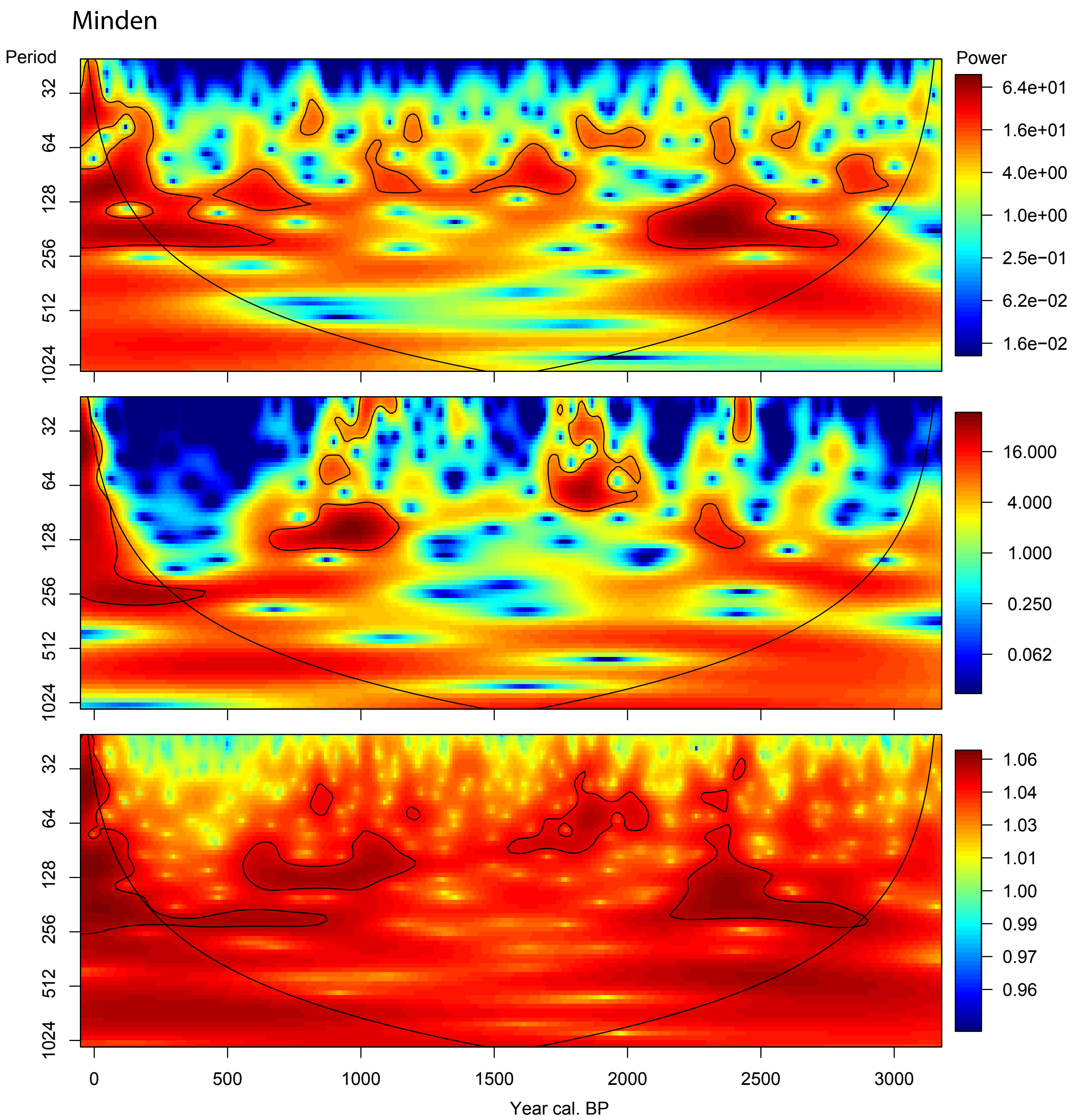

Minden
Period
Power
32
64
128
256
512
1024
6.4e+01
1.6e+01
4.0e+00
1.0e+00
2.5e−01
6.2e−02
1.6e−02
16.000
4.000
1.000
0.250
0.062
1.06
1.04
1.03
1.01
1.00
0.99
0.97
0.96
0
500
1000
1500
2000
2500
3000
Year cal. BP

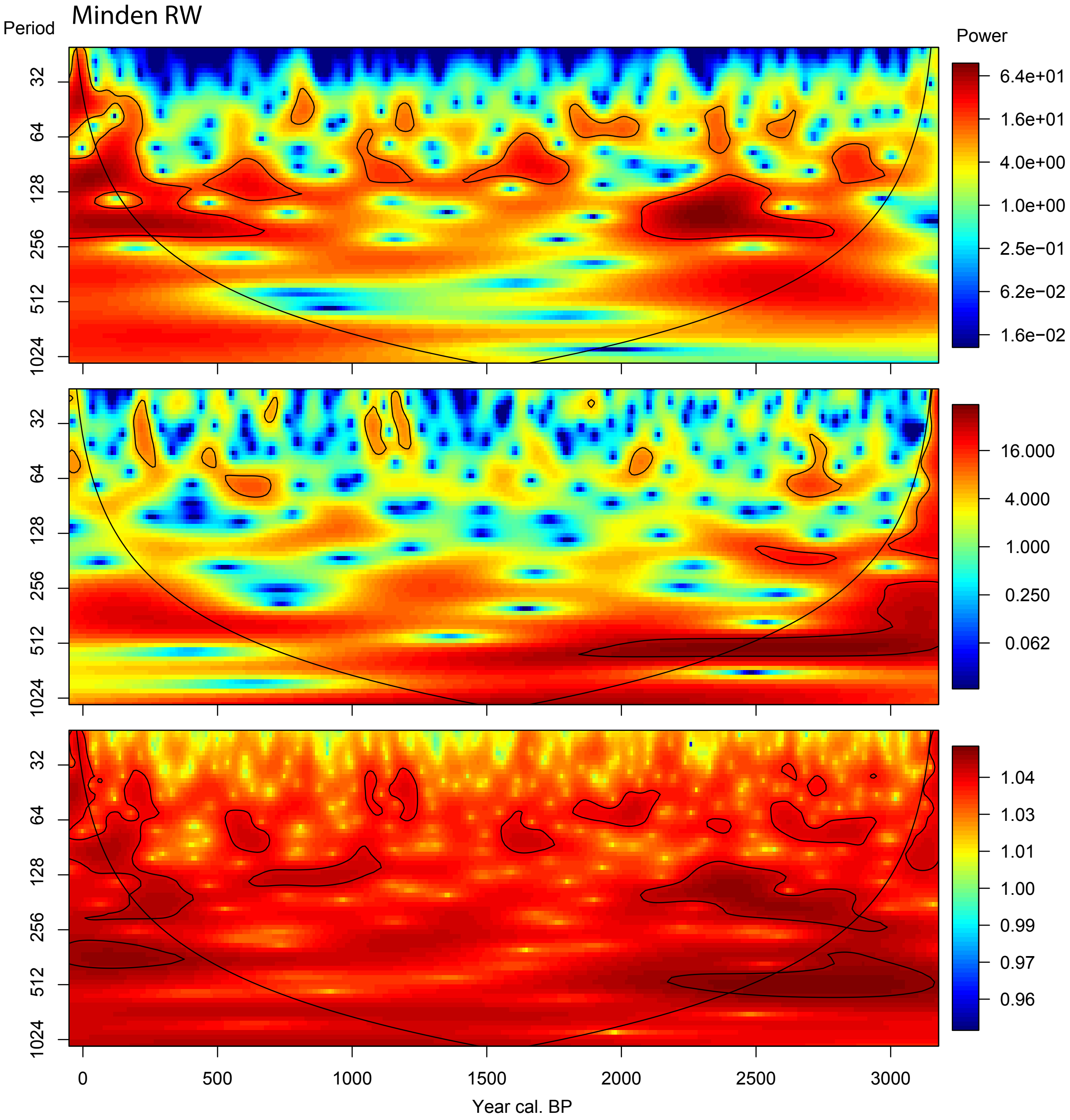
Minden RW
Period
Power
32
64
128
256
512
1024
6.4e+01
1.6e+01
4.0e+00
1.0e+00
2.5e−01
6.2e−02
1.6e−02
16.000
4.000
1.000
0.250
0.062
1.04
1.03
1.01
1.00
0.99
0.97
0.96
0
500
1000
1500
2000
2500
3000
Year cal. BP

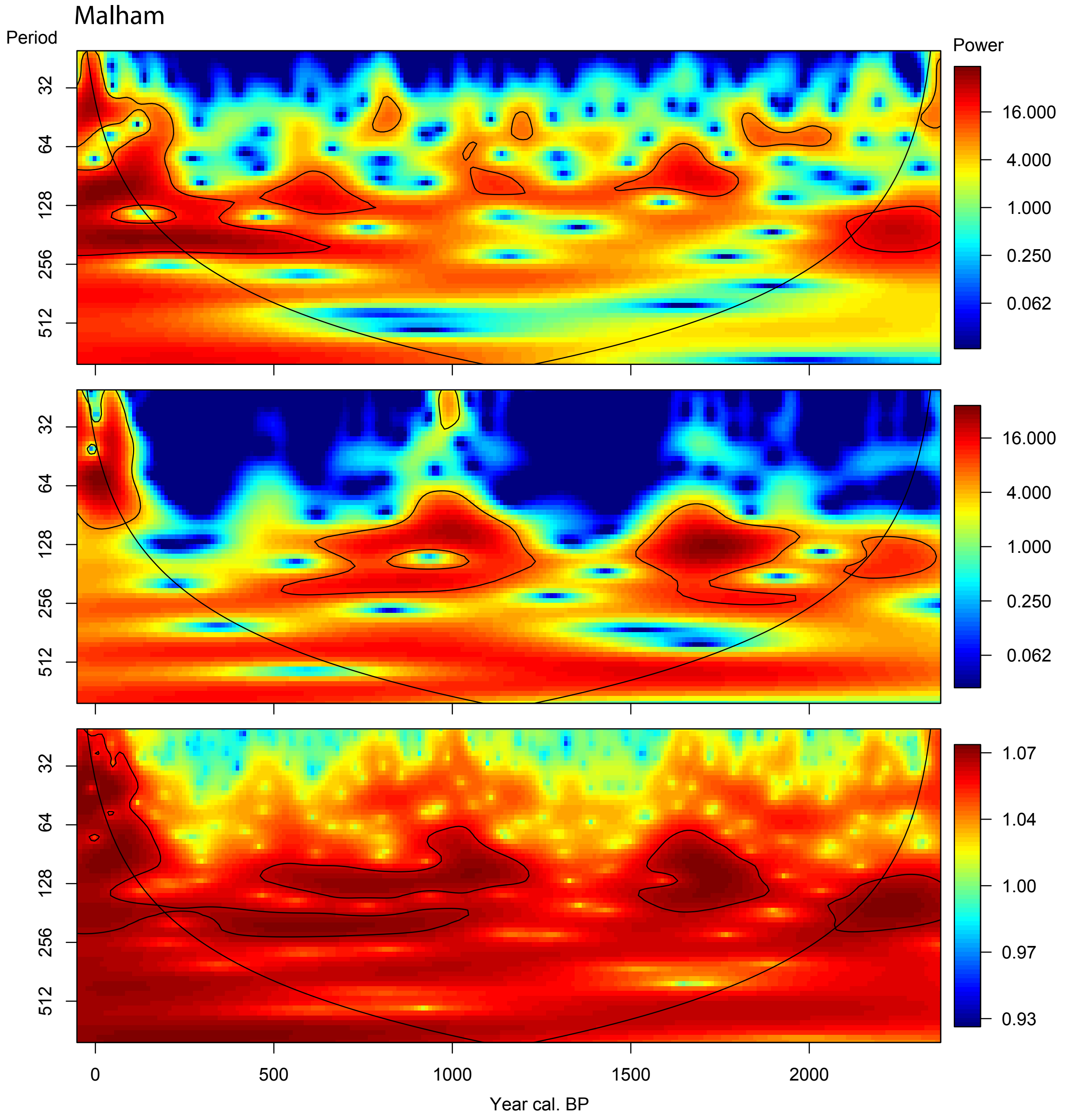
Malham
Period
Power
32
64
128
256
512
16.000
4.000
1.000
0.250
0.062
1.07
1.04
1.00
0.97
0.93
0
500
1000
1500
2000
Year cal. BP

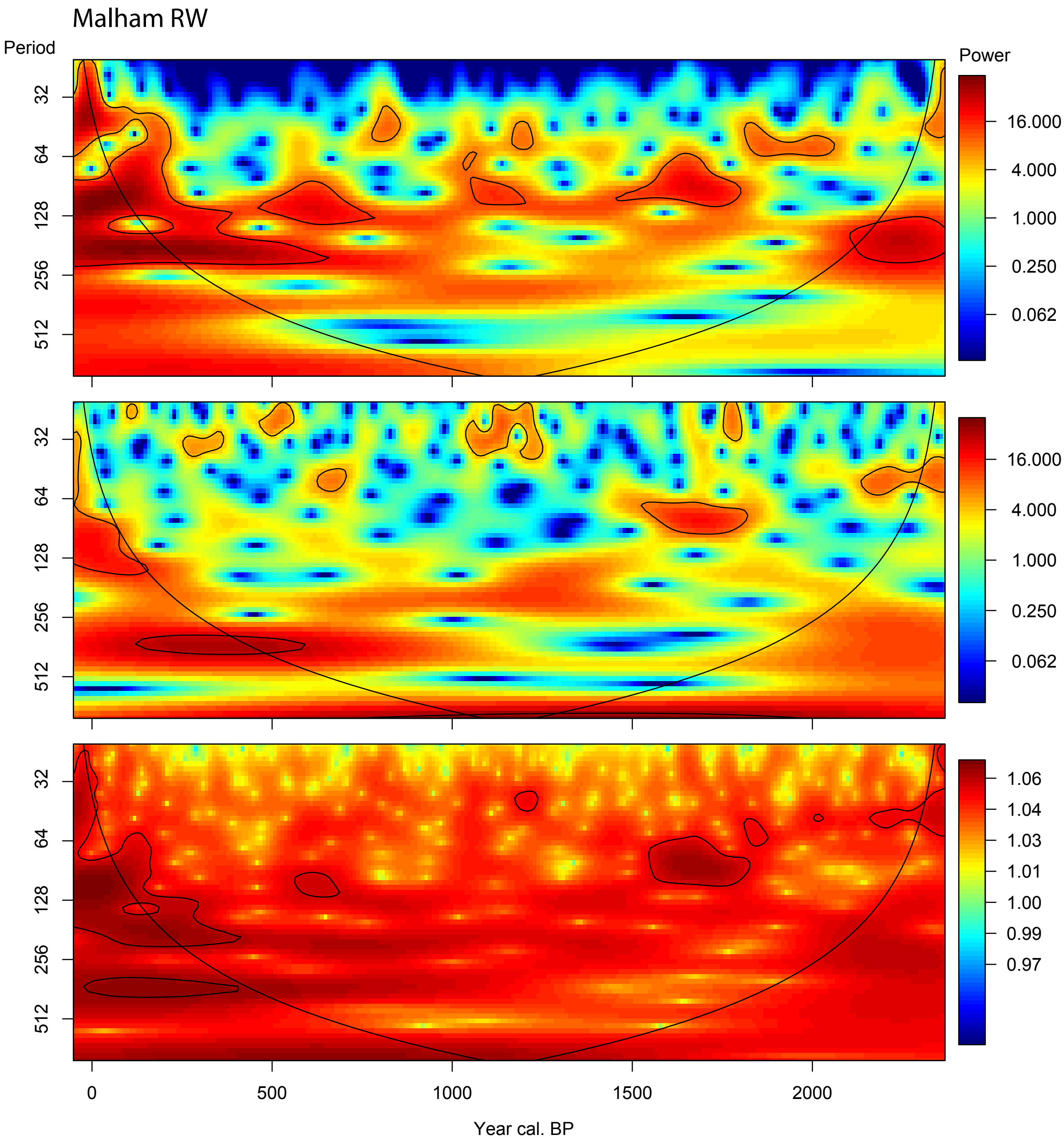

Malham RW
Period
Power
32
64
128
256
512
16.000
4.000
1.000
0.250
0.062
1.06
1.04
1.03
1.01
1.00
0.99
0.97
0
500
1000
1500
2000
Year cal. BP

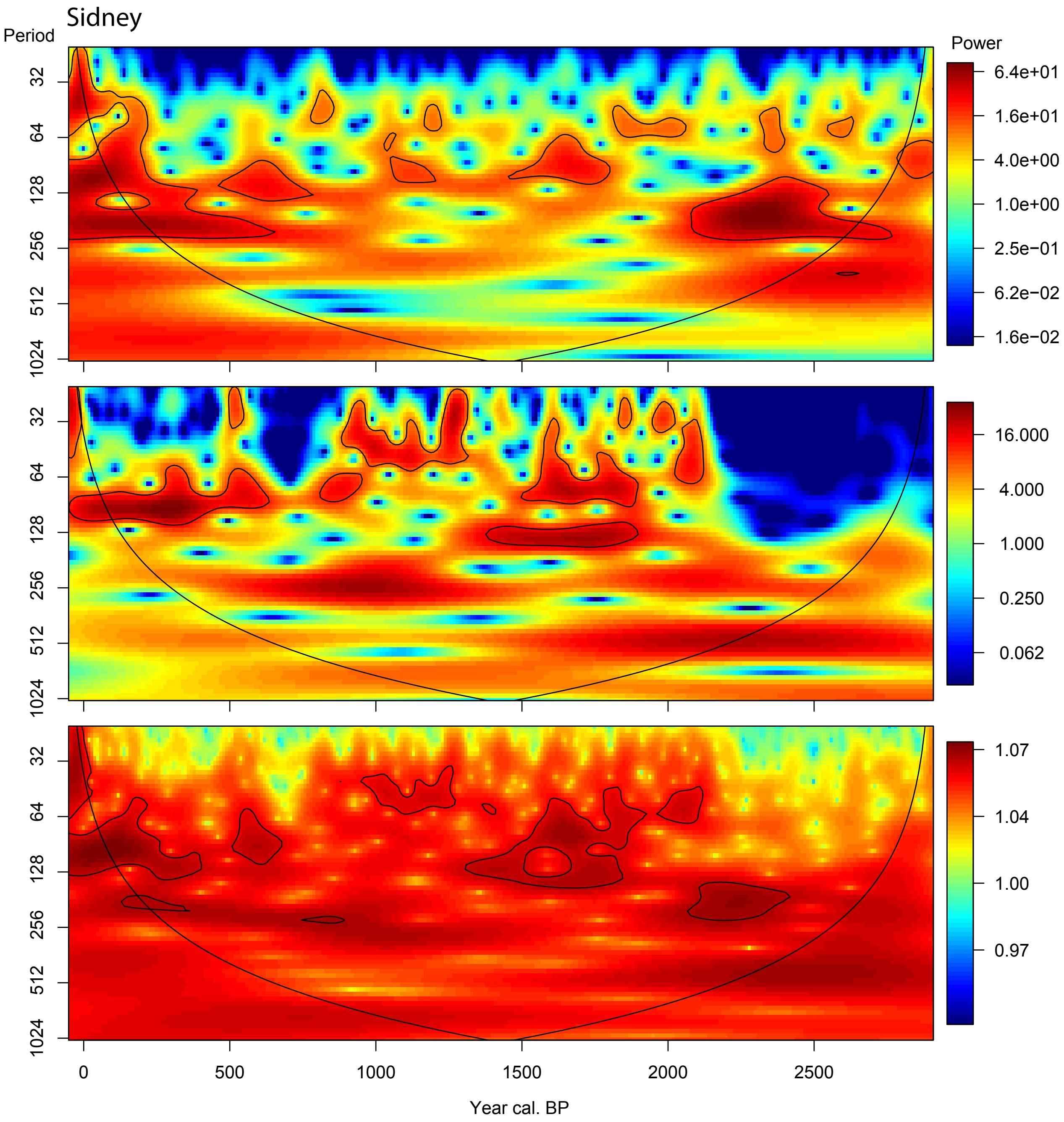

Sidney
Period
Power
32
64
128
256
512
1024
6.4e+01
1.6e+01
4.0e+00
1.0e+00
2.5e−01
6.2e−02
1.6e−02
16.000
4.000
1.000
0.250
0.062
1.07
1.04
1.00
0.97
0
500
1000
1500
2000
2500
Year cal. BP

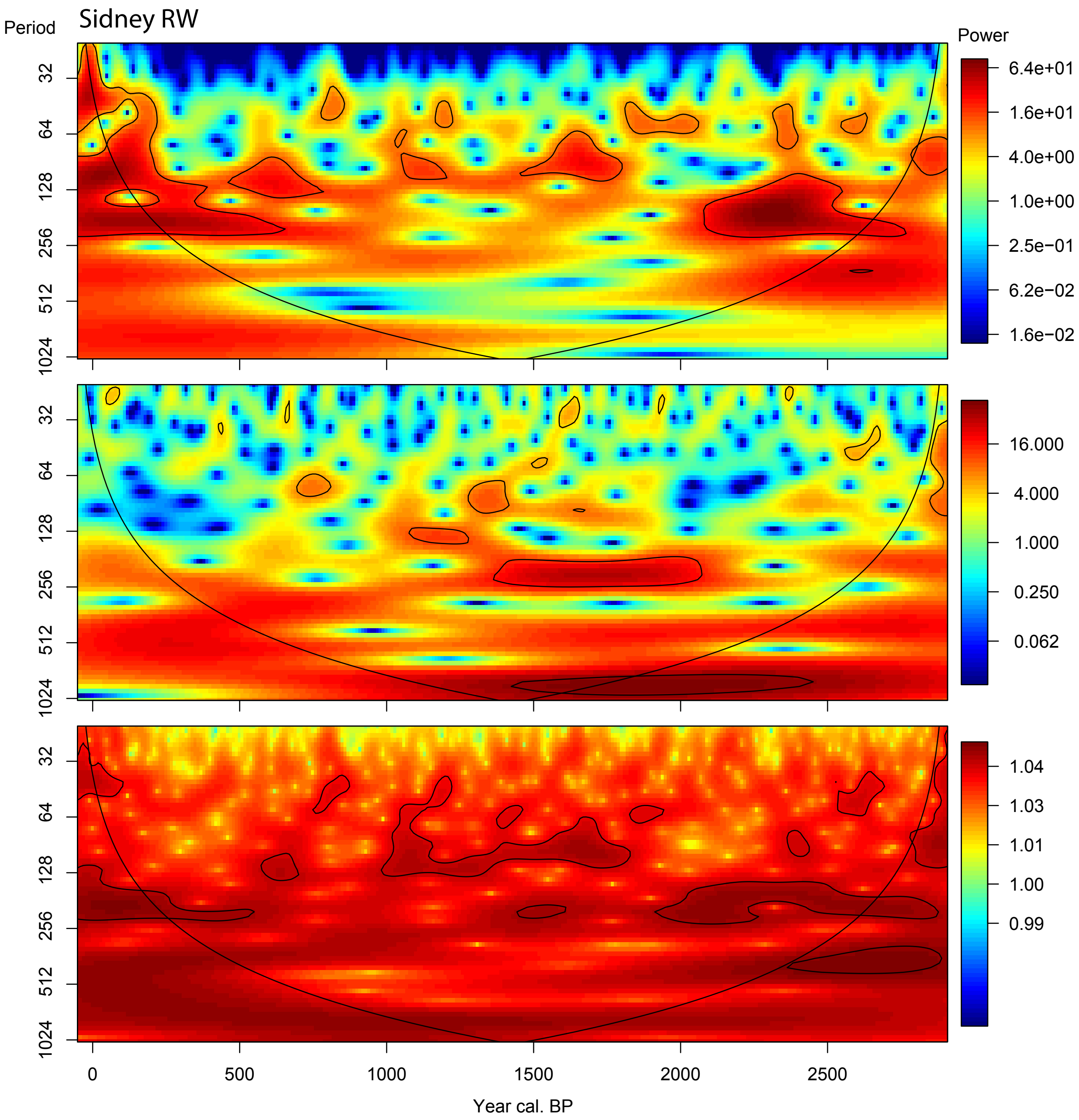

Sidney RW
Period
Power
32
64
128
256
512
1024
6.4e+01
1.6e+01
4.0e+00
1.0e+00
2.5e−01
6.2e−02
1.6e−02
16.000
4.000
1.000
0.250
0.062
1.04
1.03
1.01
1.00
0.99
0
500
1000
1500
2000
2500
Year cal. BP

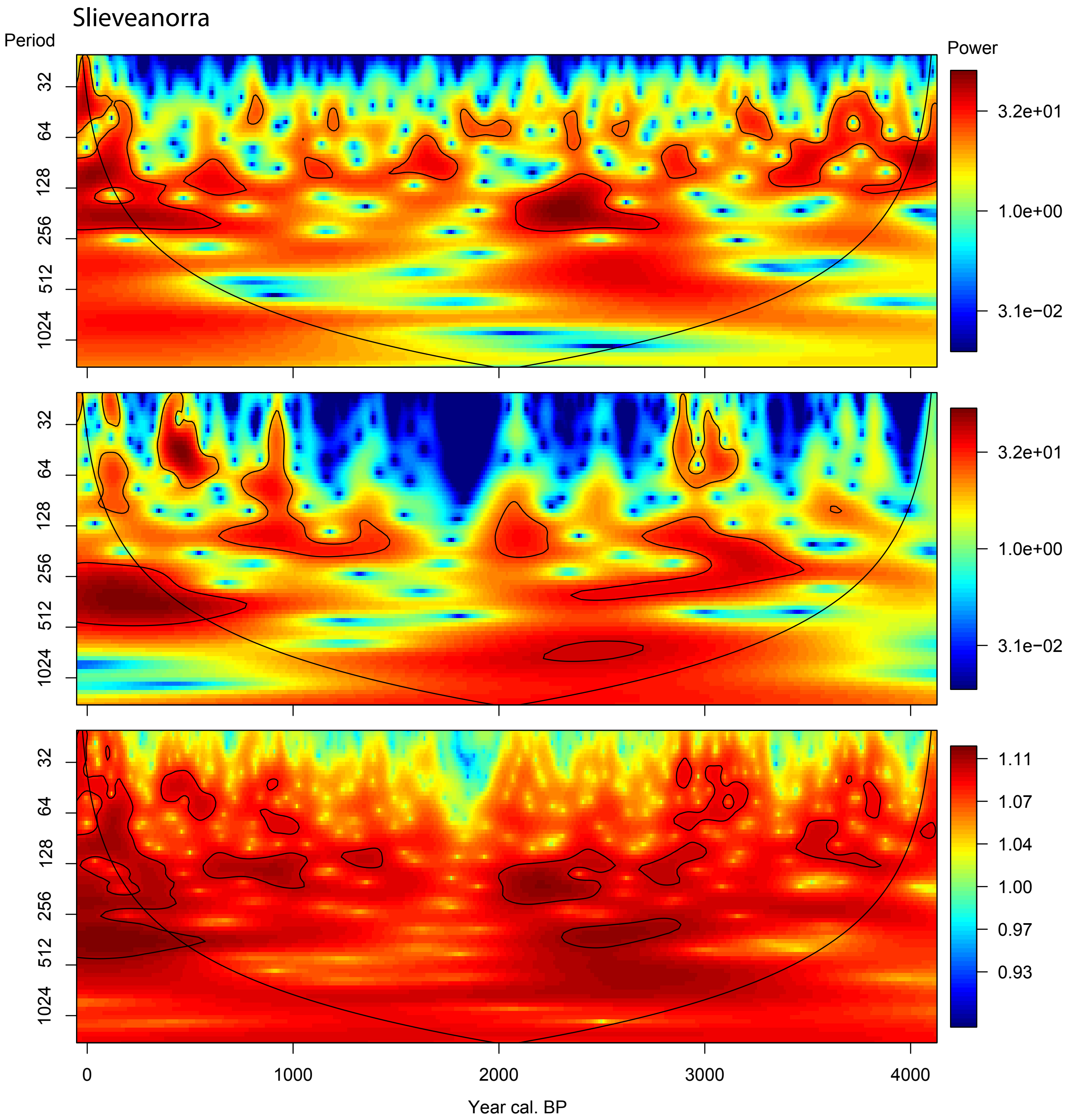
Slieveanorra
Period
Power
32
64
128
256
512
1024
3.2e+01
1.0e+00
3.1e-02
1.11
1.07
1.04
1.00
0.97
0.93
0
1000
2000
3000
4000
Year cal. BP

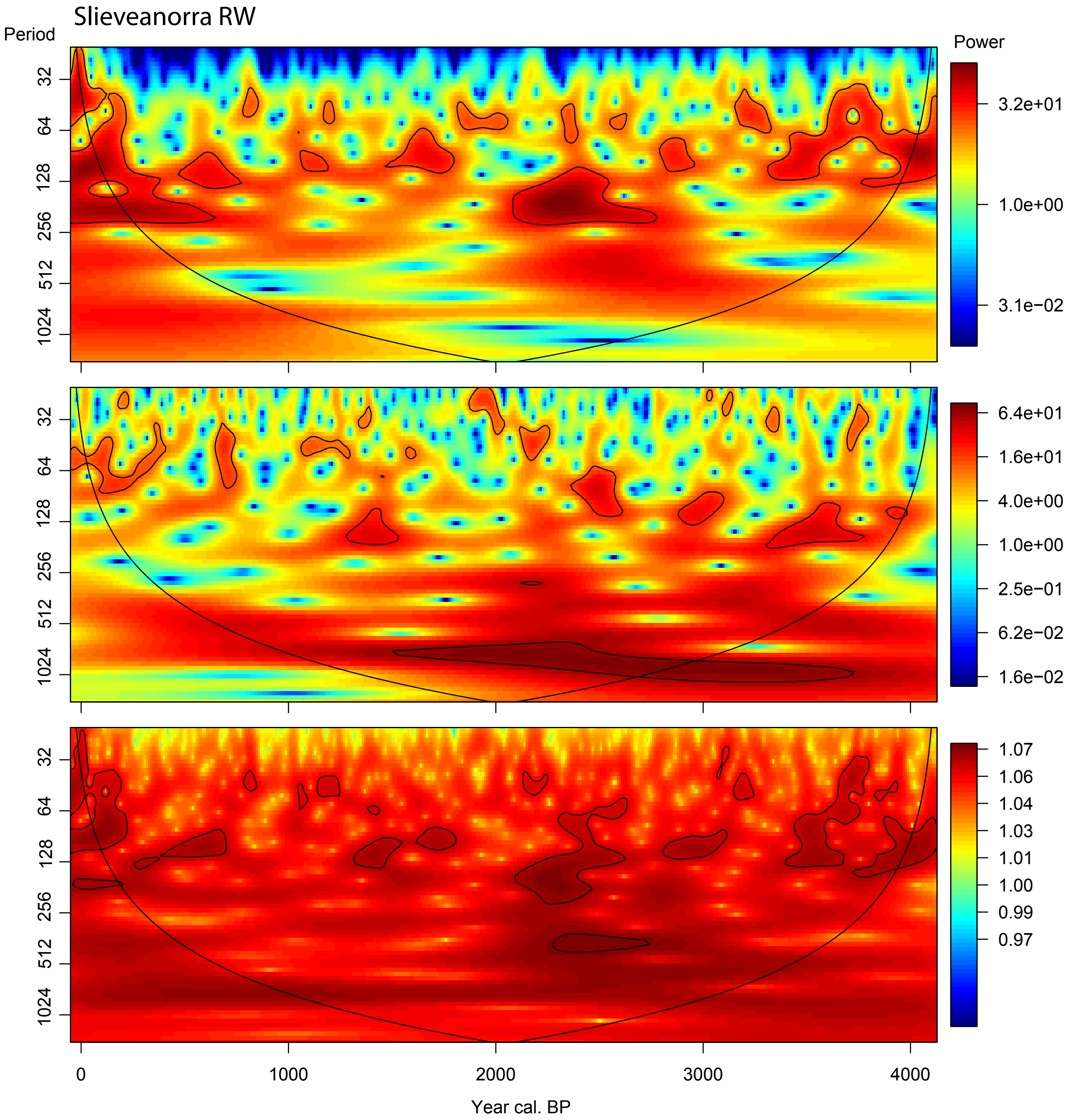

Slieveanorra RW
Period
Power
32
64
128
256
512
1024
3.2e+01
1.0e+00
3.1e-02
6.4e+01
1.6e+01
4.0e+00
1.0e+00
2.5e-01
6.2e-02
1.6e-02
1.07
1.06
1.04
1.03
1.01
1.00
0.99
0.97
0
1000
2000
3000
4000
Year cal. BP

6 Literature survey of periodicities reported in the literature

Significant periodicities in peatlands from published literature

#	Lead author	Period	Period	Period	Period	Period	Period	Period	Period	Period	Period	Period	Period	Period	Period
1	Swindles	1120	1050	750	540	500	260	250	180	130					
2	Swindles	1530	1200	580	440	370	320	290	260	180	170	130			
3	Swindles	580	423	391	373	307	265								
4	Turner	1230	240	176	150	130	80	60							
5	Langdon	1100													
6	Hughes	1100	600												
7	Aaby	520	260												
8	Blundell	560													
9	Nichols	205	129												
10	Barber	800													
11	Borgmark	1024	882	661	258	231	196								
12	Borgmark	2020	900	396	310	248									
19	Hong	86	93	101	110	127	132	140	155	207	245	311	590	820	1046
20	Chambers	80	120	200											
21	Chambers	60	100	200											
22	Chambers	80													
23	Chambers	70													
24	Chambers	210													
25	Oliver	800													
26	Yu	2190	1665	1500	667	386									

Histograms of significant periodicities: top (0-1000 year periods), bottom (0-500 year periods)

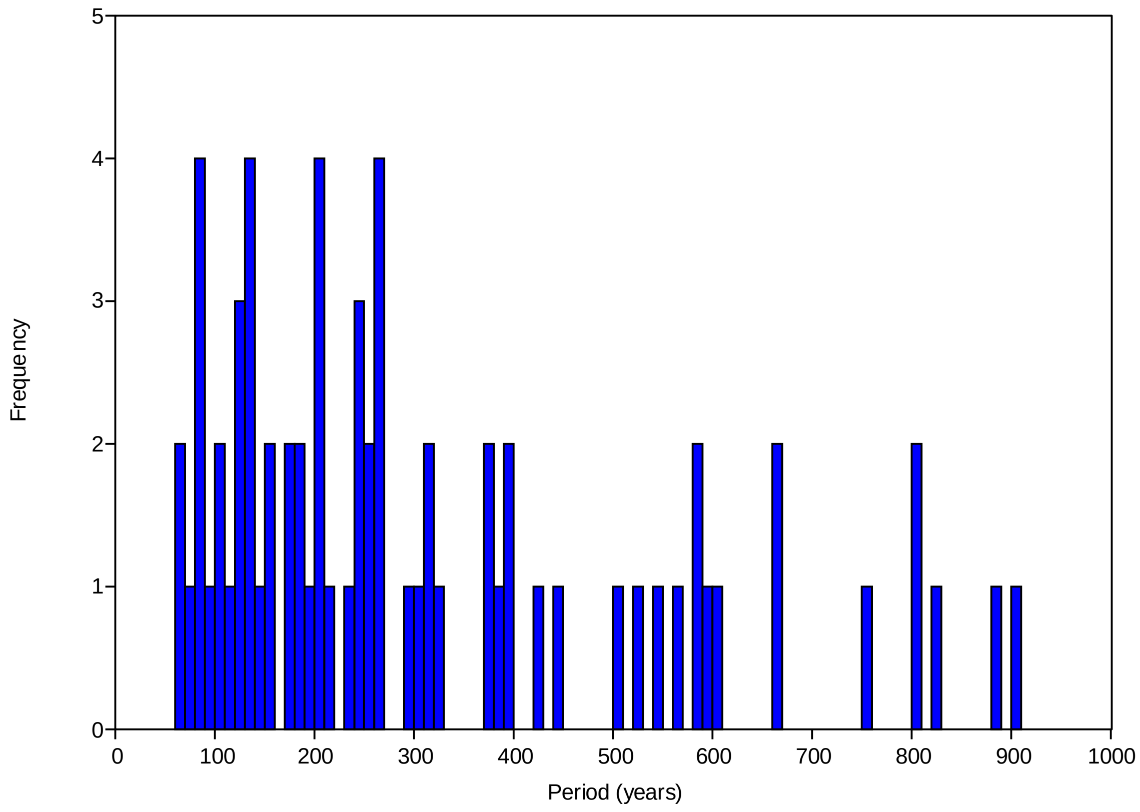

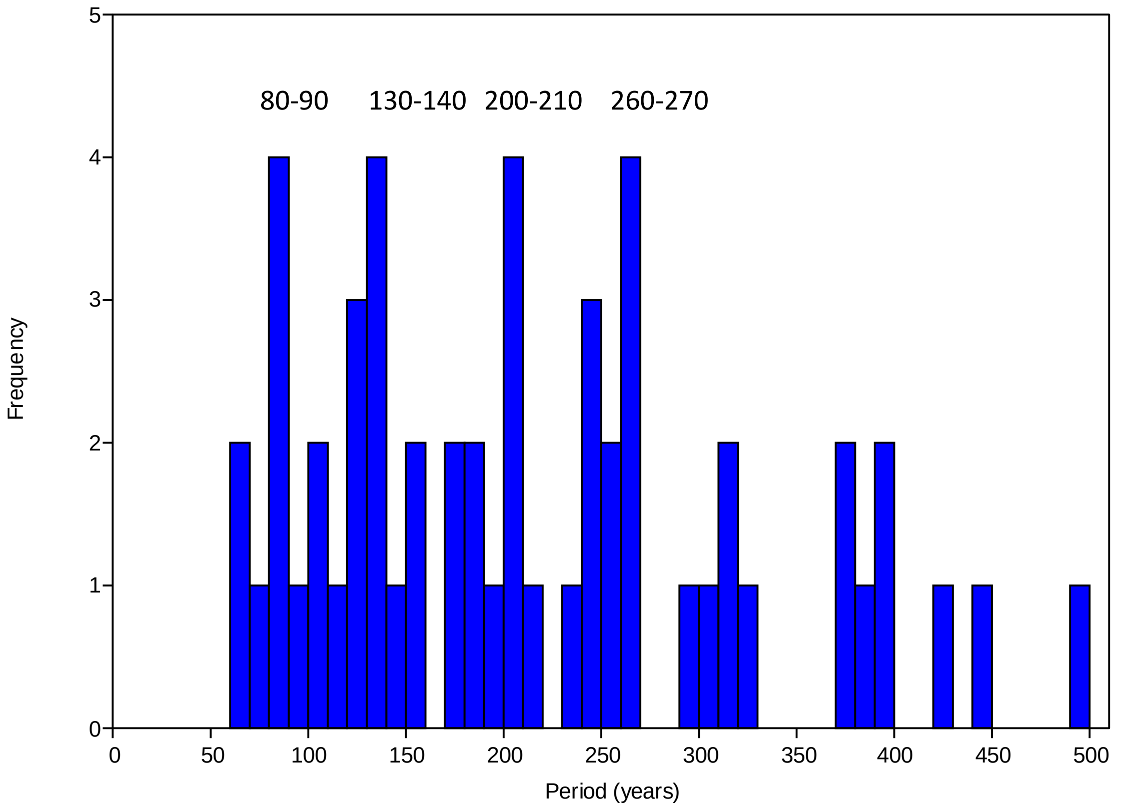

Sources and meta-data for Supplementary material

#	Reference	Country	Site	Latitude	Longitude	Noise test	Resolution	Chronological control	Significance testing	Comments
1	Swindles et al. (2012) Quaternary Science Reviews 41, 94-103.	Ireland	Dead Island	54.887821	-6.549955	Red	Multi-centennial	Well dated for periodicities present	Yes	Siegel test, REDFIT
2	Swindles et al. (2012) Quaternary Science Reviews 41, 94-103.	Ireland	Slieveanorra	55.085101	-6.192297	Red	Multi-centennial	Well dated for periodicities present	Yes	Siegel test, REDFIT
3	Swindles et al. (2007) Journal of Quaternary Science 22, 667–679.	Ireland	Glen West	54.409342	-8.036475	None	Multi-centennial	Satisfactorily dated for periodicities present	Yes	Siegel test
4	Turner et al. (2014) Quaternary Science Reviews 84, 65-85.	England	Malham	54.097257	-2.172562	Red	Multi-centennial	Well dated for periodicities present	Yes	REDFIT
5	Langdon et al. (2003) Quaternary Science Reviews 22, 259-274.	Scotland	Temple Hill	55.844318	-3.426220	None	Multi-centennial	Well dated for periodicities present	No	Simple periodogram
6	Hughes et al. (2000) The Holocene 10, 465-479.	England	Walton Moss	54.991179	-2.773580	None	Multi-centennial	Satisfactorily dated for periodicities present	No	Simple periodogram
7	Aaby (1976) Nature 263, 281-284.	Denmark	Draved Moss	55.016700	8.933000	None	Multi-centennial	Satisfactorily dated for periodicities present	No	Timing between shifts
8	Blundell and Barber (2005) Quaternary Science Reviews 24,	Scotland	Tore Hill	57.232538	-3.674747	None	Multi-centennial	Satisfactorily dated for periodicities	No	Simple periodogram

	1261-1277							present		
9	Nichols and Hyuang(2012) Geophysical Research Letters 39, L04707.	USA	The Great Heath	44.708848	-67.810639	Red	Multi-centennial	Well dated for periodicities present	Yes	Spectral and Cross-spectral
10	Barber et al. (1994) The Holocene 4, 198-205.	England	Bolton Fell Moss	55.012129	-2.801009	None	Multi-centennial	Satisfactorily dated for periodicities present	No	Simple periodogram
11	Borgmark (2005) The Holocene 15, 387-395.	Sweden	Stomyren	60.790634	12.720518	Red	Multi-centennial	Well dated for periodicities present	Yes	Siegel test, REDFIT
12	Borgmark (2005) The Holocene 15, 387-395.	Sweden	Kortlandamossen	59.81993	12.234373	Red	Multi-centennial	Well dated for periodicities present	Yes	Siegel test, REDFIT
19	Hong et al. (2000) The Holocene 10, 1-7	China	Jinchuan	42.333333	126.366667	None	Multi-centennial	Marginally dated for periodicities present	No	Simple periodogram
20	Chambers and Blackford (2001) Journal of Quaternary Science 16, 329-338.	Wales	Migneint	52.970278	-3.840556	Red	Multi-decadal	Marginally dated for periodicities present	Yes	Fourier transform
21	Chambers and Blackford (2001) Journal of Quaternary Science 16, 329-338.	England	Harold's bog	54.326389	-1.100556	Red	Multi-decadal	Marginally dated for periodicities present	Yes	Fourier transform
22	Chambers and Blackford (2001) Journal of Quaternary Science 16, 329-338.	Ireland	Letterfrack	53.559124	-9.946773	Red	Multi-decadal	Marginally dated for periodicities present	Yes	Fourier transform
23	Chambers and Blackford (2001)	Wales	Brecon Beacons	51.866667	-3.388889	Red	Multi-decadal	Marginally dated for	Yes	Fourier transform

	Journal of Quaternary Science 16, 329-338.							periodicities present		
24	Chambers et al. (1997) The Holocene 7, 391-399.	Scotland	Talla Moss	55.458555	-3.352038	None	Multi-decadal	Satisfactorily dated for periodicities present	No	Simple periodogram
25	Oliver et al. (1997) Review of Palaeobotany and Palynology 96, 121-144	Scotland	Pickletillem	56.412496	-2.906660	None	Multi-decadal	Satisfactorily dated for periodicities present	Yes	Power spectra
26	Yu et al. (2003) The Holocene 13, 801–808.	Canada	Upper Pinto Fen	53.583333	-118.016667	White	Multi-centennial	Well dated for periodicities present	Yes	Lomb-Scargle

7 Correlation analysis

Table shows results of bivariate correlation analysis between the proxy data and solar variability, and random walk data and solar variability. Yellow highlights indicate where significance at the 0.01 level was found. Orange highlights indicate where significance at the 0.05 level was found.

[GH = Great Heath; MT = Malham Tarn Moss; BD = Ballyduff; BB = Butterburn Flow; DI = Dead Island; SL = Slieveanorra; DE = Derragh; SI = Sidney; MI = Minden]

Graphs show running correlation analysis to determine the temporal variation of the correlation. Top panel shows the sunspot number (*n*; black line) and standardised proxy-based water table reconstruction (STD WTD units; red line). The lower panel shows the running correlation. The first set of graphs show time windows = 100 years, and the second set of graphs show the analysis repeated with time windows = 500 years.

Real data

			sol
Spearman's rho	GH	Correlation Coefficient	-.196**
		Sig. (2-tailed)	.000
		N	507
	MT	Correlation Coefficient	.246**
		Sig. (2-tailed)	.000
		N	243
	BD	Correlation Coefficient	.412**
		Sig. (2-tailed)	.000
		N	476
	BB	Correlation Coefficient	-.043
		Sig. (2-tailed)	.331
		N	507
	DI	Correlation Coefficient	.142**
		Sig. (2-tailed)	.003
		N	446
	SL	Correlation Coefficient	.037
		Sig. (2-tailed)	.455
		N	419
	DE	Correlation Coefficient	-.060
		Sig. (2-tailed)	.178
		N	507
	SI	Correlation Coefficient	.062
		Sig. (2-tailed)	.289
		N	297
	MI	Correlation Coefficient	.057
		Sig. (2-tailed)	.308
		N	324

**. Correlation is significant at the 0.01 level (2-tailed).

Random walks

		sol
GH RW	Correlation Coefficient	.428**
	Sig. (2-tailed)	.000
	N	507
MT RW	Correlation Coefficient	.205**
	Sig. (2-tailed)	.001
	N	243
BD RW	Correlation Coefficient	-.067
	Sig. (2-tailed)	.143
	N	476
BB RW	Correlation Coefficient	-.055
	Sig. (2-tailed)	.213
	N	507
DI RW	Correlation Coefficient	.023
	Sig. (2-tailed)	.631
	N	446
SL RW	Correlation Coefficient	.096
	Sig. (2-tailed)	.051
	N	419
DE RW	Correlation Coefficient	-.097*
	Sig. (2-tailed)	.028
	N	507
SI RW	Correlation Coefficient	-.210**
	Sig. (2-tailed)	.000
	N	297
MI RW	Correlation Coefficient	.047
	Sig. (2-tailed)	.395
	N	324

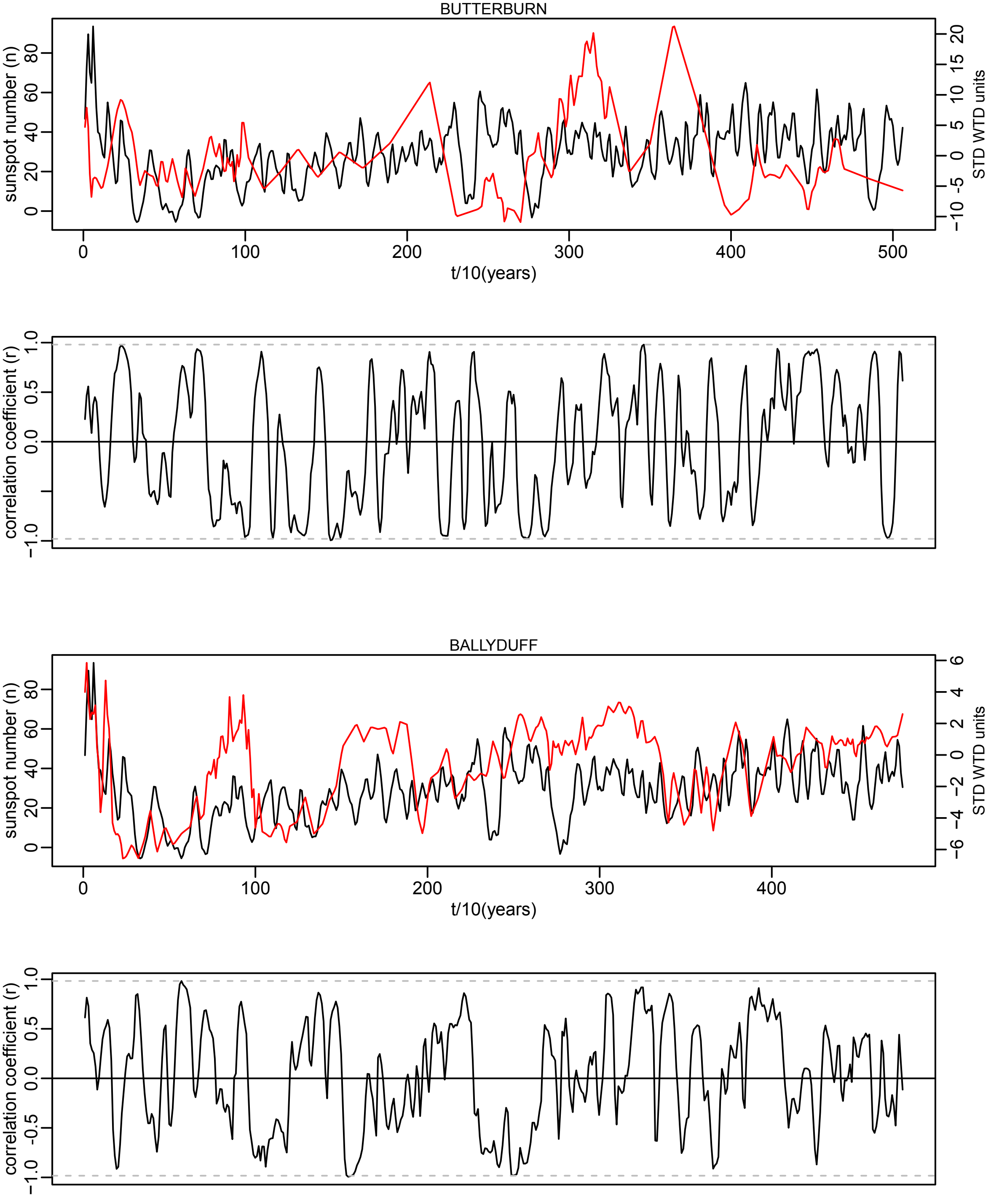
BUTTERBURN
sunspot number (n)
STD WTD units
t/10(years)
correlation coefficient (r)
BALLYDUFF
sunspot number (n)
STD WTD units
t/10(years)
correlation coefficient (r)

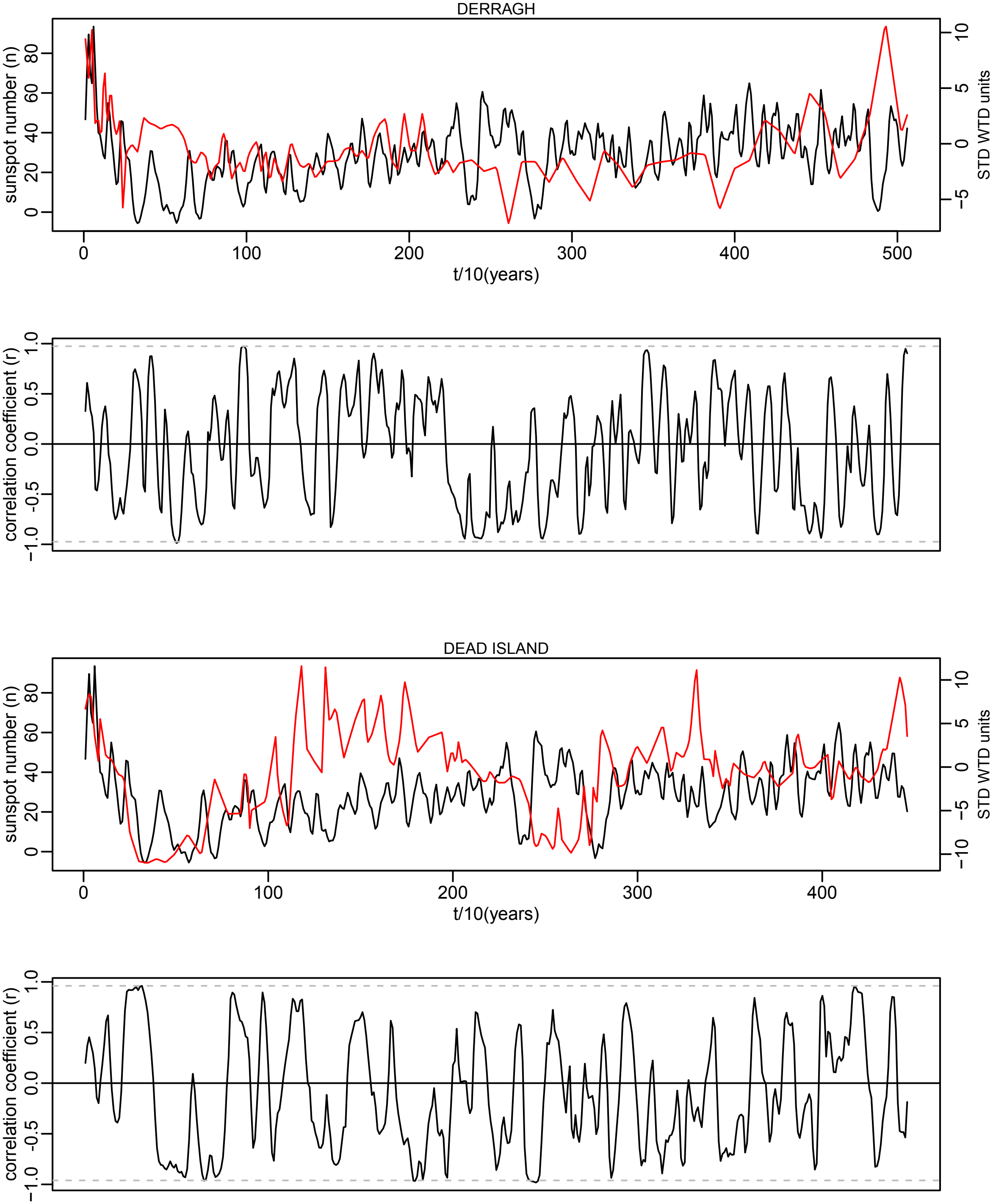
DERRAGH
sunspot number (n)
0
20
40
60
80
STD WTD units
−5
0
5
10
0
100
200
300
400
500
t/10(years)
correlation coefficient (r)
−1.0
−0.5
0.0
0.5
1.0
DEAD ISLAND
sunspot number (n)
0
20
40
60
80
STD WTD units
−10
−5
0
5
10
0
100
200
300
400
t/10(years)
correlation coefficient (r)
−1.0
−0.5
0.0
0.5
1.0

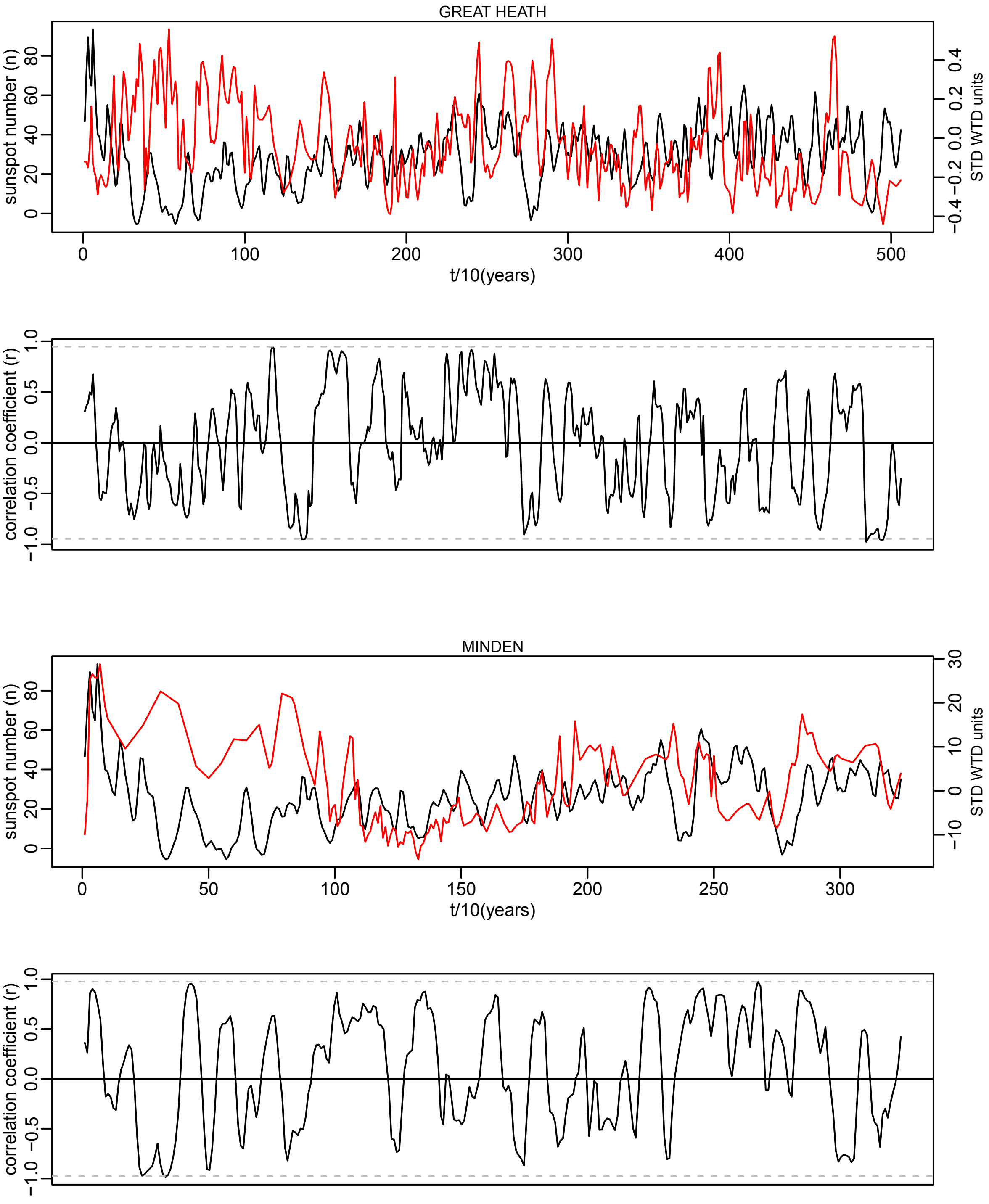
GREAT HEATH
sunspot number (n)
STD WTD units
t/10(years)
correlation coefficient (r)
MINDEN
sunspot number (n)
STD WTD units
t/10(years)
correlation coefficient (r)

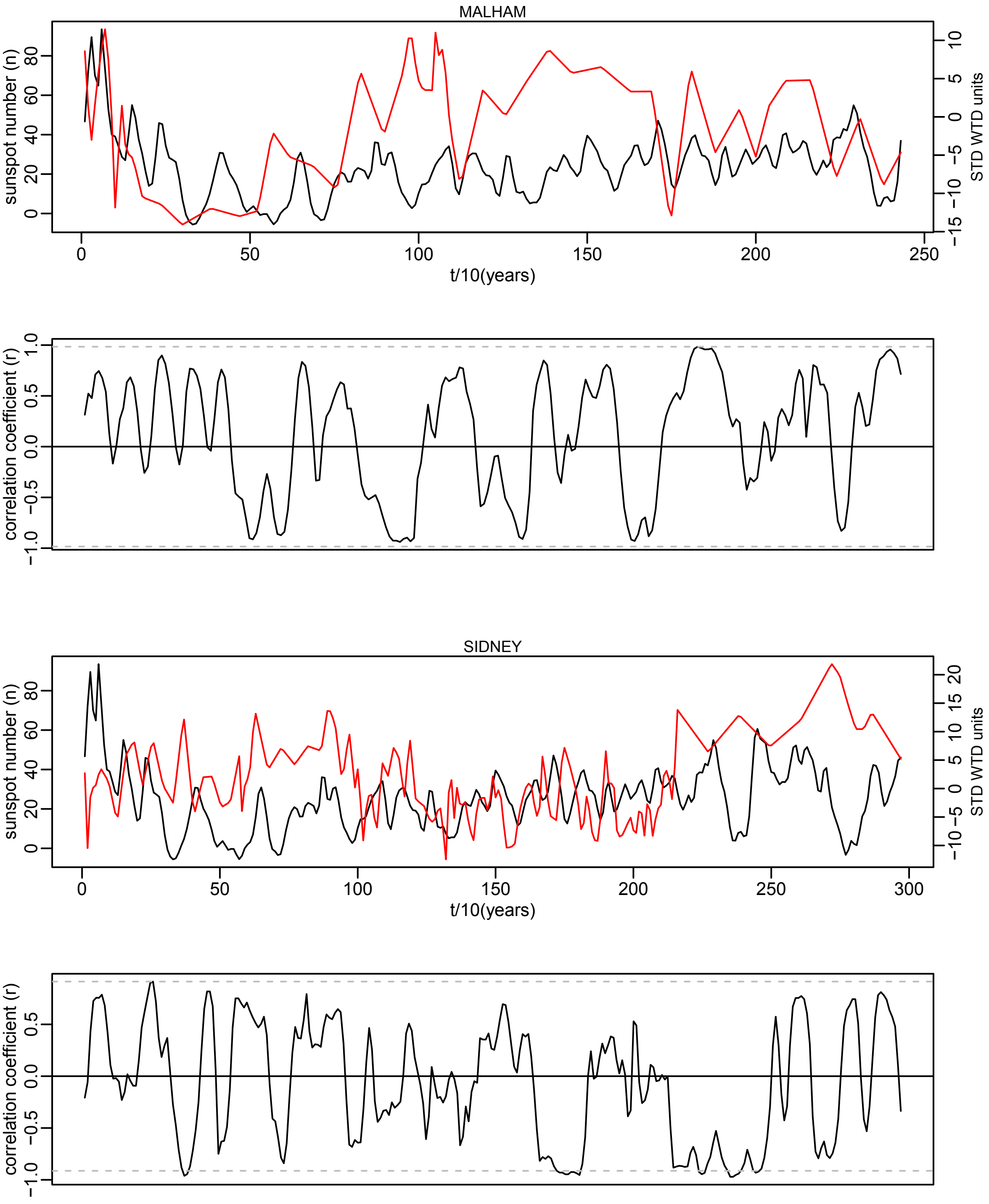

MALHAM
sunspot number (n)
STD WTD units
t/10(years)
correlation coefficient (r)
SIDNEY
sunspot number (n)
STD WTD units
t/10(years)
correlation coefficient (r)

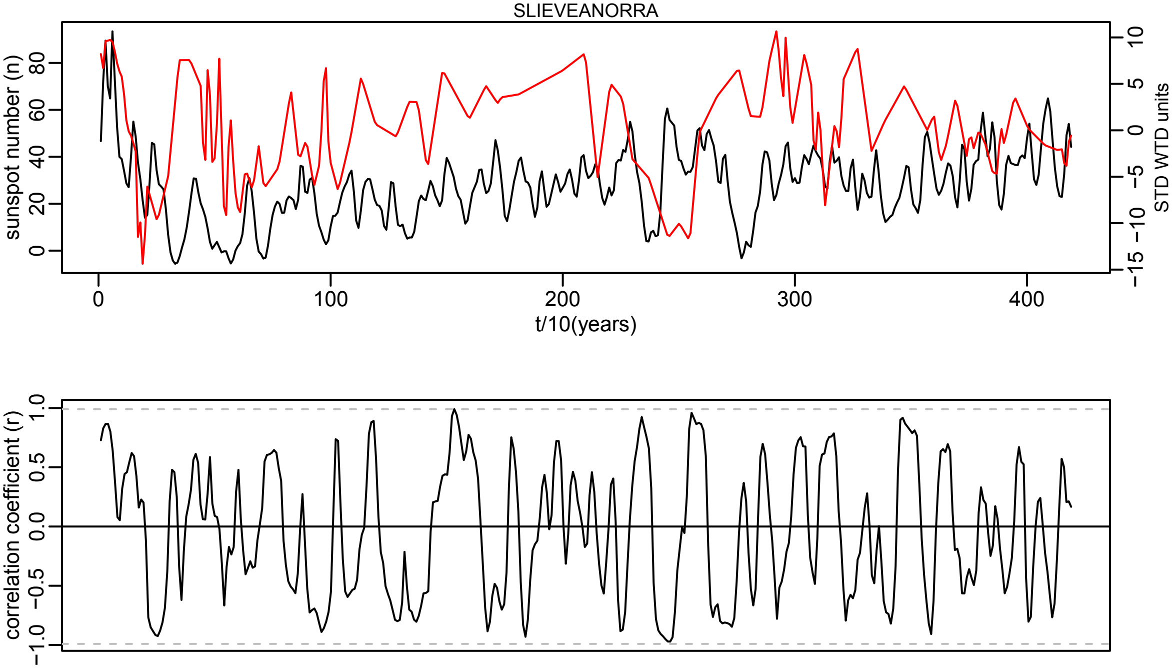

SLIEVEANORRA
sunspot number (n)
0
20
40
60
80
STD WTD units
−15
−10
−5
0
5
10
0
100
200
300
400
t/10(years)
correlation coefficient (r)
−1.0
−0.5
0.0
0.5
1.0

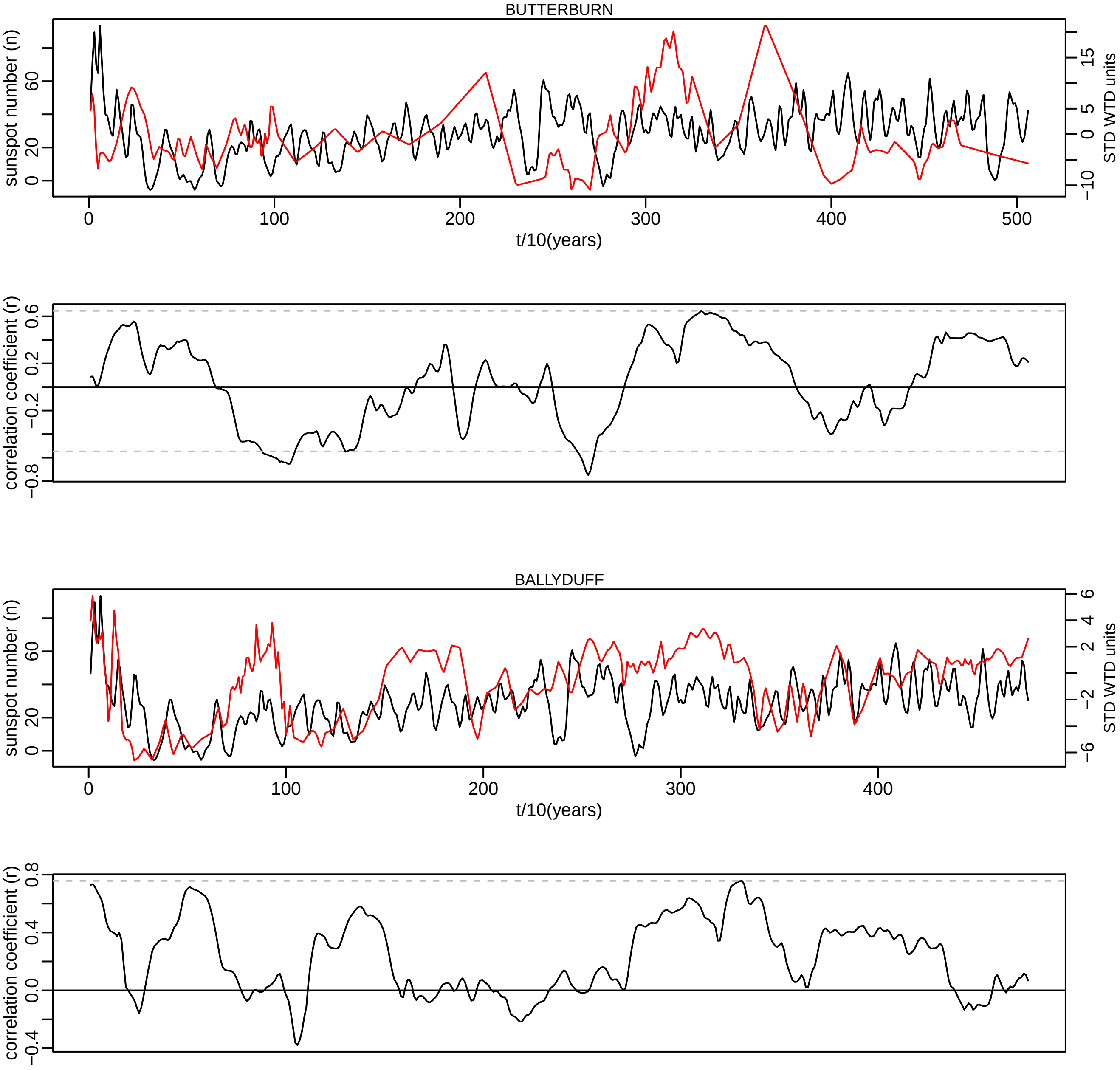
BUTTERBURN
sunspot number (n)
STD WTD units
t/10(years)
correlation coefficient (r)
BALLYDUFF
sunspot number (n)
STD WTD units
t/10(years)
correlation coefficient (r)

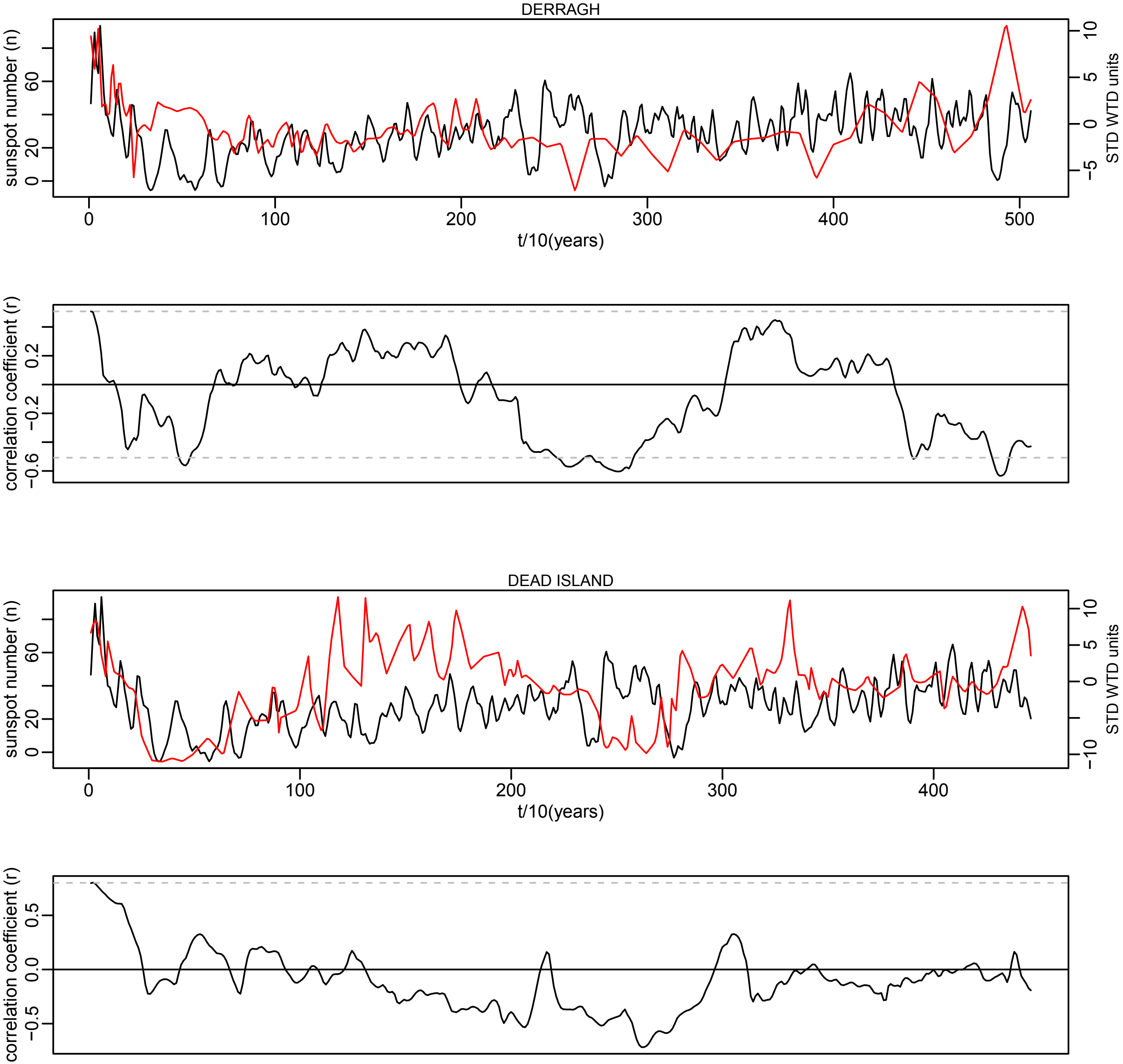
DERRAGH
sunspot number (n)
STD WTD units
t/10(years)
correlation coefficient (r)
DEAD ISLAND
sunspot number (n)
STD WTD units
t/10(years)
correlation coefficient (r)

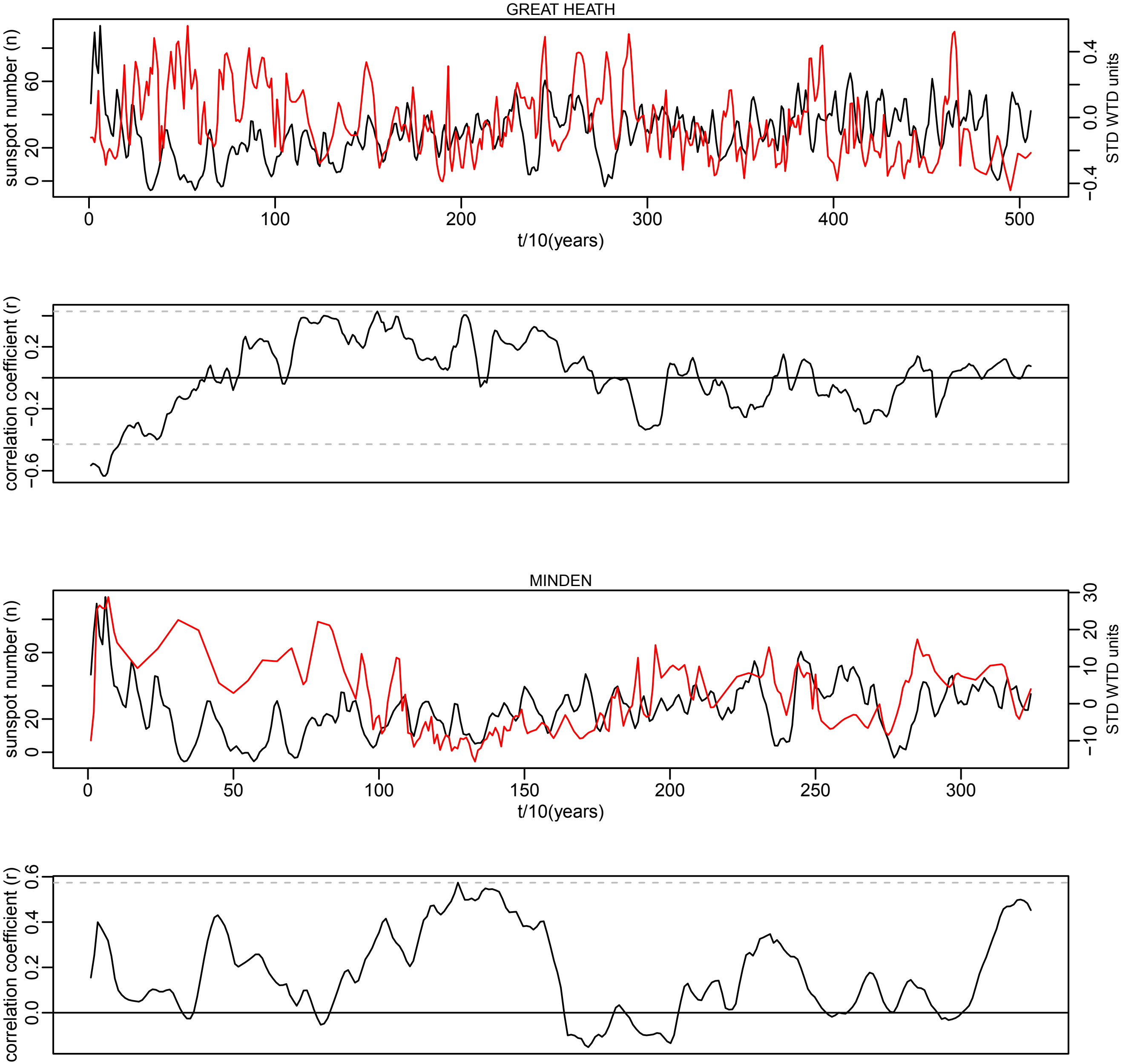
GREAT HEATH
sunspot number (n)
STD WTD units
t/10(years)
correlation coefficient (r)
MINDEN
sunspot number (n)
STD WTD units
t/10(years)
correlation coefficient (r)

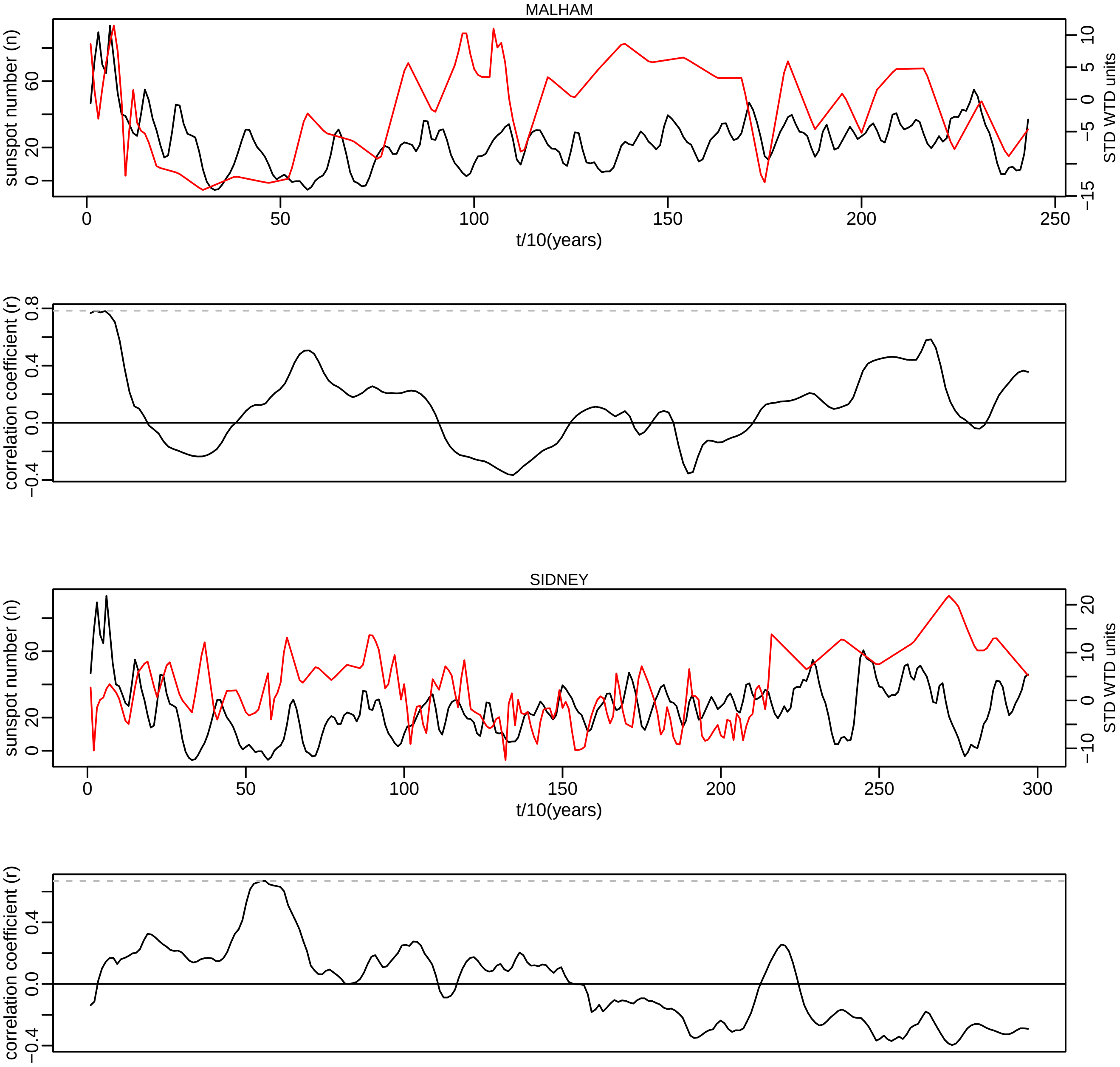
MALHAM
sunspot number (n)
STD WTD units
t/10(years)
correlation coefficient (r)
SIDNEY
sunspot number (n)
STD WTD units
t/10(years)
correlation coefficient (r)

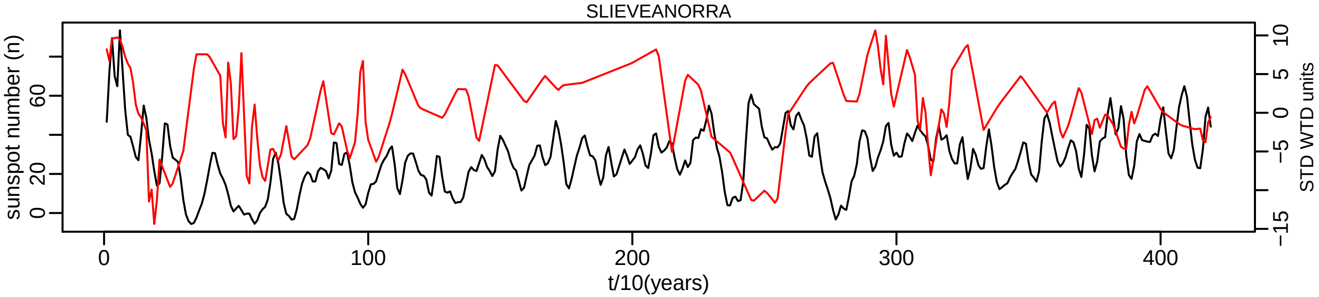
SLIEVEANORRA
sunspot number (n)
0
20
60
STD WTD units
10
5
0
−5
−15
0
100
200
300
400
t/10(years)

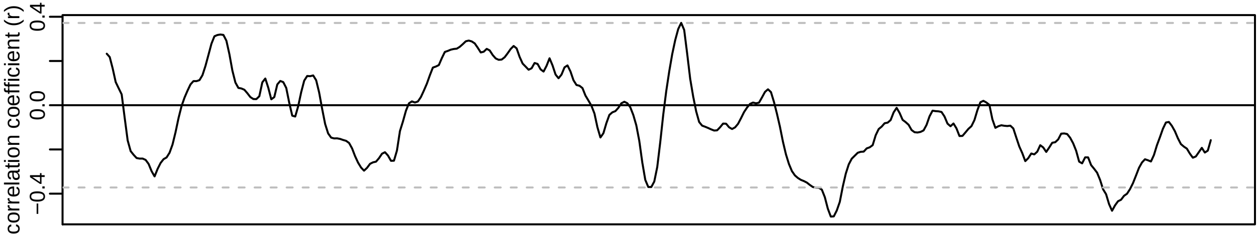
correlation coefficient (r)
0.4
0.0
−0.4

8 R code for running correlation analysis & random walks

Running correlation analysis

```
#Setup up analysis and files
library(gtools)
setwd("C:/Users/geogts/Desktop/Ed")
par(mar=c(3,3,2,3),mgp=c(1.5,.5,0), mfrow=c(4,1))
a<-read.csv(file="BB.csv",head=TRUE, na.strings="NA")
b<-read.csv(file="BD.csv",head=TRUE, na.strings="NA")
c<-read.csv(file="DE.csv",head=TRUE, na.strings="NA")
d<-read.csv(file="DI.csv",head=TRUE, na.strings="NA")
e<-read.csv(file="GH.csv",head=TRUE, na.strings="NA")
f<-read.csv(file="MI.csv",head=TRUE, na.strings="NA")
g<-read.csv(file="MT.csv",head=TRUE, na.strings="NA")
h<-read.csv(file="SI.csv",head=TRUE, na.strings="NA")
i<-read.csv(file="SL.csv",head=TRUE, na.strings="NA")


#Monte Carlo significance testing
#Monte Carlo as classical testing will not give the correct
significance levels for a running correlation because of the
multiple comparison problem.
res<-replicate(1000, {
  y<-rnorm(100)
  z<-max(running(a$sol,a$BB, fun=cor, width=10))# find maximum
correlation
  })
quantile(res, 0.90) #critical of maximum correlation at p=0.10
one-sided test.
```

```
#Monte Carlo as classical testing will not give the correct significance levels for a running correlation because of the multiple comparison problem.

res<-replicate(1000, {

  y<-rnorm(100)

  z<-max(running(b$sol,b$BD, fun=cor, width=10))# find maximum correlation

  })

quantile(res, 0.90) #critical of maximum correlation at p=0.10 one-sided test.

#Monte Carlo as classical testing will not give the correct significance levels for a running correlation because of the multiple comparison problem.

res<-replicate(1000, {

  y<-rnorm(100)

  z<-max(running(c$sol,c$DE, fun=cor, width=10))# find maximum correlation

  })

quantile(res, 0.90) #critical of maximum correlation at p=0.10 one-sided test.

#Monte Carlo as classical testing will not give the correct significance levels for a running correlation because of the multiple comparison problem.

res<-replicate(1000, {

  y<-rnorm(100)

  z<-max(running(d$sol,d$DI, fun=cor, width=10))# find maximum correlation

  })

quantile(res, 0.90) #critical of maximum correlation at p=0.10 one-sided test.

#Monte Carlo as classical testing will not give the correct significance levels for a running correlation because of the multiple comparison problem.

res<-replicate(1000, {

  y<-rnorm(100)
```

```
  z<-max(running(e$sol,e$GH, fun=cor, width=10))# find maximum
correlation

  })

quantile(res, 0.90) #critical of maximum correlation at p=0.10
one-sided test.

#Monte Carlo as classical testing will not give the correct
significance levels for a running correlation because of the
multiple comparison problem.

res<-replicate(1000, {

  y<-rnorm(100)

  z<-max(running(f$sol,f$MI, fun=cor, width=10))# find maximum
correlation

  })

quantile(res, 0.90) #critical of maximum correlation at p=0.10
one-sided test.

#Monte Carlo as classical testing will not give the correct
significance levels for a running correlation because of the
multiple comparison problem.

res<-replicate(1000, {

  y<-rnorm(100)

  z<-max(running(g$sol,g$MT, fun=cor, width=10))# find maximum
correlation

  })

quantile(res, 0.90) #critical of maximum correlation at p=0.10
one-sided test.

#Monte Carlo as classical testing will not give the correct
significance levels for a running correlation because of the
multiple comparison problem.

res<-replicate(1000, {

  y<-rnorm(100)

  z<-max(running(h$sol,h$SI, fun=cor, width=10))# find maximum
correlation

  })

quantile(res, 0.90) #critical of maximum correlation at p=0.10
one-sided test.
```

```
#Monte Carlo as classical testing will not give the correct significance levels for a running correlation because of the multiple comparison problem.

res<-replicate(1000, {

  y<-rnorm(100)

  z<-max(running(i$sol,i$SL, fun=cor, width=10))# find maximum correlation

  })

quantile(res, 0.90) #critical of maximum correlation at p=0.10 one-sided test.



#Running correlation analysis

###################################1

sol<-a$sol

prox<-a$BB

#plot time series

plot(sol, type="l", ylab="sunspot number (n)", xlab="t/10(years)")

par(new=T)# treat the graph window as a new window, so a new graph can be plotted without erasing the old

plot(prox, col=2, ann=F, type="l", axes=F)

axis(4)

mtext(side=4, text="STD WTD units", cex=0.6, line=1.5)

mtext(top=0, text="BUTTERBURN", cex=0.6, line=0)

#calculate the running correlation

rc<-running(sol,prox,fun=cor, width=10)

plot(rc, type="l", xlab="", ylab="correlation coefficient (r)",xaxt="n")

abline(h = 0, col = "black")

abline(h = 0.98, col = "grey", lty=2)

abline(h = -0.98, col = "grey", lty=2)

#

sol<-b$sol
```

```
prox<-b$BD

#plot time series

plot(sol, type="l", ylab="sunspot number (n)",
xlab="t/10(years)")

par(new=T)# treat the graph window as a new window, so a new
graph can be plotted without erasing the old

plot(prox, col=2, ann=F, type="l", axes=F)

axis(4)

mtext(side=4, text="STD WTD units", cex=0.6, line=1.5)

mtext(top=0, text="BALLYDUFF", cex=0.6, line=0)

#calculate the running correlation

rc<-running(sol,prox,fun=cor, width=10)

plot(rc, type="l", xlab="", ylab="correlation coefficient
(r)",xaxt="n")

abline(h = 0, col = "black")

abline(h = 0.983, col = "grey", lty=2)

abline(h = -0.983, col = "grey", lty=2)

###################################1


###################################2

sol<-c$sol

prox<-c$DE

#plot time series

plot(sol, type="l", ylab="sunspot number (n)",
xlab="t/10(years)")

par(new=T)# treat the graph window as a new window, so a new
graph can be plotted without erasing the old

plot(prox, col=2, ann=F, type="l", axes=F)

axis(4)

mtext(side=4, text="STD WTD units", cex=0.6, line=1.5)

mtext(top=0, text="DERRAGH", cex=0.6, line=0)

#calculate the running correlation

rc<-running(sol,prox,fun=cor, width=10)
```

```
plot(rc, type="l", xlab="", ylab="correlation coefficient (r)",xaxt="n")

abline(h = 0, col = "black")

abline(h = 0.974, col = "grey", lty=2)

abline(h = -0.974, col = "grey", lty=2)

#

sol<-d$sol

prox<-d$DI

#plot time series

plot(sol, type="l", ylab="sunspot number (n)", xlab="t/10(years)")

par(new=T)# treat the graph window as a new window, so a new graph can be plotted without erasing the old

plot(prox, col=2, ann=F, type="l", axes=F)

axis(4)

mtext(side=4, text="STD WTD units", cex=0.6, line=1.5)

mtext(top=0, text="DEAD ISLAND", cex=0.6, line=0)

#calculate the running correlation

rc<-running(sol,prox,fun=cor, width=10)

plot(rc, type="l", xlab="", ylab="correlation coefficient (r)",xaxt="n")

abline(h = 0, col = "black")

abline(h = 0.961, col = "grey", lty=2)

abline(h = -0.961, col = "grey", lty=2)

###################################2


###################################3

sol<-e$sol

prox<-e$GH

#plot time series

plot(sol, type="l", ylab="sunspot number (n)", xlab="t/10(years)")

par(new=T)# treat the graph window as a new window, so a new graph can be plotted without erasing the old
```

```
plot(prox, col=2, ann=F, type="l", axes=F)

axis(4)

mtext(side=4, text="STD WTD units", cex=0.6, line=1.5)

mtext(top=0, text="GREAT HEATH", cex=0.6, line=0)

#calculate the running correlation

rc<-running(sol,prox,fun=cor, width=10)

plot(rc, type="l", xlab="", ylab="correlation coefficient (r)",xaxt="n")

abline(h = 0, col = "black")

abline(h = 0.947, col = "grey", lty=2)

abline(h = -0.947, col = "grey", lty=2)

#

sol<-f$sol

prox<-f$MI

#plot time series

plot(sol, type="l", ylab="sunspot number (n)", xlab="t/10(years)")

par(new=T)# treat the graph window as a new window, so a new graph can be plotted without erasing the old

plot(prox, col=2, ann=F, type="l", axes=F)

axis(4)

mtext(side=4, text="STD WTD units", cex=0.6, line=1.5)

mtext(top=0, text="MINDEN", cex=0.6, line=0)

#calculate the running correlation

rc<-running(sol,prox,fun=cor, width=10)

plot(rc, type="l", xlab="", ylab="correlation coefficient (r)",xaxt="n")

abline(h = 0, col = "black")

abline(h = 0.977, col = "grey", lty=2)

abline(h = -0.977, col = "grey", lty=2)

###################################3


###################################4
```

```
sol<-g$sol

prox<-g$MT

#plot time series

plot(sol, type="l", ylab="sunspot number (n)",
xlab="t/10(years)")

par(new=T)# treat the graph window as a new window, so a new
graph can be plotted without erasing the old

plot(prox, col=2, ann=F, type="l", axes=F)

axis(4)

mtext(side=4, text="STD WTD units", cex=0.6, line=1.5)

mtext(top=0, text="MALHAM", cex=0.6, line=0)

#calculate the running correlation

rc<-running(sol,prox,fun=cor, width=10)

plot(rc, type="l", xlab="", ylab="correlation coefficient
(r)",xaxt="n")

abline(h = 0, col = "black")

abline(h = 0.984, col = "grey", lty=2)

abline(h = -0.984, col = "grey", lty=2)

#

sol<-h$sol

prox<-h$SI

#plot time series

plot(sol, type="l", ylab="sunspot number (n)",
xlab="t/10(years)")

par(new=T)# treat the graph window as a new window, so a new
graph can be plotted without erasing the old

plot(prox, col=2, ann=F, type="l", axes=F)

axis(4)

mtext(side=4, text="STD WTD units", cex=0.6, line=1.5)

mtext(top=0, text="SIDNEY", cex=0.6, line=0)

#calculate the running correlation

rc<-running(sol,prox,fun=cor, width=10)

plot(rc, type="l", xlab="", ylab="correlation coefficient
(r)",xaxt="n")
```

```
abline(h = 0, col = "black")

abline(h = 0.913, col = "grey", lty=2)

abline(h = -0.913, col = "grey", lty=2)

###################################4


###################################5

sol<-i$sol

prox<-i$SL

#plot time series

plot(sol, type="l", ylab="sunspot number (n)", xlab="t/10(years)")

par(new=T)# treat the graph window as a new window, so a new graph can be plotted without erasing the old

plot(prox, col=2, ann=F, type="l", axes=F)

axis(4)

mtext(side=4, text="STD WTD units", cex=0.6, line=1.5)

mtext(top=0, text="SLIEVEANORRA", cex=0.6, line=0)

#calculate the running correlation

rc<-running(sol,prox,fun=cor, width=10)

plot(rc, type="l", xlab="", ylab="correlation coefficient (r)",xaxt="n")

abline(h = 0, col = "black")

abline(h = 0.991, col = "grey", lty=2)

abline(h = -0.991, col = "grey", lty=2)

###################################5
```

Random walks

```
# Generate k random walks across time {0, 10, ... , T}
T <- 3235
k <- 15
initial.value <- 0
GetRandomWalk <- function() {
  # Add a standard normal at each step
  initial.value + c(0, cumsum(rnorm(T)))
}
# Matrix of random walks
values <- replicate(k, GetRandomWalk())
# Create an empty plot
dev.new(height=8, width=12)
plot(0:T, rep(NA, T + 1), main=sprintf("%s Random Walks", k),
     xlab="time", ylab="value",
     ylim=10 + 3.5 * c(-1, 1) * sqrt(T))
mtext(sprintf("%s%s} with initial value of %s",
              "Across time {0, 1, ... , ", T, initial.value))
for (i in 1:k) {
  lines(0:T, values[ , i], lwd=0.25)
}
for (sign in c(-1, 1)) {

  curve(initial.value + sign * 1.96 * sqrt(x), from=0, to=T,
        n=2*T, col="darkred", lty=2, lwd=1.5, add=TRUE)
}
```

9 Random walk-solar reconstruction correlations

Results of correlation analysis (Spearman's Rank, $p < 0.05$) of the 5000 random walks with the solar reconstruction of Solanki et al. (2004). Blue line = Spearman's R_S; red line = p-value.

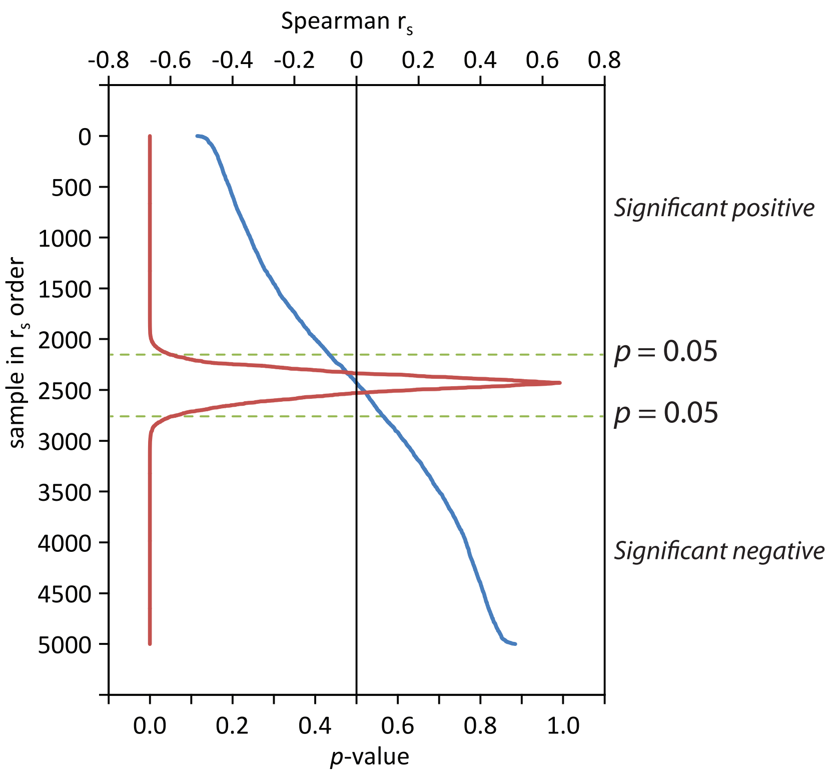

Spearman r_s
-0.8
-0.6
-0.4
-0.2
0
0.2
0.4
0.6
0.8
sample in r_s order
0
500
1000
1500
2000
2500
3000
3500
4000
4500
5000
Significant positive
$p = 0.05$
$p = 0.05$
Significant negative
0.0
0.2
0.4
0.6
0.8
1.0
p-value
